# A systematic CRISPR screen reveals redundant and specific roles for Dscam1 isoform diversity in neuronal wiring

**DOI:** 10.1371/journal.pbio.3002197

**Published:** 2023-07-06

**Authors:** Haiyang Dong, Xi Yang, Lili Wu, Shixin Zhang, Jian Zhang, Pengjuan Guo, Yiwen Du, Changkun Pan, Ying Fu, Lei Li, Jilong Shi, Yanda Zhu, Hongru Ma, Lina Bian, Bingbing Xu, Guo Li, Feng Shi, Jianhua Huang, Haihuai He, Yongfeng Jin

**Affiliations:** 1 MOE Laboratory of Biosystems Homeostasis & Protection and Innovation Center for Cell Signaling Network, College of Life Sciences, Zhejiang University, Hangzhou, Zhejiang, China; 2 Cancer Center and State Key Laboratory of Biotherapy, West China Hospital, Sichuan University, Chengdu, China; 3 Institute of Insect Sciences, Zhejiang University, Hangzhou, Zhejiang, China, PR China; 4 Department of Neurosurgery, Cancer Center and State Key Laboratory of Biotherapy, West China Hospital, Sichuan University, Chengdu, China; University of Michigan, UNITED STATES

## Abstract

*Drosophila melanogaster Down syndrome cell adhesion molecule* 1 (*Dscam1*) encodes 19,008 diverse ectodomain isoforms via the alternative splicing of exon 4, 6, and 9 clusters. However, whether individual isoforms or exon clusters have specific significance is unclear. Here, using phenotype–diversity correlation analysis, we reveal the redundant and specific roles of Dscam1 diversity in neuronal wiring. A series of deletion mutations were performed from the endogenous locus harboring exon 4, 6, or 9 clusters, reducing to 396 to 18,612 potential ectodomain isoforms. Of the 3 types of neurons assessed, dendrite self/non-self discrimination required a minimum number of isoforms (approximately 2,000), independent of exon clusters or isoforms. In contrast, normal axon patterning in the mushroom body and mechanosensory neurons requires many more isoforms that tend to associate with specific exon clusters or isoforms. We conclude that the role of the Dscam1 diversity in dendrite self/non-self discrimination is nonspecifically mediated by its isoform diversity. In contrast, a separate role requires variable domain- or isoform-related functions and is essential for other neurodevelopmental contexts, such as axonal growth and branching. Our findings shed new light on a general principle for the role of Dscam1 diversity in neuronal wiring.

## Abstract 2

The fruit fly Down syndrome cell adhesion molecule 1 (Dscam1) encodes 19,008 diverse ectodomain isoforms via alternative splicing. A systematic CRISPR screen of Drosophila Dscam1 reveals both redundant and specific functions of its isoforms in neuronal wiring.

## Introduction

Diverse families of cell surface molecules play important roles in cell recognition during the assembly of neural circuits [1–3]. In *Drosophila melanogaster*, the *Dscam1* gene can generate 38,016 closely related single-transmembrane proteins of the immunoglobulin superfamily through mutually exclusive alternative splicing of 12 exon 4s, 48 exon 6s, 33 exon 9s, and 2 exon 17s, comprising 19,008 ectodomains linked to one of 2 alternative transmembrane segments [4]. Since discovering Dscam1’s extraordinary diversity, their specific features have fascinated and perplexed interdisciplinary researchers in molecular biology and neurobiology. Biochemical analyses show that each ectodomain preferentially exhibits isoform-specific homophilic binding; each isoform binds only to itself but binds weakly or not to other isoforms in vitro and in vivo [5,6]. Each neuron is estimated to express approximately 10 to 50 Dscam1 isoforms via probabilistic splicing, providing each neuron with a unique molecular code for self-recognition in the nervous system [7–10]. *Dscam1* is required for wiring of diverse neurons [11–22]. A canonical model suggests that isoform-specific homophilic binding of identical Dscam1 receptor isoforms on sister branches initiates neurite repulsion, which is self-avoidance [9]. In this model, a single Dscam1 isoform is sufficient to ensure the detachment of sister self-dendrites from the same neuron [11,12,23]. However, to ensure that different neurons possess a specific identity, thousands of Dscam1 isoforms are essential and sufficient to discriminate between self and non-self neurites [24]. In this canonical model, the number of Dscam1 isoforms matter, and the specific isoform may not be functionally important.

However, there is growing evidence that Dscam1 has additional functions beyond self-avoidance. Genetic analysis of mutant flies lacking all but one of exon 6 shows that Dscam1 diversity is intrinsically required by cells for the collateral formation of mechanosensory (MS) axons [18]. In addition, genetic analysis of *Dscam1* RNA interference (RNAi) knockdown indicates that Dscam1 is essential for forming new dendritic branches in *Drosophila* motoneurons but not for dendritic spacing [25]. Recent knockdown of Dscam1 revealed its diverse roles in central neuron arbor differentiation [26]. Recently, we showed that alterations of *Dscam1* exon 9 variants result in obvious mushroom body (MB) axonal defects, partially supporting the specific function of Dscam1 isoforms [27,28]. Genetic analysis of a series of mutant flies with a single exon 6 or 9 variant revealed that 396 and 576 Dscam1 isoforms are insufficient for normal patterning of MB axonal branches [22]. All these data suggest that self-avoidance alone does not explain the function of Dscam1 in neuronal wiring.

Moreover, most functional genetics experiments have focused on Dscam1 loss-of-function mutations and RNAi knockdown itself or used overexpression of certain isoforms [11–14,19,25,26,29–31]. Only a few examples involve the effect of Dscam1 diversity on neuronal phenotypes, which mainly harbor the deletion of relatively small exon 4 clusters [17,21,24]. Other studies have only examined the effect of the remaining single exons 6 or 9 on neuronal wiring [18,24]. Recent studies have shown that mutant flies with single exon 4, 6, or 9 variants exhibited varying developmental defects [22]. However, intermediate information between mutants with a single exon and the wild type is lacking. Furthermore, the diverse potential functions of Dscam1 isoforms have not been assessed in parallel. Therefore, it remains unknown to what extent different phenotypic defects in Dscam1 mutants correlate with the number of isoforms and how isoform diversity and specificity contribute to distinct phenotypes. The differential significance of exon 4, 6, and 9 clusters remains unknown, as does the specificity of Dscam1 isoforms. A systematic comparative analysis via gradient reduction of exon 4, 6, or 9 clusters from the endogenous *Dscam1* locus will allow meaningful comparison of isoform diversity–function correlations.

This study used CRISPR/Cas9 to perform a series of deletions from the endogenous locus harboring exon 4, 6, or 9 clusters, encoding 396 to 18,612 potential ectodomain isoforms. We systematically evaluated the phenotypes of 3 classes of neurons. All phenotypes generally improved as the potential number of isoforms increased; however, the magnitude of defects varied remarkably in a variable cluster-specific manner. Dendrite self/non-self discrimination requires a minimum number of isoforms (approximately 2,000), independent of the isoform identity. In contrast, normal axon patterning in MBs and MS neurons requires up to 10,000 isoforms (at least some subsets), which tend to be associated with specific exon clusters or isoforms. We conclude that exon 4, 6, or 9 clusters share the same role of Dscam1 isoforms that provide each neuron with a unique identity, to distinguish self from non-self. In contrast, distinct and separate roles are essential for other aspects of neuronal development, such as axonal growth and branching, requiring a variable domain-related function. Our findings demonstrate that Dscam1 isoform diversity has different requirements for diverse neurons in a variable domain-specific manner and further highlight the functional significance of *Dscam1* multiclusters.

## Results

### Ectodomain-wide deletion of variable exons 4, 6, or 9 to reduce Dscam1 diversity

**Fig 1:**
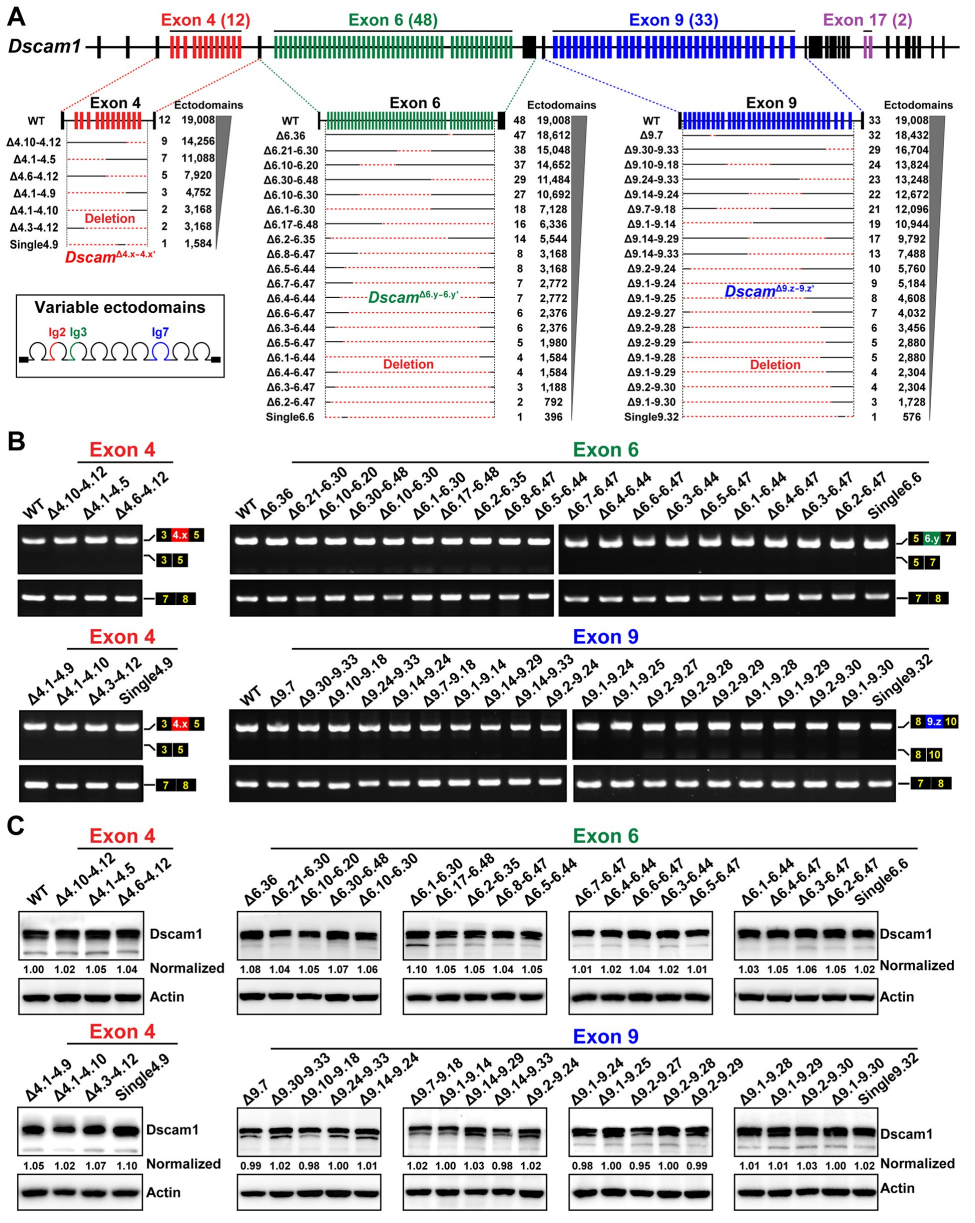
Construction and molecular characterization of *Dscam1* deletion mutants. **See also S1 Fig. (A)** Schematic diagrams of mutants with reduced Dscam1 diversity in exon 4, 6, or 9 clusters. Red dotted lines indicate the fragments of deleted variable exons. Potential ectodomain diversity is shown on the right. **(B)** RT-PCR diagrams of head tissues of *Dscam1* mutants. The overall inclusion frequency of variable exons in the mutants for each variable cluster was indistinguishable from the wild-type controls. **(C)** The protein levels in the head tissues of the *Dscam1* mutants were similar to the wild-type controls. Dscam1 levels were normalized to β-actin, and the expression levels were then compared to the wild type, set to 1. Dscam1, Down syndrome cell adhesion molecule 1; RT-PCR, reverse transcription PCR; WT, wild type.

We constructed a series of mutants using CRISPR/Cas9 technology in which various numbers of exons 4, 6, and 9 were differentially deleted (designated *Dscam*^Δ4.x-4.x’^, *Dscam*^Δ6.y-6.y’^, and *Dscam*^Δ9.z-9.z’^, respectively), to test the extent to which molecular diversity is required for Dscam1 to perform its diverse functions (Figs 1A and S1). The largest deletion removed 47 out of 48 exon 6 alternatives, encoding 396 potential isoforms, while the smallest deletion removed 1 out of 48 exon 6 alternatives, encoding 18,612 potential isoforms. These deletions resulted in a loss of endogenous loci harboring exon 4, 6, or 9 clusters from a single exon to a cluster scale, encoding 396 to 18,612 potential ectodomain isoforms (Fig 1A).

All these mutants were homozygous-viable, fertile, and morphologically normal externally compared to the wild-type controls. Reverse transcription PCR (RT-PCR) analyses did not show obvious skipping of variable exons 4, 6, and 9 in the tissues of these *Dscam1* mutant flies, like the wild-type control (Fig 1B), indicating that partial deletion of individual exon 4, 6, or 9 clusters do not have a significant effect on splicing of the remaining variable exons. A western blot assay showed no difference in the overall level of Dscam1 protein in these mutants compared to the wild type (Fig 1C). Thus, these fly mutants had reduced Dscam1 diversity to different degrees while maintaining overall expression control of the endogenous *Dscam1* gene. These fly mutants allow us to systematically assess in parallel to the phenotypic consequences of reducing the diversity of exons 4, 6 and 9 in different developmental contexts, including 3 classes of neurons. This experimental design can be used to identify the correlation between Dscam1 diversity and individual phenotypic defects and explore how many different isoforms are required for the normal wiring of distinct neurons.

### Differential requirements of exon 4, 6, and 9 diversities for fly viability

**Fig 2:**
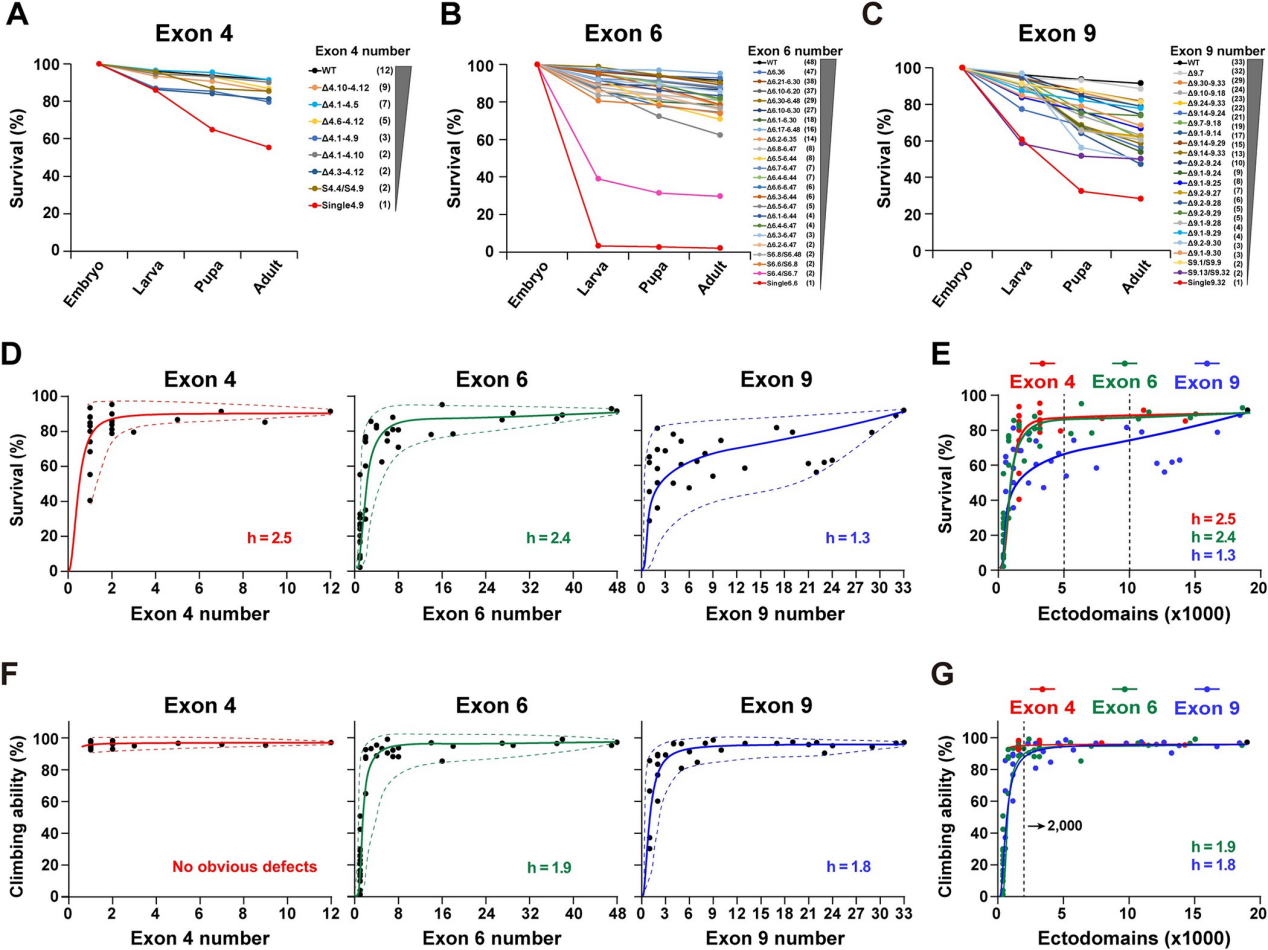
Reducing the Dscam1 diversity affects fly viability and locomotion ability. **See also S2 and S3 Figs. (A-C)** Survival rates at each development stage in the wild-type and mutants with reduced numbers of exon 4 (A), exon 6 (B), or exon 9 (C). The number of remaining variable exons is shown on the right. **(D)** Survival rates plotted against the remaining numbers of variable exon 4s, exon 6s, and exon 9s. Survival rates positively correlated with the number of variable exons, and the best-fitting Hill function corresponds to a sigmoidal curve. The Hill coefficient (h) is the sensitivity parameter. The dotted lines represent the spectra of phenotypic variations, i.e., the variation range of phenotype defects among different mutants with the same degree of Dscam1 diversity. The minimum phenotypic defects are shown by the upper dashed line, and the lower dashed line shows the maximum phenotypic defects. **(E)** Survival rates as a function of the number of ectodomain isoforms. The survival phenotype was more sensitive to exon 9 numbers (lower h) than to exon 4 and 6 numbers. **(F)** Locomotion ability of the fly adults correlated with the number of variable exons 6 and 9. Reducing to a single exon 4 did not affect the climbing ability of flies. **(G)** Locomotion ability of fly adults as a function of the number of the ectodomain isoforms. Data used to generate graphs can be found in S1 Data. Dscam1, Down syndrome cell adhesion molecule 1; WT, wild type.

First, we investigated the effect of reducing the number of exons 4, 6, and 9 on fly viability. Two hundred newly laid mutant eggs were collected in plates to assess the fertility of these mutants. After 48 h, the hatched eggs were counted. Reducing the exon 4 numbers had no significant effect on the survival rate from embryos to adults, except for a few *Dscam*^single4.x^ mutants (i.e., *Dscam*^Single4.9^) (Figs 2A and S2A). Similarly, the survival rate of most *Dscam*^Δ6.y-6.y’^ flies was slightly reduced. The survival rate increased steeply as the number of available exon 6s increased (Figs 2B and S2B). A less steep downward trend was observed when we performed a gradient deletion of the exon 9 cluster (Figs 2C and S2C). Plotting the mean survival rate as a function of the number of remaining exon 4s, 6s, and 9s revealed highly nonlinear and positive relationships, corresponding to a sigmoidal function (Fig 2D). These different curves are reminiscent of the Hill equation with different Hill (h) coefficients, which are generally used to characterize the sensitivity and cooperativity of the response in a biochemistry reaction [32]. Since the Hill equation can be flexibly fitted to the data of different Hill coefficients, we used the Hill equation to fit the sigmoid curve and the Hill coefficient to assess the sensitivity of reducing Dscam1 diversity on phenotypic defects.

We found that reducing exon clusters 4 and 6 had comparable effects on survival rates, as evidenced by their similar Hill values. In contrast, reducing exon 9s has a greater effect on the survival rate than reducing exon 4s or 6s, as evidenced by a much lower Hill value in exon 9 (2.5/2.8 versus 1.3) (Fig 2E). The data indicate that fly viability is more sensitive to reduced diversity in exon 9 than in exons 4 and 6. The data also suggest that diversity of exon clusters 4, 6, and 9 is differentially required for the normal viability of animals. In addition, most of the mutants with obviously reduced survival rates (i.e., *Dscam*^Single6.y^) had significantly reduced hatching compared to controls. In contrast, the pupation and eclosion rates were not significantly reduced, suggesting that the hatching process in these mutants is more sensitive to reduced Dscam1 diversity than pupation and eclosion. We speculate that Dscam1 diversity is more important in embryonic development, possibly affecting the embryonic nervous system. Indeed, reducing Dscam1 diversity led to obvious embryonic central nervous system defects [13].

Although the survival rate was positively correlated with the number of available exons, mutants with the same degree of diversity exhibited considerable variation. There was a broader spectrum of phenotypic variations for the exon 9 cluster than for exon 4 and 6 clusters (Figs 2D and S2). Since this variation among individual isoforms partially reflects the functional specificity of the various isoforms, these data suggest that exon 9 isoforms exhibit higher specificity than exon 4 and 6 isoforms.

### The effect of reducing exon 4, 6, and 9 diversities on fly locomotion

We performed a climbing assay on the mutant adults to assess locomotor abilities and assess the impact of reducing Dscam1 diversity on fly behavior. There was no significant reduction in locomotor ability after the deletion of some exon 4s in all the mutants, even in *Dscam*^Single4.x^ mutants lacking all but one exon 4 (Figs 2F and S3A). As the number of potential Dscam1 isoforms increased, the climbing ability increased steeply. When the number of exon 6s was increased to 3, the climbing ability of Dscam1 mutants was largely indistinguishable from the wild-type control (Figs 2F and S3B). Similarly, there was a strong positive correlation between exon 9 diversity and locomotion defects, with little difference between the various mutants (Figs 2F and S3C). The Hill index was similar between exons 6 and 9, indicating that reducing exon 6 or 9 diversity had the same effect on fly locomotion (Fig 2G). The correlation curve showed that the isoform diversity threshold for normal locomotion abilities was 1,500 to 2,000 using the system analyzed in this paper. The mutant flies exhibited normal locomotion abilities when the isoform diversity was over 1,500. Notably, this threshold is based only on this climbing assay, and more detailed behavior analyses remain to be conducted.

### Dscam1 diversity mediates dendrite self/non-self discrimination in an exon cluster- and isoform-independent manner

**Fig 3:**
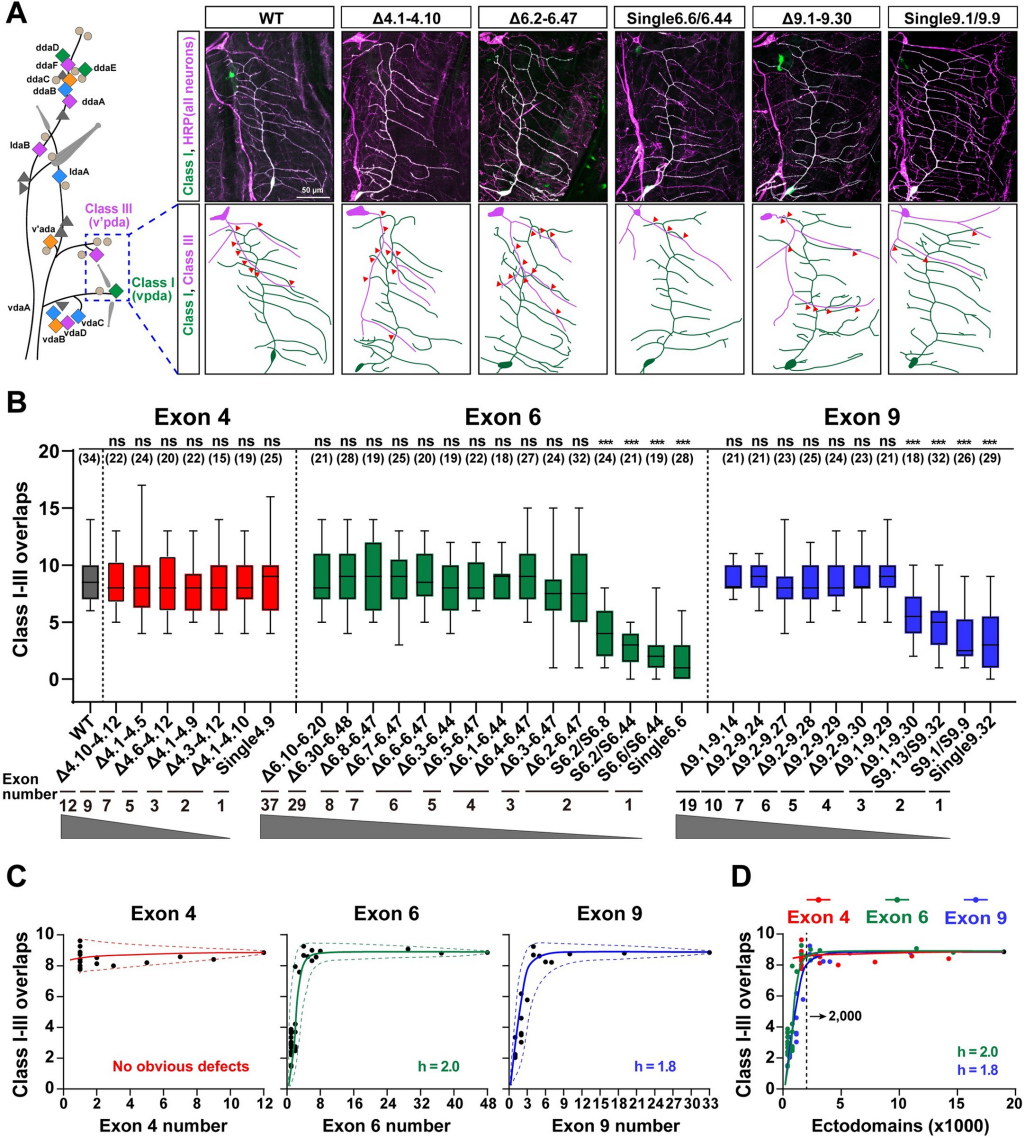
Reducing the Dscam1 diversity affects the overlaps between dendrites of class I and class III neurons. **See also S4 Fig. (A)** A schematic diagram of the distribution of 4 types of da neurons (colored diamonds) is shown on the left. The diagram of dendrite overlaps of class I and III neurons in the wild-type and deletion mutants is shown on the right. All neurons were detected by anti-HRP antibody (magenta), and class I (vpda) neurons were labeled with GFP driven by *Gal4*^*221*^ (green; appears white because they overlap with magenta). Red arrowheads indicate crossing between class I and class III dendrites. Scale bar, 50 μm. **(B)** Overlaps between class I and class III dendrites of da neurons of different Dscam1 mutant flies. ns, not significant; ***p < 0.001 (Student *t* test, two-tailed). The numbers in parentheses represent the analyzed class I and III neurons. **(C)** Overlaps between class I–III dendrites largely correlated with the number of available variable exons 4, 6, and 9. The best-fitting Hill function corresponds to a sigmoidal curve. The dashed curves indicate the spectra of variation among different variants. The small variation spectra suggest that these isoforms have low specificity for this phenotype. **(D)** A comparison of phenotype–diversity correlates among variable exon 4, exon 6, and exon 9 clusters. The Hill coefficient (h) is similar between exons 6 and 9, suggesting that Dscam1 isoforms act largely in a variable exon cluster–independent manner. Data used to generate graphs can be found in S1 Data. da, dendritic arborization; Dscam1, Down syndrome cell adhesion molecule 1; HRP, horseradish peroxidase; WT, wild type.

Previous genetic studies have shown that Dscam1 diversity is essential in fly neuronal wiring [11–13,23,24]. However, systematic genetic analyses are lacking due to technical challenges before the invention of CRISPR/Cas9. We evaluated the phenotype defects in 3 classes of neurons in the mutant flies to compare the contribution of exon 4, 6, and 9 diversities in neural development. First, we investigated the number of isoforms required for dendritic self-avoidance and self/non-self discrimination. The dendritic arborization (da) neurons of the fly larva are an ideal system for self-avoidance and self/non-self discrimination because these neurons elaborate dendrites in a two-dimensional array [33] (Fig 3A). In this system, self-dendrites of the same neuron do not overlap. In contrast, the dendrites between different da neurons normally overlap (Fig 3A). The self-dendrites of class I neurons in the larval body wall seldom overlapped in all the mutant flies with reducing exon 4, 6, and 9 diversities (Figs 3A and S4). Consistent with previous studies [11,12,23,24], these data indicate that expressing one or multiple isoforms is sufficient to support dendrite repulsion between self-branches from the same neuron.

We further assessed the extent of exon 4, 6, and 9 diversities required for normal overlap between the dendrites of class I and class III neurons. As expected, overlaps between class I and class III dendrites in all *Dscam*^Δ4.x-4.x’^ mutants encoding more than 1,584 isoforms were not significantly different from the wild-type control (Fig 3A and 3B). The *Dscam*^Δ6.4–6.47^ mutant encoding 1,584 isoforms showed no noticeable overlap defects between class I and class III dendrites, nor did the other *Dscam*^Δ6.y-6.y’^ mutants encoding more than 1,584 isoforms (Fig 3B). Even the *Dscam*^Δ6.3–6.47^ mutant encoding 1,188 potential isoforms had overlaps that were similar to the wild-type controls. However, dendrites of class I and class III neurons exhibited obvious avoidance in mutants encoding 396 and 576 isoforms (Fig 3A and 3B) [22]. This indicates that ectopic repulsion occurred between class I and III dendrites in these mutants when Dscam1 diversity was reduced below a certain threshold value. We plotted dendritic overlaps as a function of the number of available exon 6s and revealed a highly nonlinear correlation to fit a sigmoidal curve (Fig 3C). In the same way, class I and III dendritic overlaps generally improved in a nonlinear manner as the number of remaining exon 9s increased (Fig 3C).

Furthermore, reducing exon clusters 4, 6, and 9 had a comparable effect on dendritic overlaps, as evidenced by their similar Hill values (Fig 3D). These data suggest that Dscam1 mediates dendritic overlapping independently of the variable exon clusters. Furthermore, different mutants with the same degree of diversity showed very limited variation, indicating that Dscam1 isoforms engage in this process in a largely independent manner from the isoform identity (Fig 3D). From the curve, approximately 2,000 Dscam1 isoforms are sufficient to maintain a normal overlap between class I and III dendrites (Fig 3D). Therefore, we conclude that isoform diversity is required for dendrite self/non-self discrimination in an exon cluster- or isoform-independent manner.

### Reducing Dscam1 diversity causes MB phenotypic defects in an exon cluster–specific manner

**Fig 4:**
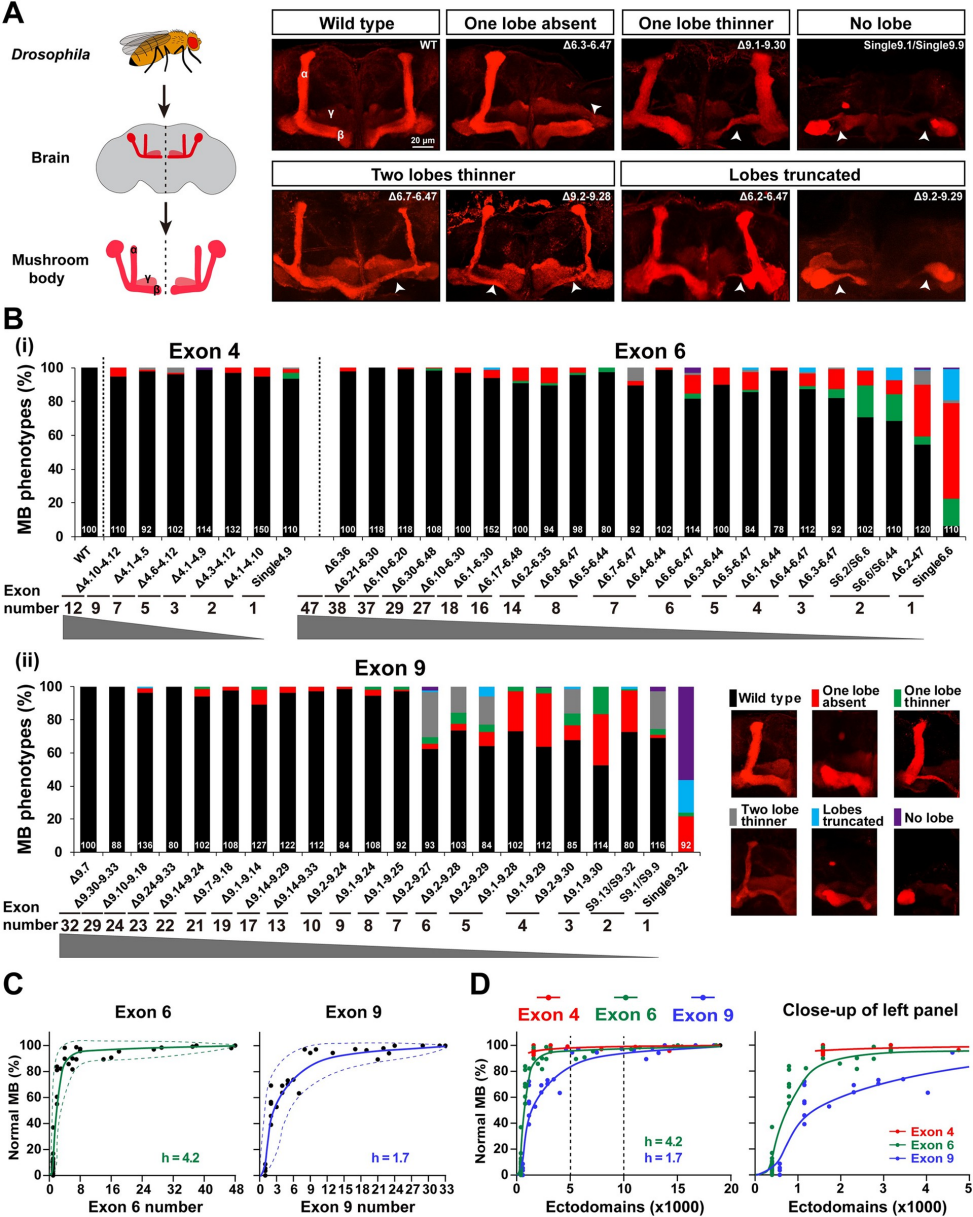
Reduction of Dscam1 diversity affects MB morphology. **See also S5 and S6 Figs. (A)** MB lobe morphology in wild-type and *Dscam1* mutant animals was visualized with monoclonal antibody 1D4 (anti-FasII, red). Scale bar, 20 μm. **(B)** Quantification of the MB phenotypes in mutants with a deletion of variable exons 4, 6, and 9. The numbers at the bottom represent the number of MBs (i.e., brain hemispheres) examined for each genotype. **(C)** The normal MB phenotype rate largely correlated with the numbers of variable exon 6 (left panel) and exon 9 (right panel). The dashed curves show the spectra of phenotypic variation. **(D)** A comparison of the phenotype–diversity correlates among variable exon 4, 6, and 9 clusters. A close-up of the left panel is shown on the right. These data indicate that reducing Dscam1 diversity affects MB phenotype in an exon cluster–specific manner. Data used to generate graphs can be found in S1 Data. Dscam1, Down syndrome cell adhesion molecule 1; MB, mushroom body; WT, wild type.

Next, we assessed the extent of Dscam1 isoform diversity necessary for MB development. Previous studies showed that 1,152 to 4,752 isoforms were required to form a normal bi-lobed MB structure [24]. Recent studies have revealed a 0% to 8% frequency of MB phenotype defects in *Dscam*^Single4.x^ mutants with a single exon 4 variant, while *Dscam*^Single6.x^ mutants encoding 396 isoforms exhibited severe MB defects [22]. As expected, the frequency of defective MB phenotypes decreased as the potential number of available exons 4, 6, and 9 increased (Fig 4A–4C). In addition, the MB phenotypes of the *Dscam*^Δ4.x-4.x’^/*Dscam*^+^, *Dscam*^Δ6.y-6.y’^/*Dscam*^+^, and *Dscam*^Δ9.z-9.z’^/*Dscam*^+^ heterozygous mutants were indistinguishable from the wild-type controls (S5C Fig). Moreover, the type of MB phenotypic defects was significantly altered. Among them, the most common phenotypic defects included absence, truncation, and thinning of one lobe (Fig 4A and 4B). Lobe truncation and thinning of 2 lobes accounted for up to approximately 76% of defects in some mutants, such as *Dscam*^Δ9.2–9.27^ (Fig 4B). Taken together, these data show that the normal MB phenotype correlates to a large extent with the number of variable exons. The correction curve fitted a sigmoidal correlation with different Hill values, indicating that MB development is differentially sensitive to reduced diversity in exons 4, 6 or 9 (Figs 4C and S5A).

Reducing exon 4s, 6s, and 9s had different effects on the MB phenotype (Fig 4D). For example, *Dscam*^Single4.x^ mutants encoding 1,584 isoforms exhibited 0% to 8% frequency of MB phenotype defects, while *Dscam*^Δ6.1–6.44^ and *Dscam*^Δ6.4–6.47^ mutants encoding the same degree of isoforms exhibited 2% and 13% frequency of MB phenotype defects, respectively (Figs 4B and S5B). We speculate that MB defects are more sensitive to reductions in exon 6 cluster than in exon 4 cluster. Remarkably, reducing exon 9s had a much greater effect on the MB phenotype than reducing exon 4 or 6 clusters, as evidenced by the lowest Hill value for exon 9 (4.2 versus 1.7) (Fig 4D). This indicates that MB phenotypes are more sensitive to the reduction of exon 9 cluster than exon 4 and 6 clusters. In particular, the reduction to 4,032 isoforms in *Dscam*^Δ9.2–9.27^ mutant and 10,944 isoforms in *Dscam*^Δ9.1–9.14^ mutant still exhibited over 38% and 11% of MB defects, respectively (Fig 4B). Mosaic analysis with a repressible cell marker (MARCM) at the single-cell resolution showed that some mutant animals with deletions encoding >10,000 isoforms (i.e., *Dscam*^Δ6.21–6.30^; *Dscam*^Δ6.10–6.30^; and *Dscam*^Δ9.24–9.33^) exhibited 20% to 36% axonal defects (S6 Fig). These data suggest that up to 10,000 Dscam1 isoforms, at least for some subsets of isoforms, are required for normal MB axon patterning.

Although the normal MB phenotypes in mutant flies are positively correlated with the number of available exons, these mutants with the same degree of diversity exhibit considerable phenotypic variation in exon clusters 4, 6, or 9 (Fig 4B). We observed a greater spectrum of MB phenotype variations in the exon 9 cluster than in exons 6 and 4 (Fig 4D), suggesting that exon 9 isoforms mediate MB development with higher specificity than exon 4 and exon 6 isoforms.

### The influence of Dscam1 diversity on axonal branching of mechanosensory neurons in an exon cluster–related manner

**Fig 5:**
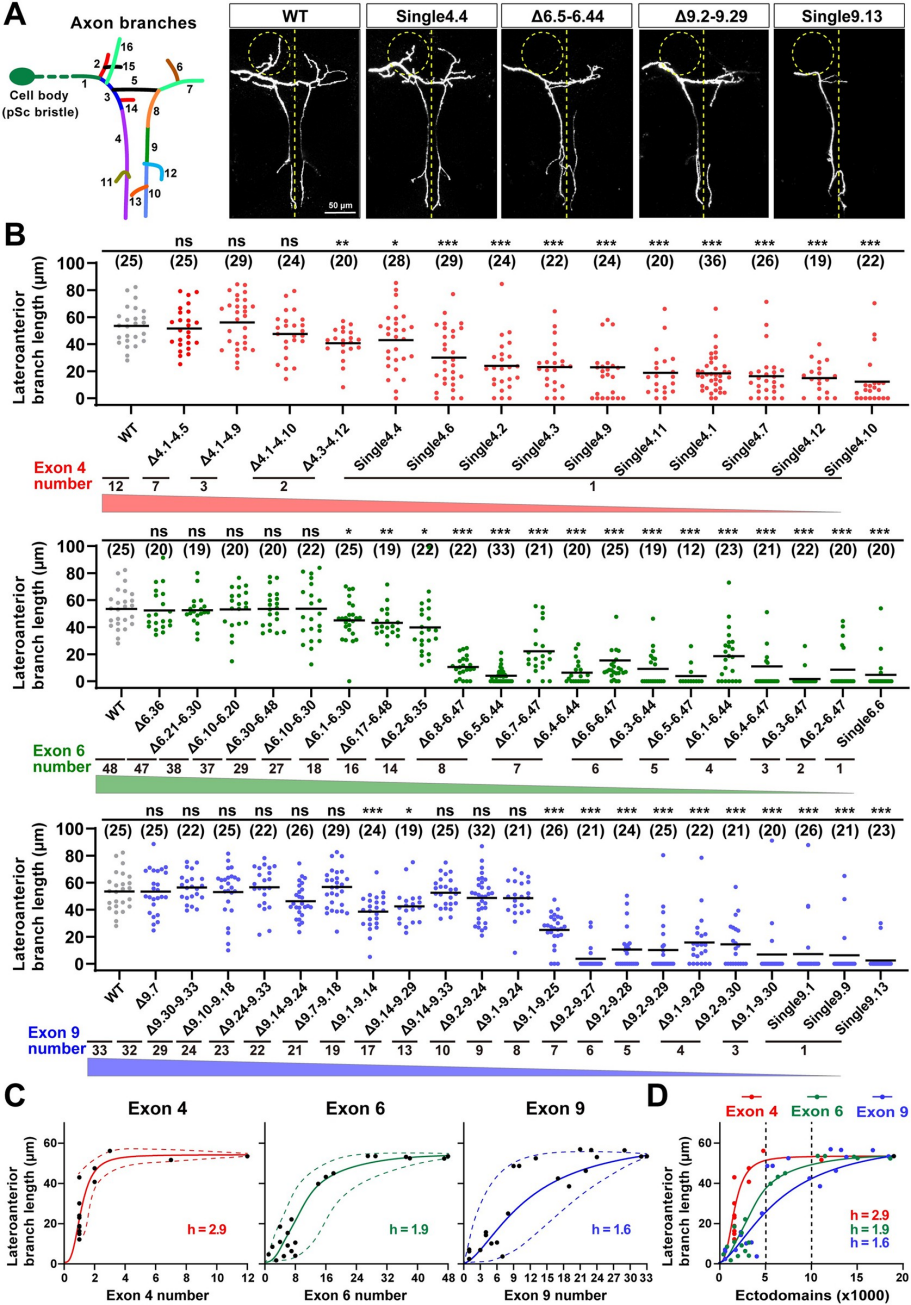
The reduction of Dscam1 diversity affects axonal branching in MS neurons. **See also S7–S9 Figs. (A)** Schematic of the axon trajectory of a single MS neuron within the VNC. Branch segments were assigned different colors for scoring. Representative dye tracing images of the wild-type and *Dscam1* mutant flies. Dashed circles indicate the location of the lateroanterior branch. ns, not significant; *p < 0.05; **p < 0.01; ***p < 0.001 (Student *t* test, two-tailed). Scale bar, 50 μm. **(B)** Quantitative analysis of the length of lateroanterior branch in wild-type and *Dscam1* mutant flies. Numbers in parentheses represent the number of pSc neurons observed in each genotype. **(C)** The average length of lateroanterior branch positively correlated with the number of variable exons 4, 6, and 9. The dashed curves show the spectra of phenotypic variation among different variants. **(D)** A comparison of phenotype–diversity correlates among variable exon 4, 6, and 9 clusters. Reducing Dscam1 diversity affected MS axonal branching in an exon cluster–specific manner. Data used to generate graphs can be found in S1 Data. Dscam1, Down syndrome cell adhesion molecule 1; MS, mechanosensory; VNC, ventral nerve cord; WT, wild type.

Previous studies have shown that the *Dscam1* mutant allele with a single exon 6 or 9 variant exhibits severe disruption of the collateral formation of MS axons [18,24]. We analyzed the axonal branching of adult *Dscam1* mutants with reduced numbers of exons 4, 6, or 9 to explore further the role of Dscam1 diversity in the axonal patterning of MS neurons. The *Dscam*^Single4.x^ mutant allele lacked all but one of the exon 4 variants and exhibited mild to moderate MS axonal branching defects (Figs 5B and S9B). This axonal branching improves with increasing numbers of exon 4s, including the length of the lateroanterior branches, total number of branches, and branching patterns of MS axons (Figs 5B, S7A, S7B, and S9). Similarly, MS axonal phenotypes improve with increasing numbers of exons 6 or 9 (Figs 5A, 5B, S7A, S7B, and S9B). These data indicate that Dscam1 diversity largely correlates with MS axonal branching (Figs 5C and S7C). These nonlinear relationships fit a sigmoidal correlation with different Hill values (Fig 5C).

Reducing exon 4, 6, and 9 diversities resulted in considerable differences in MS axonal phenotypes; exon 4 had the least effect, and exon 9 had the largest effect, as evidenced by the Hill values (Fig 5D). These phenotype–diversity correlation comparisons indicate that MS axonal branching is much more sensitive to a reduction of exon 6 or exon 9 diversity than to the reduction of exon 4 diversity. For example, *Dscam*^Δ9.2–9.27^ mutant encoding 4,032 isoforms exhibit only 3.9 μm lateroanterior branch lengths of the MS axon (Fig 5B). A reduction to 10,944 isoforms in *Dscam*^Δ9.1–9.14^ flies still exhibited a lateroanterior branch length of 38.5 μm, much shorter than the wild-type average of 53.5 μm (Fig 5B). Similar results were observed in *Dscam*^Δ6.y-6.y’^ flies. Inconsistent with previous studies of a few mutants reducing exon 4s [24], these data suggest that thousands of Dscam1 isoforms may not be sufficient for normal MS axonal branching, at least in some subsets. In contrast, up to 10,000 isoforms may be required for normal axon development in MS neurons. These data imply that MS axonal growth and branching may be associated with intrinsic properties of variable domains.

Previous genetic analyses of the *Dscam*^Single6.y^ or *Dscam*^Single9.z^ mutants showed no significant differences in MS axonal defects between them, so it is speculated that the process does not require specific isoforms [18,24]. Consistent with this, *Dscam*^Single9.z^ mutants exhibit similar penetrance of MS axon defects (Fig 5B). However, the differences between these mutants may be overridden due to excessive defects in the *Dscam*^Single6.y^ or *Dscam*^Single9.z^ mutants. To address this possibility, we performed pairwise comparisons of MS axonal defects among *Dscam*^Single4.x^ mutants encoding 1,584 potential ectodomain isoforms that exhibit mild to moderate axonal branching defects. Some *Dscam*^Single4.x^ mutants exhibited significant differences in the lateroanterior branch length and total branch number of MS axons (Figs 5B, S8A, and S8B). For example, the lateroanterior branch length of the MS axon ranged from a maximum of 40.0 μm in *Dscam*^Single4.4^ to a minimum of 12.0 μm in *Dscam*^Single4.10^ (Figs 5B and S8A). Similar results were observed for the total branch number of MS axons (S5B and S8B Figs). Moreover, *Dscam*^Δ6.7–6.47^ and *Dscam*^Δ6.4–6.44^ mutants encoding the same degree of isoform diversity showed significant differences in MS axon defects (Figs 5B, S8C and S8D). *Dscam*^Δ6.y-6.y’^ and *Dscam*^Δ9.z-9.z’^ flies with more remaining exons had more penetrance of MS axon defects (Figs 5B, S8C and S8D). These data indicate that Dscam1 diversity was differentially required for normal axonal growth and branching in an exon cluster- and isoform-related manner.

### Reducing Dscam1 levels significantly affects phenotype–diversity correlations

**Fig 6:**
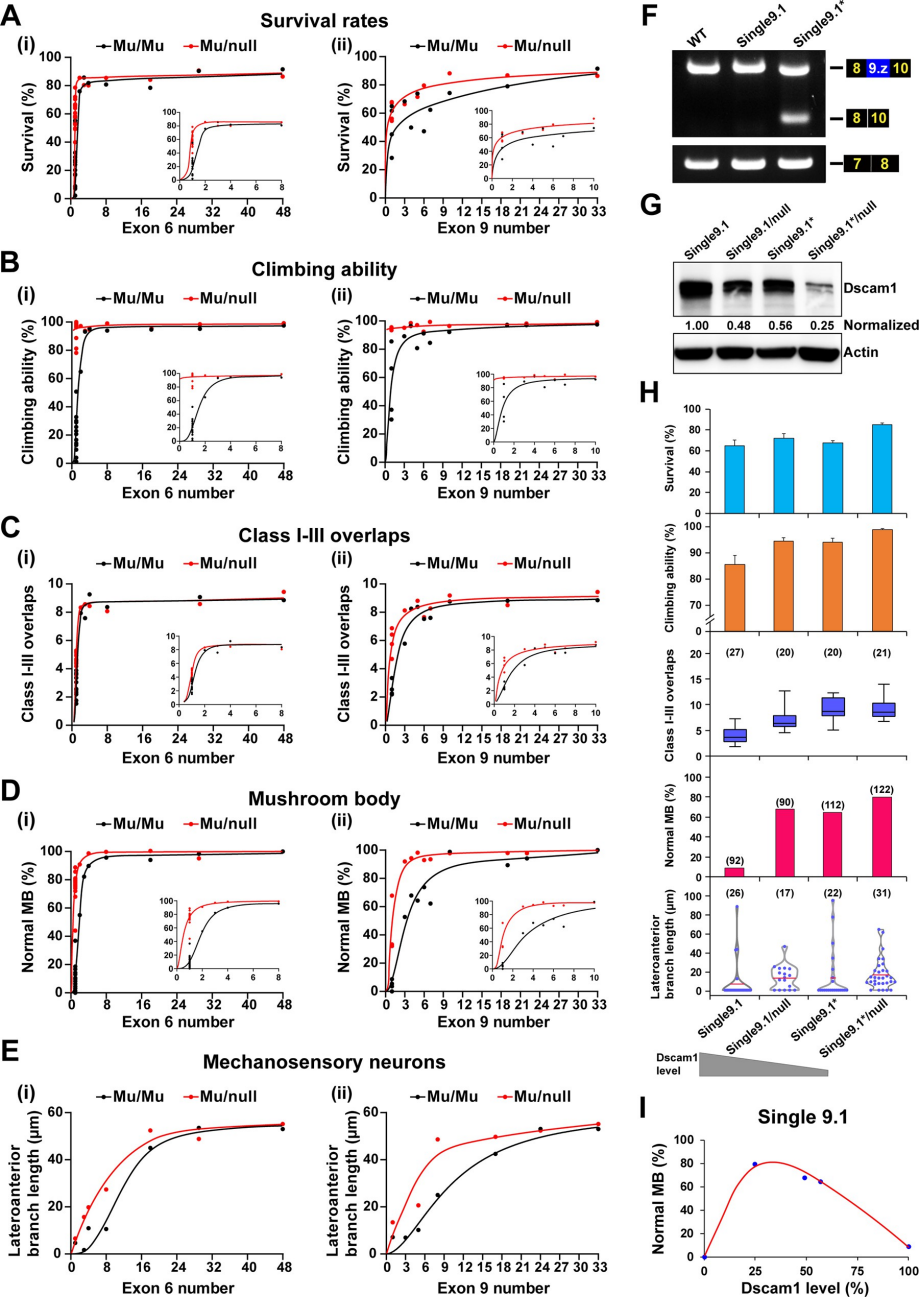
Effect of reducing Dscam1 expression level on the phenotype–diversity correlations. **See also S10 and S11 Figs. (A-E)** A comparison of Dscam1 phenotype–diversity correlates between Mu/Mu (homozygous mutants, black lines) and Mu/null (mutant/null, red lines) mutants for survival rates (A), climbing ability (B), class I-III overlaps (C), MB (D), and MS neurons (E) phenotypes. Reducing Dscam1 expression levels could partially rescue defects caused by reduced diversity in *Dscam*^Δ6.y-6.y’^ and *Dscam*^Δ9.z-9.z’^ mutant flies. **(F)** RT-PCR was performed from the head tissues of wild-type, *Dscam*^Single9.1^, and *Dscam*^Single9.1^* mutant flies. The primers anneal to exons 8 and 10 or exons 7 and 8. **(G)** Western blot analysis. Dscam1 levels were normalized to β-actin, and the expression levels were compared to *Dscam*^Single9.1^, which was set to 1. **(H)** The normal phenotypes in mutants expressing a single exon 9.1 improved with the decrease in the Dscam1 expression level. **(I)** The relationship between the frequency of normal MBs versus the corresponding Dscam1 protein levels in the *Dscam*^Single9.1^ mutants. Data used to generate graphs can be found in S1 Data. Dscam1, Down syndrome cell adhesion molecule 1; MB, mushroom body; MS, mechanosensory; RT-PCR, reverse transcription PCR; WT, wild type.

We compared *Dscam*^Δ6.y-6.y’^/*Dscam*^null^ and *Dscam*^Δ9.z-9.z’^/*Dscam*^null^ flies bearing one copy of a mutant allele, which reduced the Dscam1 level by approximately 50% of the wild type to analyze further the effect of reduced Dscam1 levels on phenotype–diversity correlations (S10A Fig). When a copy of the mutant Dscam1 was removed, there was a reduced downward trend in fly viability with the reduction of the remaining exon 6s or exon 9s (Figs 6A and S10B). These data suggest that reducing the Dscam1 protein level restores fly viability due to reduced diversity. Similar trends were observed in fly locomotion (Figs 6B and S10C). When we removed 1 copy of the mutant *Dscam1*, these phenotype–diversity correlations changed considerably in the overlaps between dendrites of class I and III neurons and the phenotypes of MB and MS neurons (Figs 6C–6E and S11). These data indicate that reducing Dscam1 levels to half can partially rescue the defects caused by reduced diversity.

We constructed mutants expressing a single exon 9.1 with varying degrees of Dscam1 levels by changing intronic elements (designated *Dscam*^Single9.1^*), to explore how reduced Dscam1 levels and isoform diversity jointly affect fly phenotypes. RT-PCR analyses showed that *Dscam*^Single9.1^* mutants exhibited considerable exon 9 skipping (Fig 6F). Western blot analyses indicated that Dscam1 expression levels in *Dscam*^Single9.1^* were reduced to approximately 60% of the wild type (Fig 6G). Phenotypic spectra in *Dscam*^Single9.1^* were indistinguishable from those in *Dscam*^Single9.1^/*Dscam*^null^ flies, which is consistent with comparable levels of Dscam1. When we removed 1 copy of the *Dscam*^Single9.1^* mutant, the resulting *Dscam*^Single9.1^*/*Dscam*^null^ mutant, whose Dscam1 expression level was reduced to approximately 30% of the wild type, increased to 80% of the normal MB phenotype (Fig 6H). Similarly, almost all other phenotypes were remarkably improved in *Dscam*^Single9.1^*/*Dscam*^null^ flies compared to *Dscam*^Single9.1^* homozygous flies. Overall, the various phenotypes of mutants expressing a single exon 9.1 improved with decreasing levels of Dscam1 expression (Fig 6H). However, considering that Dscam1 null mutant and knockdown by RNAi are adult homozygous lethal [16,34], the normal phenotypes were expected to peak at a certain threshold and then decline as Dscam1 expression levels were further reduced from approximately 25% to null (Fig 6I). These results suggest a profound relationship between Dscam1 expression levels and isoform diversity.

## Discussion

This study reveals the differential requirement for Dscam1 diversity in diverse neurons via phenotype–diversity correlation analysis. We show that reducing exon clusters 4, 6, or 9 has a comparable effect on da dendrite self/non-self discrimination but causes differential defects in MB and MS neurons, with exon 9s having the largest effect. We conclude that the role of Dscam1 diversity in dendrite self/non-self discrimination is nonspecifically mediated by its isoform diversity, whereas a separate role essential for other nerve developments, probably in axonal growth and branching, requires variable domain- and isoform-related functions. Our findings highlight that fly Dscam1 isoforms, like mammalian protocadherins (Pcdhs), play redundant and differential roles in neuronal circuits.

### How many Dscam1 isoforms are required in vivo?

Thousands of Dscam1 isoforms are necessary to provide neurons with a robust discrimination mechanism to distinguish between self and non-self neurites [24]. Of the 3 classes of neurons analyzed previously, flies with 1,152 potential isoforms retained substantial branching defects, but those with 4,752 isoforms were indistinguishable from the wild-type controls. Therefore, thousands of isoforms are required for the normal patterning across 3 classes of neurons: dendrite patterning in da neurons and axonal branching in MB and MS neurons (S12D–S12F Fig). Genetic analysis of a more comprehensive repertoire of fly mutants in our present study does not support the conclusion of the latter 2 neuronal systems (S12A–S12C Fig). This discrepancy is due to the relatively small number of deletion mutants analyzed in their study and the lack of representative mutants. In particular, the crucial deletion mutants with Dscam1 diversity ranging from 1,500 to 15,000 isoforms with partial deletion of exon 6 or exon 9 clusters are lacking in the previous study [24].

The present study shows that different numbers of isoforms are required for normal patterning in the 3 neuronal systems. Of the three, normal da dendrite self/non-self discrimination requires a minimum number of Dscam1 isoforms; approximately 2,000 isoforms are sufficient for normal dendrite patterning, which is largely independent of variable exon clusters. In contrast, some flies with >10,000 potential isoforms exhibit substantial MB and MS defects. (Figs 4B, 5B and S6). These data imply that normal axon patterning in MB and MS neurons may require up to 10,000 isoforms, which are likely to be associated with specific variable exon clusters or isoforms. These data suggest that many or possibly even the entire repertoire of diverse Dscam1 isoforms are necessary for normal development in some neurons. We show that previous reports [24] have overestimated the number of isoforms required for normal dendrite self/non-self discrimination but have underestimated the number of isoforms required for normal axon patterning in MB and MS neurons.

### Functional redundancy and specificity of Dscam1 isoforms

Genetic studies have shown that Dscam1 diversity is functionally required in neuronal wiring [11–14,17–19]. However, it has been challenging to obtain reliable evidence that the large diversity of Dscam1 isoforms is utilized to generate specificity. Expression studies have shown that exons 4 and 6 are alternatively spliced largely randomly in single neurons [7,10,35]. Genetic studies have demonstrated that mutant animals with deletions encoding the 4,752 and 14,256 isoforms are indistinguishable from the wild-type controls in neuronal branching phenotypes [17,24]. Functionally, mutants with a single isoform or a single variable exon exhibited no obvious phenotypic discrepancy [13,18,24], and overexpression of diverse isoforms rescued the Dscam1 phenotypes of neurons equally well [11,14,23]. These results posit that alternative exon variants may be functionally redundant during neuronal development and that specific Dscam1 isoforms may not be functionally important.

**Fig 7:**
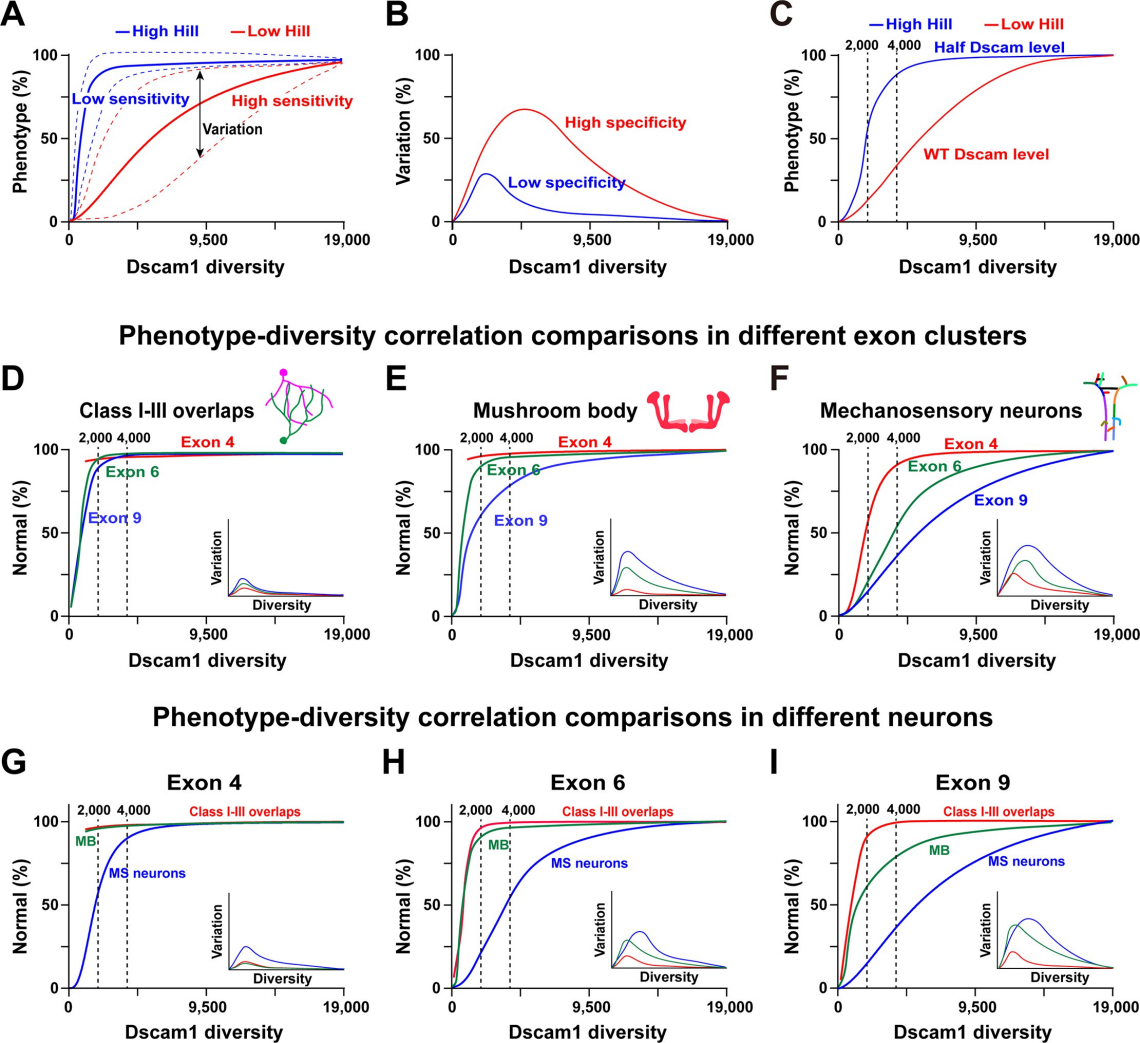
Phenotype–diversity correlations reveal differential requirements of Dscam1 diversity in diverse neurons. **See also S12 Fig. (A)** The normal phenotype is a sigmoidal function of Dscam1 diversity with various Hill coefficients. The Hill coefficient (h) is the sensitivity parameter. The dotted lines represent the spectra of phenotypic variation. **(B)** The spectra of phenotypic variation as a function of Dscam1 diversity and an indicator to assess isoform specificity. The larger variation spectra reflect higher isoform specificity. **(C)** Reducing the Dscam1 level affected the phenotype–diversity correlations considerably, as indicated by the higher Hill value. (**D-F**) A comparison of the phenotype–diversity correlations in 3 exon clusters. Exon 4, 6, or 9 clusters exhibited similar phenotype–diversity correlation curves in class I-III overlaps (D). The small spectra of variation suggest that this process was largely independent of the isoform identity. However, exon clusters 4, 6, or 9 exhibited considerable differences in the correlation curves in MB (E) and MS neurons (F), suggesting an association with a specific exon cluster. Moreover, the large spectra of variation are associated with the isoform identity. **(G-I)** Phenotype–diversity correlation comparisons of different neurons in individual exon 4 (G), 6 (H), and 9 (I) clusters. These data revealed the differential requirement of Dscam1 diversity in diverse neurons. Dscam1, Down syndrome cell adhesion molecule 1; MB, mushroom body; MS, mechanosensory.

However, our genetic analysis supports a potential association with specific Dscam1 isoforms or variable domains in the wiring of at least some neurons. Combining the deletion experiments and associated neural phenotypic data from this study with other laboratories [13,16,17,22,24], we propose a general model depicting the functional redundancy and specificity of Dscam1 isoforms. The mathematical modeling suggests that the phenotypic spectra are a sigmoidal function of Dscam1 diversity, albeit with varying Hill values (Fig 7A). The Hill value represents the sensitivity of the phenotype to Dscam1 diversity. In contrast, the spectra of variation between different variants or subsets may represent their functional differences (specificity) (Fig 7B). In addition, these phenotype–diversity correlations were influenced by overall Dscam1 levels (Fig 7C). The phenotypes of 3 classes of neurons improved with increasing potential numbers of isoforms, albeit with different correlation curves. One concerned da dendrite self/non-self discrimination (Fig 7D), where exon clusters 4, 6, or 9 exhibited similar phenotype–diversity correlation curves (similar Hill values), largely independent of the exon cluster. Moreover, the spectra of variation were very small, indicating that self/non-self discrimination is largely independent of the identity of the isoforms. In this case, individual exon clusters or isoforms tend to be of equal functional importance. Therefore, our correlation modeling is consistent with previous findings that dendrite self/non-self discrimination is nonspecifically mediated by Dscam1 diversity [24].

In contrast, exon clusters 4, 6, or 9 exhibited considerably different correlation curves (different Hill values) in MB and MS neuron axons (Fig 7E and 7F), demonstrating that this correlates with specific exon clusters. Furthermore, a large range of variants correlates with the identity of the isoforms. For example, since exon cluster 9 has the largest spectra of variation among different variants, we infer that exon 9 isoforms have the highest specificity. This idea is supported by genetic evidence that altering the variant composition by deleting docking sites in exon 4 or 6 clusters leads to <5% subtle to mild MB defects [27,36]. However, deleting upstream docking sites in the exon 9 cluster causes up to 60% of MB defects [28]. Diverse neurons exhibit considerable correlation curve differences in individual exon clusters (Fig 7G–7I). In this scenario, we conclude that the separate role played by Dscam1 isoforms in some nervous development requires variable domain- or isoform-related functions.

These results suggest that Dscam1 isoforms from exon 4, 6, or 9 clusters may be functionally redundant and specific. We propose that Dscam1 isoforms have 2 functions. First is the role shared by the Dscam1 isoforms in exon 4, 6 and 9 clusters or isoforms, which provides each neuron with a unique identity to distinguish self from non-self neurites. This process does not require a specific cluster or isoform. Second is a separate role essential to other neurodevelopmental contexts, possibly in axonal growth and branching, which requires isoform-related functions. These genetic data reveal redundant and specific roles for Dscam1 individual isoforms in neuronal wiring.

### How is the specificity of Dscam1 isoforms acquired?

It remains to be seen how Dscam1 isoforms acquire their specificity. Mechanistically, we speculate that the exon cluster and exon variant specificity of Dscam1 isoforms may be attributable to differences in expression levels and intrinsic features of variable domains or individual isoforms. Biochemical analyses indicate that homophilic binding varies remarkably among individual variable domains or isoforms (i.e., 19–58 for variable Ig2, 13–37 for variable Ig3, and 1–38 for variable Ig7) [5]. In addition, this specificity may be related to their distinct expression patterns, in which the exon 9 cluster exhibits the highest bias in expression bias [35]. Dscam is associated with extracellular ligands or coreceptors, like Netrin, Slit, and Ptp69D [37–40]. It is conceivable that the binding affinity of Dscam1 to these ligands and coreceptors may vary, consequently affecting downstream signaling events. It will be interesting to test the alleles for specific interactions with downstream effectors such as Abl or Pak [41,42], as upstream splicing events in *Dscam1* can influence downstream events. These scenarios align with our observation that reducing isoform diversity led to developmental and neuronal defects in an exon cluster–and exon variant–related manner. Therefore, we speculate that combinatorial mechanisms contribute to the specificity of Dscam1 isoforms through homophilic or heterophilic binding.

### Functional parallels between fly Dscams and mammalian Pcdhs

The fly *Dscam1* gene closely resembles the mammalian *Pcdh* gene cluster: Both have a complex genomic locus comprising 3 repeat arrays and encode many distinct isoforms that exhibit stochastic expression and isoform-specific homophilic binding [9]. These parallels between fly Dscams and mammalian Pcdhs suggest that both may provide neuronal identity for self-avoidance and self/non-self discrimination [3,9,43–45]. These parallels are strengthened by our recent discovery of a “hybrid” *sDscam* gene family in Chelicerata, which has a high sequence similarity to fly *Dscam1* but shares an organizational resemblance to the 5′ variable region of mammalian-clustered *Pcdhs* [46]. Like the fly Dscam1 and mammalian-clustered Pcdhs, Chelicerata sDscams exhibit isoform-specific homophilic binding [47,48].

There are fundamental differences between these 2 gene families in the observations: Individual *Dscam1* exon clusters or isoforms appear to be functionally equivalent [13,24], whereas certain *Pcdh* gene clusters or isoforms have specific roles in the nervous system [49–51]. For example, the C-type Pcdh isoforms have unique roles in neuronal survival [49,50], while Pcdhac2 is required for axonal tiling and assembly of serotonergic circuitries in mice [51]. Moreover, destroying the *Pcdhα* or *Pcdhγ* genes caused various phenotypic defects, suggesting that individual *Pcdh* gene clusters may have distinct or complementary roles in different neuronal cell types [3]. Our findings suggest that, like mammalian Pcdhs, individual exon clusters or certain isoforms of fly Dscam1 have redundant and specific roles in neuronal circuits. Individual exon 4, 6, and 9 clusters are consistently under different evolutionary pressures and display distinct expression patterns [10,35]. Although fly Dscam1 and mammalian Pcdh proteins do not share any sequence homology, they appear to underlie mechanistic and functionally analogous strategies. It remains to be seen whether a subset of Dscam1 isoforms acts synergistically. Exon 4 diversity appears less dispensable for MB development but may play a role in other, as yet unidentified, developmental contexts. Further functional studies explicitly targeting these variable exon 4, 6, and 9 clusters are required to address these possibilities.

## Materials and methods

### Materials: Fly stocks

We used the *W*^*1118*^ line as the wild-type control. The CRISPR/Cas9 system was used in *{nos-Cas9}attP2* flies to generate Dscam1 mutations [52]. *if/CyO* or *if/CyO*.*GFP* lines were used as the balancer stocks for mutants. The GFP expression in class I da neuron was driven by the *Gal4*^*221*^ line, as previously described [33]. *FRT42D* and *hsFLP*,*UAS-mCD8-GFP;FRT42D*,*tubP-Gal80/CyO;Gal4-OK107* lines were used to MARCM clones of MB neurons. Unless exceptional conditions were mentioned, all lines were cultured on a standard cornmeal medium at 25°C.

### Generation of Dscam1 mutant flies

The CRISPR/Cas9 system combined with nonhomologous end joining generated the variable exon deletion in *Dscam1* [52]. A detailed schematic for knocking out the variable exon of *Dscam1* using the CRISPR/Cas9 technology is shown in S1 Fig. Two sgRNAs were coinjected into *{nos-Cas9}attP2* embryos performed by UniHuaii, China. The mosaic fly was crossed with the balancer stock. Then, the female offspring were collected and detected by PCR analysis. Next, the male offspring from the mutation tubes were recrossed to the balancer stock. Finally, genomes extracted from each male fly were identified by PCR analysis to acquire the final mutation lines. All mutant lines were backcrossed with *W*^*1118*^ lines 5 times to avoid potential off-target effects. If 2 crosses during the mutant screening process using a balancer (wild-type) and additional 2 after the 5 crosses with *W*^*1118*^ were considered, the total number of crosses with the wild-type was 9. The primers used for sgRNA and mutant screening are listed in S1 Table. The mutation sequences of all *Dscam1* variable exon deletion mutants are listed in S2 Table.

### RT-PCR detection

The total RNA from head tissues was obtained using Trizol reagent (Invitrogen, Waltham, MA, USA). Reverse transcription (RT) was performed following the SuperScript III system (Invitrogen) using specific primers for the *Dscam1* constitutive exon 10. The cDNA was amplified by PrimeSTAR DNA Polymerase (TaKaRa, Kyoto, Japan) using specific primers in each variable exon cluster of *Dscam1*. The final products were detected by electrophoresis with 1.5% agarose gel and photographed using the GIS 1D Gel Image System (ver. 3.73; Tanon, Shanghai, China). The specific primers used for RT-PCR are listed in S1 Table.

### Western blot analyses

We used a strong RIPA lysis buffer (CW-BIO, Jiangsu, China) and the PMSF (Phenylmethylsulfonyl fluoride) protease inhibitor (Beyotime, Shanghai, China) to obtain protein samples from the head tissues of mutant flies. Western blot was performed according to standard western blot protocol (Abcam, Cambridge, UK). We used primary antibodies to Dscam1 (ab43847, diluted 1:5,000), β-actin (ab8227, diluted 1:10,000), and secondary antibodies (goat anti-rabbit IgG, 1:10,000, CW-BIO) in this process. The immunoreactive bands of Dscam1 and β-actin protein were detected using the eECL Western Blot Kit (CW-BIO) and Tanon 5200. Semiquantitative western blot analysis compared the Dscam1 protein levels from the head tissues of different genotypes. Image J software (NIH, Maryland, USA) was used to analyze quantitatively the western blot bands.

### Survival rate detection

Survival rates include 3 development processes. For the hatching rate, approximately 200 embryos were collected, and the hatched embryos were counted after incubation for 48 h at 25°C. We collected 90 larvae from the hatched embryos (second instar larvae) and distributed them into 3 new food tubes. After 3 to 4 days, the pupae were counted, and after another 4 to 5 days, the adult flies in the food tubes were counted. Three biological replicates were performed for each development stage experiment.

### Climbing ability detection

Approximately 30 two-day-old male flies were collected into tubes. A line was drawn at 2 cm around each tube. All the flies were gathered at the bottom after tapping the tubes. After 10 s, the flies crossing the 2-cm line were counted. This was repeated 10 times. The average percentage of flies that crossed the line indicated the climbing ability of the flies. Three biological replicates were performed.

### Imaging of da neurons

As previously described, the class I da neurons were GFP-labelled using the *Gal4*^*221*^ line [33]. Larvae with GFP-labelled class I da neurons were dissected to detect the overlaps between class I and III da dendrites. After dissection, the larval epidermis was fixed for 25 min at room temperature in 4% paraformaldehyde and blocked in 5% bovine serum albumin (BSA) (diluted in PBST, phosphate-buffered saline (PBS) containing 0.1% Triton X-100) after 3 washes in PBST (20 min each time). Next, horseradish peroxidase (HRP) antibody (Cy3-conjugated Affinipure Goat Anti-Horseradish Peroxidase, diluted 1:200) was used to incubate the larval epidermis at 4°C overnight. After 3 washes in PBST for 20 min each, the larval epidermis was mounted in ProLong gold antifade reagent (Invitrogen). Immunofluorescence staining was imaged by an LSM800 confocal microscope (Carl Zeiss, Oberkochen, Germany). Both 488 and 594 fluorescent channels were used during the imaging process. Approximately 20 images per line were obtained for analysis. The confocal images were processed using ZEN 2.1 software. The green single-channel exported images were used for the subsequent analysis of class I self-crossing, and the dual-channel exported images were used for the subsequent analysis of class I-III overlap.

### MB neurons immunostaining

MB neuron immunostaining was performed as previously described [14]. Specifically, 3- to 5-day-old adult flies were dissected in PBS, and the brains were fixed in 4% paraformaldehyde at room temperature for 35 min. After 3 washes in PBST for 20 min each time, the brains were blocked by 5% BSA for 1 h at room temperature. After 3 standard washes, the brains were incubated for 48 h at 4°C with the primary antibodies (anti-FasII, DSHB, diluted 1:2 in PBST). Following another 3 standard washes, the brains were incubated at room temperature for 4 h with the secondary antibodies (Alexa594-goat-anti-mouse IgG, Earthox, diluted 1:400 in PBST). Next, the brains were mounted in ProLong gold antifade reagent (Invitrogen) after another 3 standard washes. Finally, the MB immunofluorescence staining was imaged with an LSM800 laser scanning confocal microscope (Carl Zeiss). A single 594 fluorescent channel was used during the imaging process. Approximately 50 images per line (100 hemispherical MBs) were obtained to detect phenotypic defects. The MB confocal images were processed using ZEN 2.1 software.

### MS neurons carbocyanine dye labeling

Dye labeling of MS axon terminals in the ventral nerve cord (VNC) was performed as previously described [21]. Briefly, the DiD (20 mg/ml in ethanol) or DiI (20 mg/ml in 1:1 dimethyl formamide/ethanol) dyes were injected into the sockets of MS neurons. After 48- to 72-h incubation at room temperature, the dye-filled VNC was imaged on a Leica TCS SP8 or a Zeiss LSM 880 confocal microscope. Approximately 20 images per line were obtained for analysis. Confocal images were processed using Image J and PhotoShop software. Image J was used to trace the length of lateroanterior branches of MS neurons in each image. The total number of branches and branching patterns of the MS axons in each image was calculated as previously described [24].

### MARCM clones

The MARCM technique generated single-cell clones of MB neurons [53]. The *Dscam1* mutant flies were crossed to the FRT42D lines to obtain mutant genes by linking the FRT sites through natural chromosome recombination. Next, the mutant lines with the FRT sites were crossed to *hsFLP*,*UAS-mCD8-GFP;FRT42D*,*tubP-Gal80/CyO;Gal4-OK107* line. The pupation stage offspring were subjected to heat shock in a 37°C water bath for 1 h. Next, the adult lines were dissected, and the MBs were immunostained as described above. Finally, the single-cell clone and immunostained MBs were imaged with an LSM800 laser scanning confocal microscope (Carl Zeiss). Both 488 and 594 fluorescent channels were used during the imaging process. Confocal images were processed using ZEN 2.1 software.

### Statistical analysis

The data of the *Dscam*^Single^ (10 single variable exon 4, 15 single variable exon 6, and 4 single variable exon 9) mutants relating to fly survival, locomotion, class I-III overlap, and MB defect phenotypes are from our previous study [22]. All the raw data used to generate the graphs are located in S1 Data. Three biological replicates were performed for the fly viability and locomotion detection, and error bars represent the means ± SD. In da neurons, self-avoidance or self/non-self discrimination was assessed by counting the number of class I self-dendritic branch crossings or the number of class I and class III dendrite overlaps in each image. Significant differences in the number of overlapping points of class I self-branches, or class I-III dendrites between the wild-type and mutants were verified by the Student *t* test. The proportion of defect images for each mutant were calculated based on the MB neuron morphological images, including the proportion of each defect type. In MS neurons, significant differences in the lengths of lateroanterior branches and the total number of branches of MS neurons between the wild-type and mutants were verified by the Student *t* test. We performed two-tailed Student *t* tests for phenotype analyses without using blind method, with *p* > 0.05 considered nonsignificant (n.s.), *p < 0.05, **p < 0.01, and ***p < 0.001.

### Correlation analysis and curve fitting

To assess the effects of Dscam1 diversity on different phenotypes, we employed curve fitting, using the number of variable exons or the number of Dscam1 ectodomains as independent variables and the phenotype defect as the dependent variable. The correlation analysis between phenotype defects and Dscam1 diversity was conducted using GraphPad Prism version 8.0.2 for Windows (GraphPad Software, San Diego, California, USA). Considering that the Hill equation can be flexibly fitted to the data of different Hill coefficients, which are suitable to interpret the difference in 3 neurons, or in exon 4, 6, and 9 clusters, the Hill equation was chosen to curve fitting. Fit quality was evaluated based on the R-squared value. After fitting the curve, the corresponding Hill coefficient was derived as a key parameter.

## Supporting information

S1 Fig Schematic diagram of CRISPR/Cas9 gene-editing system to construct *Dscam1* mutant flies. **Related to Fig 1. (A-C)** Schematic diagram of the construction of mutants with variable exon 4, 6, or 9 deletion, respectively. Two guide RNAs were used to perform target deletion; a nonhomologous end repair function in *Drosophila* joins the 2 cleavage sites.
(TIF)

S2 Fig The diversity of *Dscam1* exons 4, 6, and 9 is differentially required for *Drosophila* survival. **Related to Fig 2. (A-C)** Survival rates of wild-type and *Dscam*^Δ4.x-4.x’^ (A), *Dscam*^Δ6.y-6.y’^ (B), and *Dscam*^Δ9.z-9.z’^ (C) mutants. The number of remaining variable exons of each mutant is shown on the bottom. Data are expressed as mean ± SD. ns, not significant; *P < 0.05; **P < 0.01; ***P < 0.001. (Student *t* test, two-tailed). Data used to generate graphs can be found in S1 Data.
(TIF)

S3 Fig The diversity of *Dscam1* exons 4, 6, and 9 is differentially required for fly locomotion. **Related to Fig 2. (A-C)** The climbing ability of wild-type and *Dscam*^Δ4.x-4.x’^ (A), *Dscam*^Δ6.y-6.y’^ (B), and *Dscam*^Δ9.z-9.z’^ (C) mutants. The number of remaining variable exons of each mutant is shown on the bottom. Data are expressed as mean ± SD. ns, not significant; *P < 0.05; ***P < 0.001. (Student *t* test, two-tailed). Data used to generate graphs can be found in S1 Data.
(TIF)

S4 Fig Dscam1 diversity is dispensable for dendritic repulsion of class I da neurons. **Related to Fig 3. (A)** Schematic diagram of class I neurons. Representative images of dendrites self-repulsion of class I neurons of different mutant flies. Scale bars, 50 μm. **(B, C)** Self-repulsion of the class I dendrites in all *Dscam1* mutant flies was similar to the wild-type control. Numbers in parentheses refer to the investigated neurons of each genotype. ns, not significant. (Student *t* test, two-tailed). Data used to generate graphs can be found in S1 Data. da, dendritic arborization; Dscam1, Down syndrome cell adhesion molecule 1; WT, wild type.
(TIF)

S5 Fig Reducing Dscam1 diversity leads to defects in MB neuron development. **Related to Fig 4. (A)** The correlation between the normal MB phenotype rate and variable exon 4. **(B)** Comparison of MB phenotypes between mutants with similar Dscam1 diversity. **(C)** MB phenotypes of *Dscam*^Δ4.x-4.x’^*/Dscam*^*+*^, *Dscam*^Δ6.y-6.y’^*/Dscam*^*+*^, and *Dscam*^Δ9.z-9.z’^*/Dscam*^+^ heterozygous mutants, which were indistinguishable with the wild type. Numbers in bottom refer to the analyzed MB neurons of each genotype. Data used to generate graphs can be found in S1 Data. Dscam1, Down syndrome cell adhesion molecule 1; MB, mushroom body.
(TIF)

S6 Fig MB axonal phenotypes of Dscam1 mutants in single-cell clones. **Related to Fig 4. (A)** Summary of the different defective phenotypes of MB neurons in Dscam1 mutants. Single-cell clones were labeled by GFP (green), and MB lobes were immunostained with anti-FasII (red). Scale bar, 20 μm. **(B)** Quantification of axonal defects in single-cell clones of Dscam1 mutants. Numbers in parentheses represent the number of single neurons examined in each genotype. The remaining variable exon number and ectodomains are shown on the bottom. Data used to generate graphs can be found in S1 Data. Dscam1, Down syndrome cell adhesion molecule 1; MB, mushroom body.
(TIF)

S7 Fig Reducing Dscam1 diversity affects axonal branching in MS neurons. **Related to Fig 5. (A)** Schematic of the pSc neuron. Different colors of branch segments were assigned for scoring. Representative images of WT and Dscam1 mutant flies. Scale bar, 50 μm. **(B)** Quantitative analysis of the total branch number of pSc neurons in WT and Dscam1 mutants. Numbers in parentheses represent the number of pSc neurons observed in each genotype. ns, not significant; *P < 0.05; **P < 0.01; ***P < 0.001. (Student *t* test, two-tailed). **(C)** The average branch number of pSc neurons positively correlated with the number of variable exon 4, exon 6, and exon 9. **(D)** Comparison of phenotype–diversity correlates among variable exon 4, exon 6, and exon 9 clusters were shown. Data used to generate graphs can be found in S1 Data. Dscam1, Down syndrome cell adhesion molecule 1; MS, mechanosensory; WT, wild type.
(TIF)

S8 Fig Axonal branching comparisons of MS neurons in Dscam1 mutants. **Related to Fig 5. (A, B)** Pairwise comparisons (one-way ANOVA with Tukey’s test) of MS lateroanterior branch length (a) or total branch numbers (b) in *Dscam*^Single4.x^ mutants. ns, not significant; *p < 0.05; **p < 0.01; ***p < 0.001. **(C, D)** Comparison of the lateroanterior branch length of MS neurons in mutants with similar Dscam1 diversity. **p < 0.01; ***p < 0.001 (Student *t* test, two-tailed). Data used to generate graphs can be found in S1 Data. Dscam1, Down syndrome cell adhesion molecule 1; MS, mechanosensory; WT, wild type.
(TIF)

S9 Fig Branching patterns of posterior scutellar neurons in *Dscam1* mutants. **Related to Fig 5. (A)** Average branching patterns of each branch of MS neuron for each genotype. **(B)** Summary of the detail frequency of each branch of MS neuron in each mutant and wild type. Dscam1, Down syndrome cell adhesion molecule 1; MS, mechanosensory; WT, wild type.
(TIF)

S10 Fig Reducing Dscam1 expression level partially rescued the defects of fly viability and climbing ability in *Dscam1* mutants. **Related to Fig 6. (A)** *Dscam*^Single6.y-6.y’^/*Dscam*^null^ and *Dscam*^Single9.z-9.z’^/*Dscam*^null^ flies reduced Dscam1 expression level by approximately 50% to *Dscam*^Single6.y-6.y’^ and *Dscam*^Single9.z-9.z’^ mutants. Semiquantitative western blot analysis was used to compare Dscam1 protein levels between different genotypes. Dscam1 levels were normalized to β-actin, and the expression levels were then compared to the value of wild type, which was set to 1. **(B)** Reducing Dscam1 expression level partially rescued the diminished fly viability in homozygous mutants. **(C)** Reducing Dscam1 expression level partially rescued the diminished climbing ability in homozygous mutants. Data are expressed as mean ± SD. *P < 0.05; **P < 0.01; ***P < 0.001; ns, not significant (Student *t* test, two-tailed). Data used to generate graphs can be found in S1 Data. Dscam1, Down syndrome cell adhesion molecule 1; WT, wild type.
(TIF)

S11 Fig Reducing Dscam1 expression level reduced the neuronal defects of homozygous mutants. **Related to Fig 6. (A-C)** Comparison of the various phenotypes (class I-III overlaps (A), MB defects (B), and lateroanterior branch length of pSc neurons (C)) between mutants with 2 copies and 1 copy of Dscam1. Reducing Dscam1 level rescued defects caused by reduced diversity in homozygous mutants. ns, not significant; *P < 0.05; **P < 0.01; ***P < 0.001 (Student *t* test, two-tailed). Data used to generate graphs can be found in S1 Data. Dscam1, Down syndrome cell adhesion molecule 1; MB, mushroom body; WT, wild type.
(TIF)

S12 Fig **Comparison of Dscam1 phenotype–diversity correlations between our present study (A-C) and previous study [24] (D-F).** These down panels (D-F) were drawn from data in previous study [24], which showed around 5,000 isoforms are sufficient to pattern all 3 neuron systems analyzed (panels D-F). However, our data indicated that up to 10,000 isoforms are required for normal axon patterning in MB and MS neurons (panels B, C), while 2,000 isoforms are sufficient for normal dendrite self/non-self discrimination (panel A). Our results demonstrate that previous reports [24] have overestimated the true number of isoforms required for normal dendrite self/non-self discrimination but have underestimated the true number of isoforms required for normal axon patterning in MB and MS neurons. Moreover, inconsistent with their notion that Dscam1 diversity function was independent of the identity of the isoforms [24], our present data indicated that the function of Dscam diversity was associated with specific exon clusters or isoforms. Data used to generate graphs can be found in S1 Data. Dscam1, Down syndrome cell adhesion molecule 1; MB, mushroom body; MS, mechanosensory; WT, wild type.
(TIF)

S1 Table Summary of specific primers used for sgRNA, mutant screening, and RT-PCR. (PDF)

S2 Table Summary of the mutation sequences of Dscam1 mutants. (PDF)

S1 Raw Images Uncropped blots and minimally adjusted images for Figs 1B, 1C, 6F, 6G and S10A. (PDF)

S1 Data Data that underlie Figs 2A–2G, 3B–3D, 4B–4D, 5B–5D, 6A–6E, 6H and 6I, 7A, 7D–7I, S2A–S2C, S3A–S3C, S4B, S4C, S5A–S5C, S6B, S7B–S7D, S8A–S8D, S10B, S10C, S11A–S11C and S12A–S12C. (XLSX)

## Abbreviations

BSA: bovine serum albumin
da: dendritic arborization
Dscam1: Down syndrome cell adhesion molecule 1
HRP: horseradish peroxidase
MARCM: mosaic analysis with a repressible cell marker
MB: mushroom body
MS: mechanosensory
PBS: phosphate-buffered saline
Pcdh: protocadherin
RNAi: RNA interference
RT-PCR: reverse transcription PCR
VNC: ventral nerve cord
