# Supplementary figures and images for: A systematic CRISPR screen reveals redundant and specific roles for Dscam1 isoform diversity in neuronal wiring
Source: PLoS Biol. 2023 Jul 6;21(7):e3002197. doi: 10.1371/journal.pbio.3002197 (PMC10325099; doi:10.1371/journal.pbio.3002197)

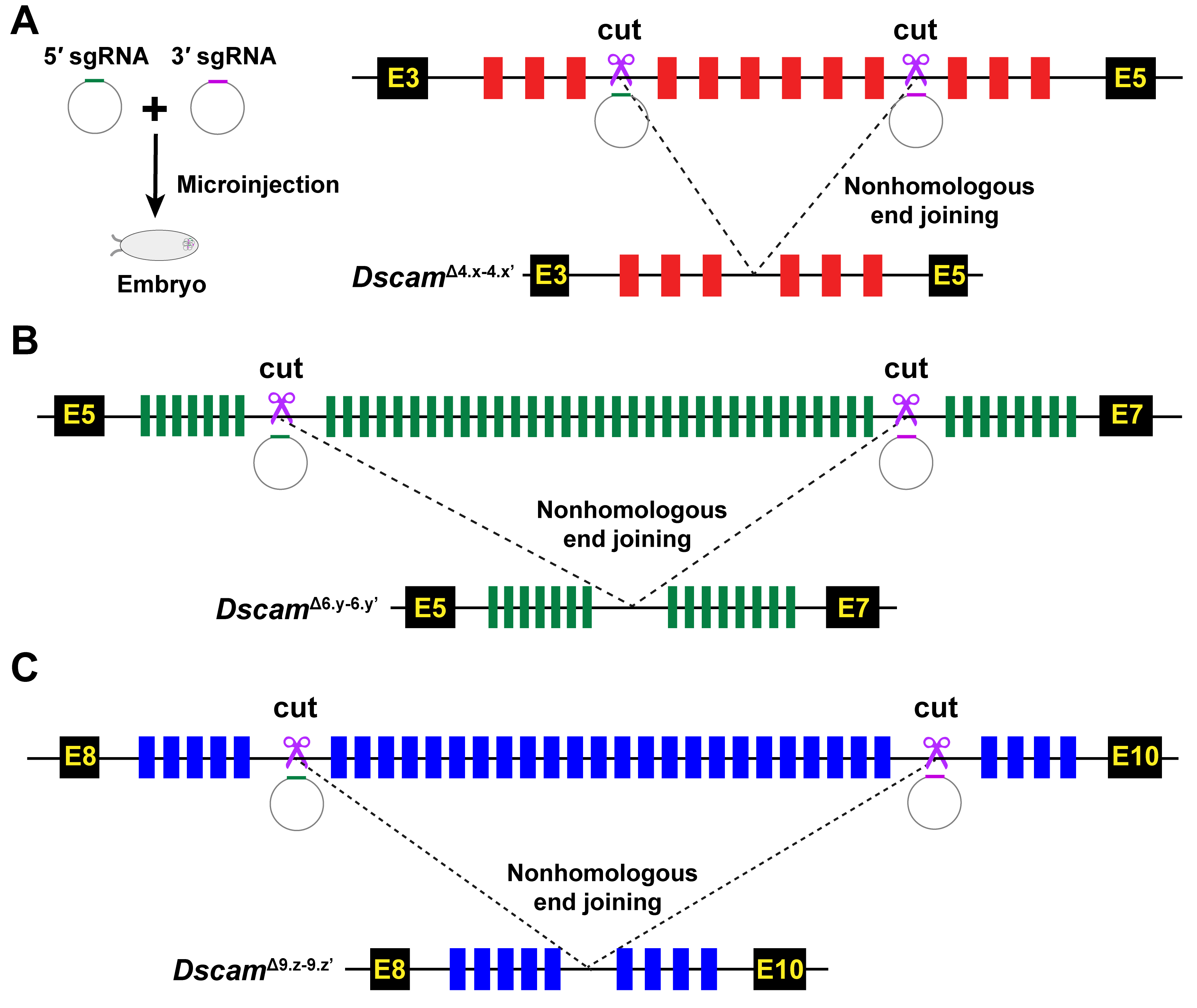

Supplement: S1 Fig — Related to Fig 1. (A-C) Schematic diagram of the construction of mutants with variable exon 4, 6, or 9 deletion, respectively. Two guide RNAs were used to perform target deletion; a nonhomologous end repair function in Drosophila joins the 2 cleavage sites. (TIF) [file pbio.3002197.s001.tif]

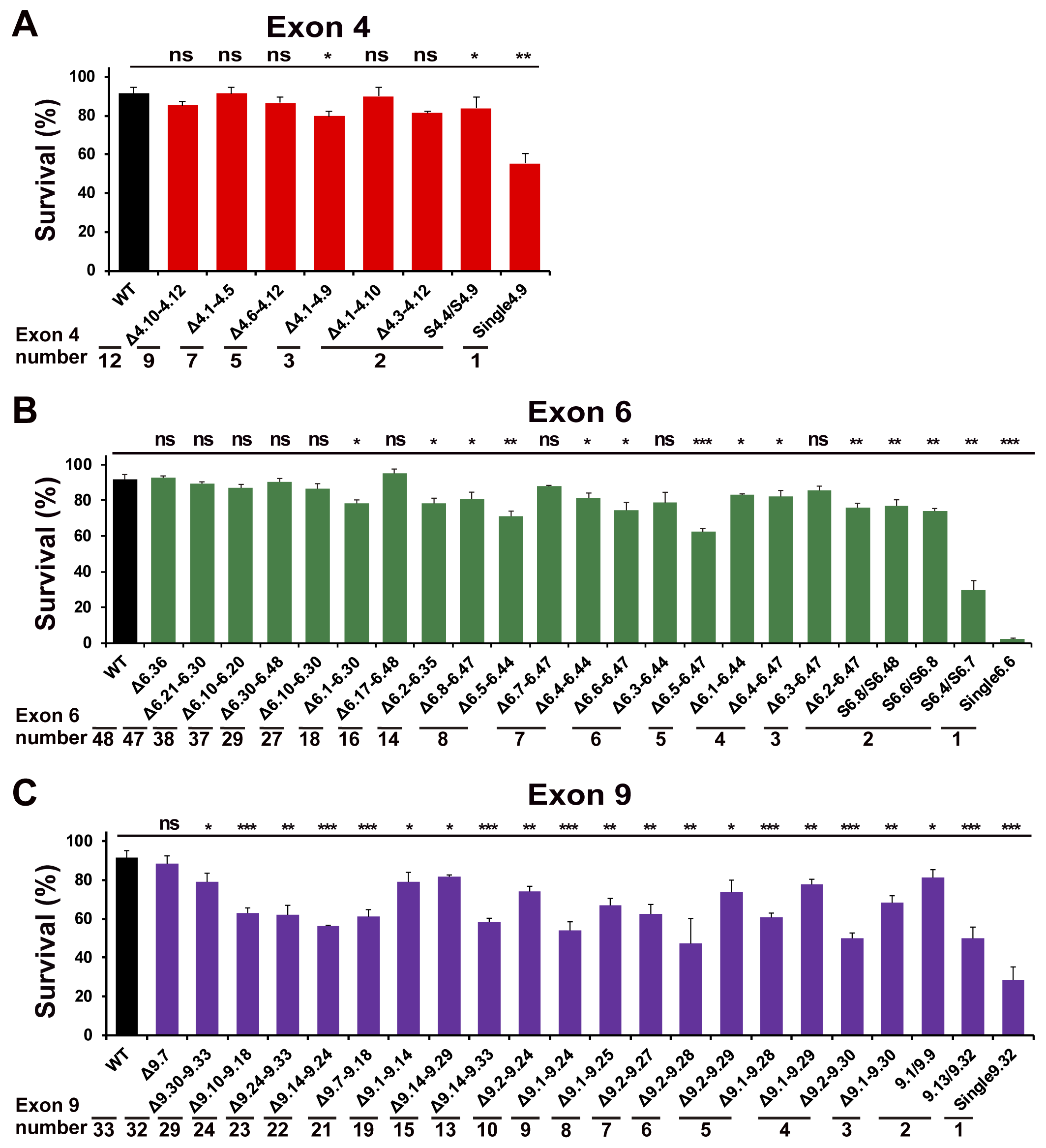

Supplement: S2 Fig — Related to Fig 2. (A-C) Survival rates of wild-type and DscamΔ4.x-4.x’ (A), DscamΔ6.y-6.y’ (B), and DscamΔ9.z-9.z’ (C) mutants. The number of remaining variable exons of each mutant is shown on the bottom. Data are expressed as mean ± SD. ns, not significant; *P < 0.05; **P < 0.01; ***P < 0.001. (Student t test, two-tailed). Data used to generate graphs can be found in S1 Data. (TIF) [file pbio.3002197.s002.tif]

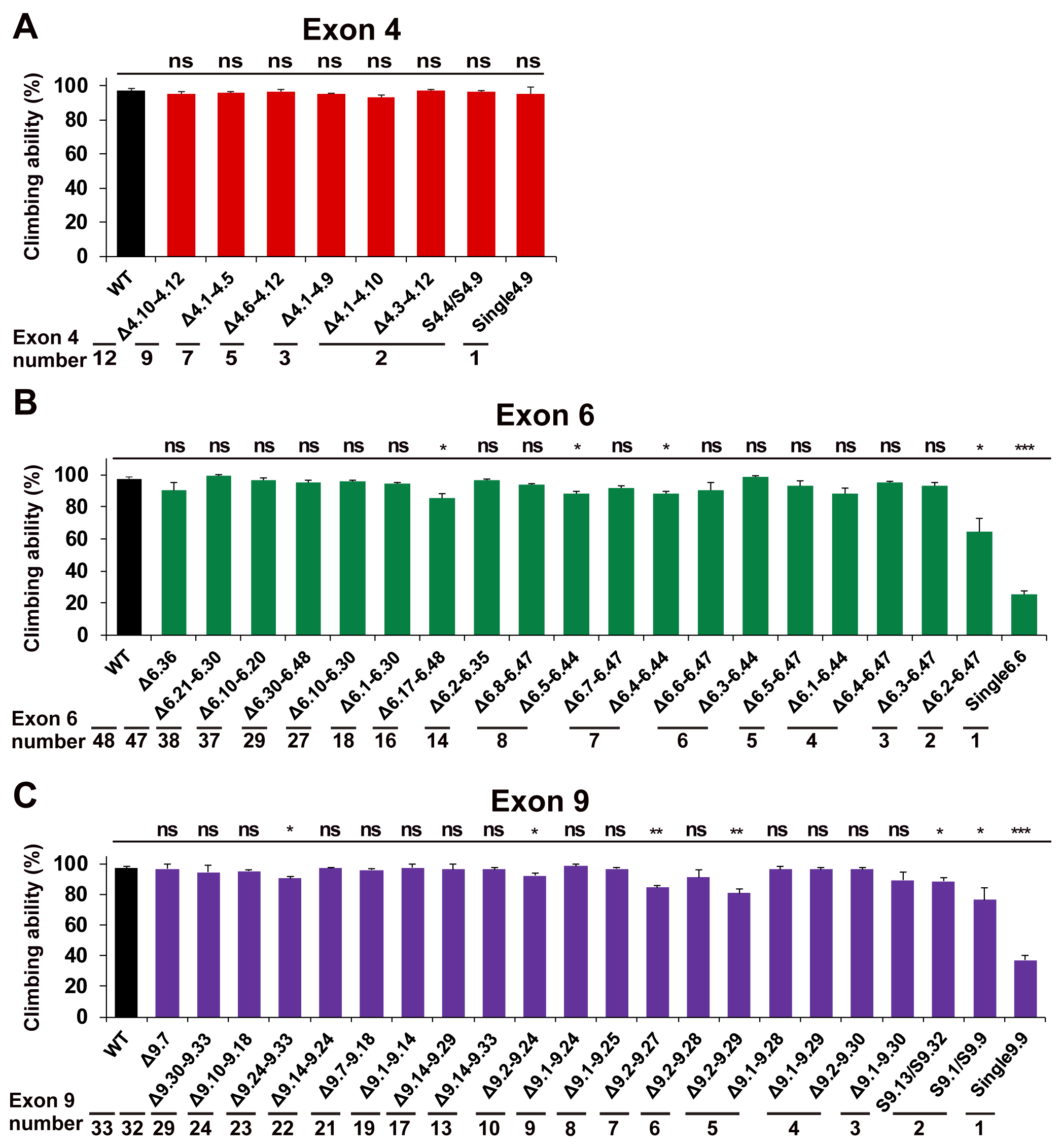

Supplement: S3 Fig — Related to Fig 2. (A-C) The climbing ability of wild-type and DscamΔ4.x-4.x’ (A), DscamΔ6.y-6.y’ (B), and DscamΔ9.z-9.z’ (C) mutants. The number of remaining variable exons of each mutant is shown on the bottom. Data are expressed as mean ± SD. ns, not significant; *P < 0.05; ***P < 0.001. (Student t test, two-tailed). Data used to generate graphs can be found in S1 Data. (TIF) [file pbio.3002197.s003.tif]

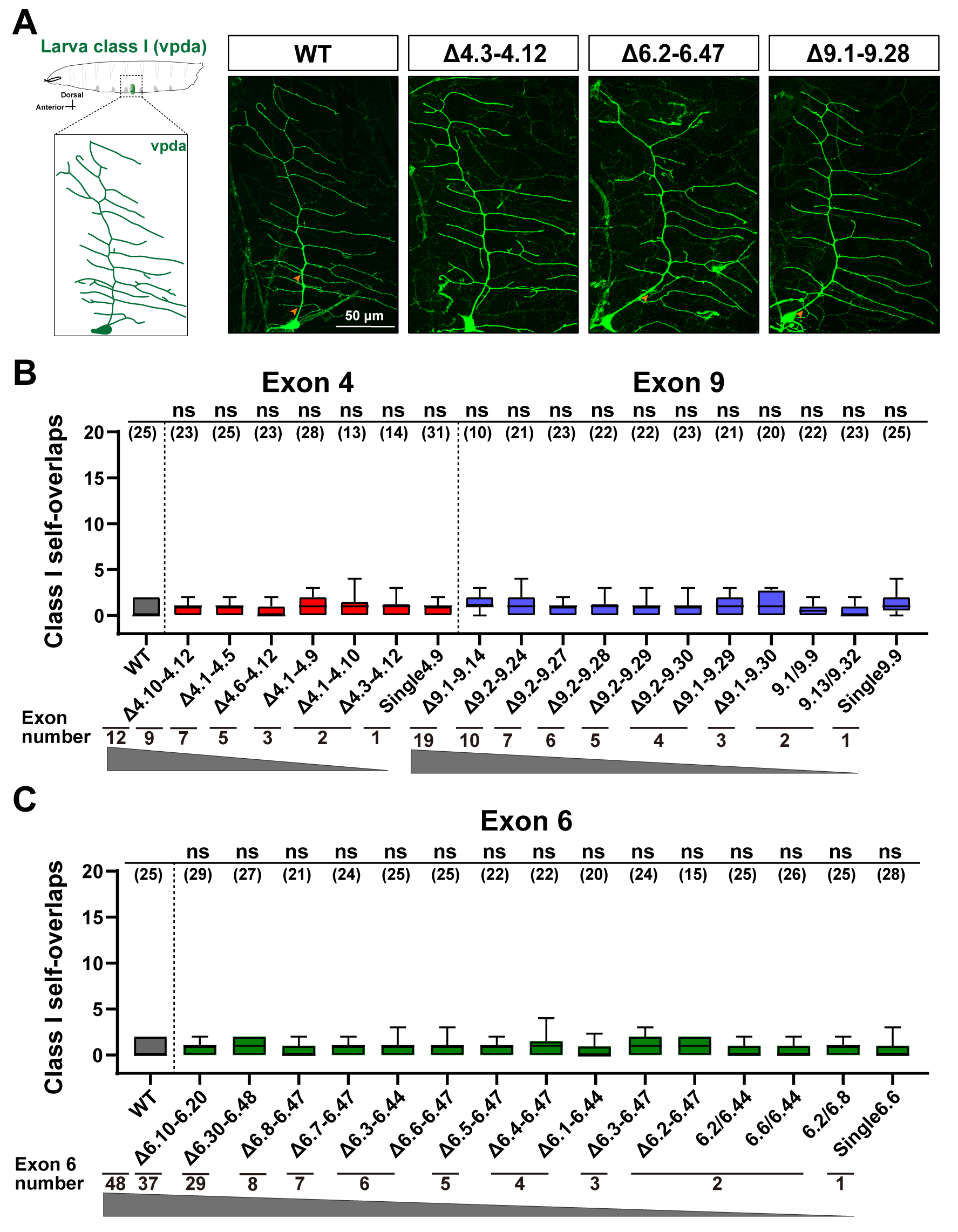

Supplement: S4 Fig — Related to Fig 3. (A) Schematic diagram of class I neurons. Representative images of dendrites self-repulsion of class I neurons of different mutant flies. Scale bars, 50 μm. (B, C) Self-repulsion of the class I dendrites in all Dscam1 mutant flies was similar to the wild-type control. Numbers in parentheses refer to the investigated neurons of each genotype. ns, not significant. (Student t test, two-tailed). Data used to generate graphs can be found in S1 Data. da, dendritic arborization; Dscam1, Down syndrome cell adhesion molecule 1; WT, wild type. (TIF) [file pbio.3002197.s004.tif]

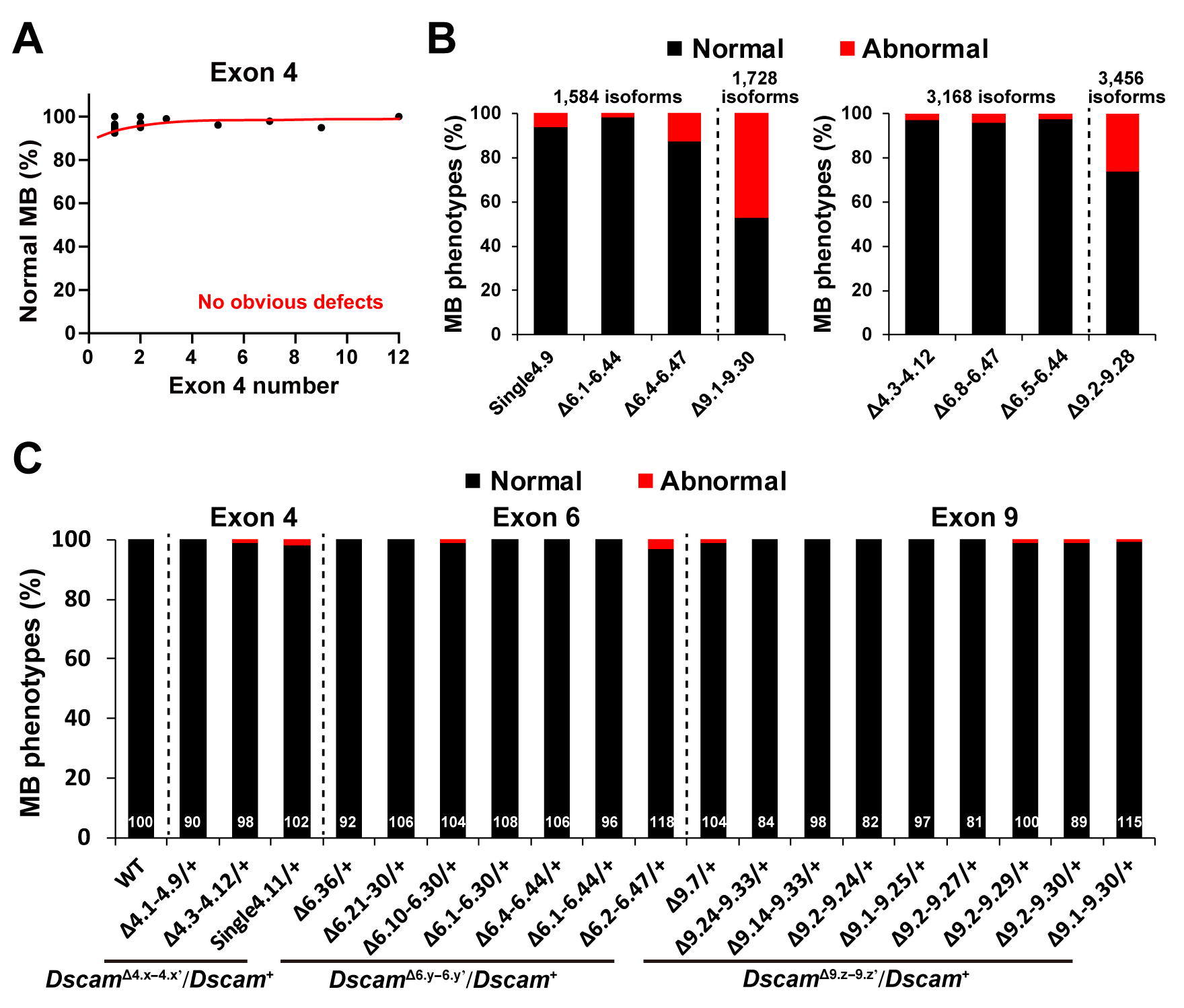

Supplement: S5 Fig — Related to Fig 4. (A) The correlation between the normal MB phenotype rate and variable exon 4. (B) Comparison of MB phenotypes between mutants with similar Dscam1 diversity. (C) MB phenotypes of DscamΔ4.x-4.x’/Dscam+, DscamΔ6.y-6.y’/Dscam+, and DscamΔ9.z-9.z’/Dscam+ heterozygous mutants, which were indistinguishable with the wild type. Numbers in bottom refer to the analyzed MB neurons of each genotype. Data used to generate graphs can be found in S1 Data. Dscam1, Down syndrome cell adhesion molecule 1; MB, mushroom body. (TIF) [file pbio.3002197.s005.tif]

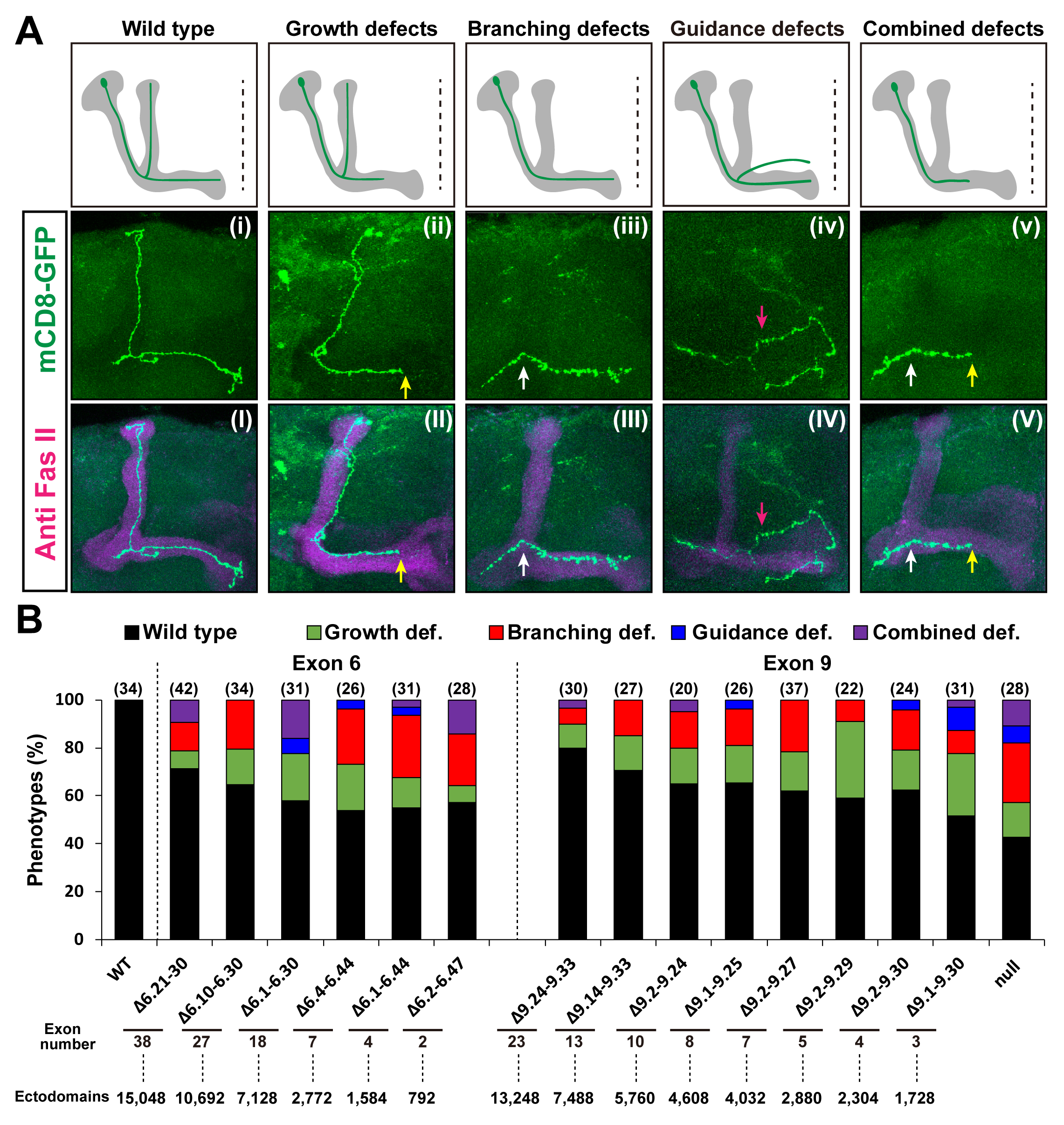

Supplement: S6 Fig — Related to Fig 4. (A) Summary of the different defective phenotypes of MB neurons in Dscam1 mutants. Single-cell clones were labeled by GFP (green), and MB lobes were immunostained with anti-FasII (red). Scale bar, 20 μm. (B) Quantification of axonal defects in single-cell clones of Dscam1 mutants. Numbers in parentheses represent the number of single neurons examined in each genotype. The remaining variable exon number and ectodomains are shown on the bottom. Data used to generate graphs can be found in S1 Data. Dscam1, Down syndrome cell adhesion molecule 1; MB, mushroom body. (TIF) [file pbio.3002197.s006.tif]

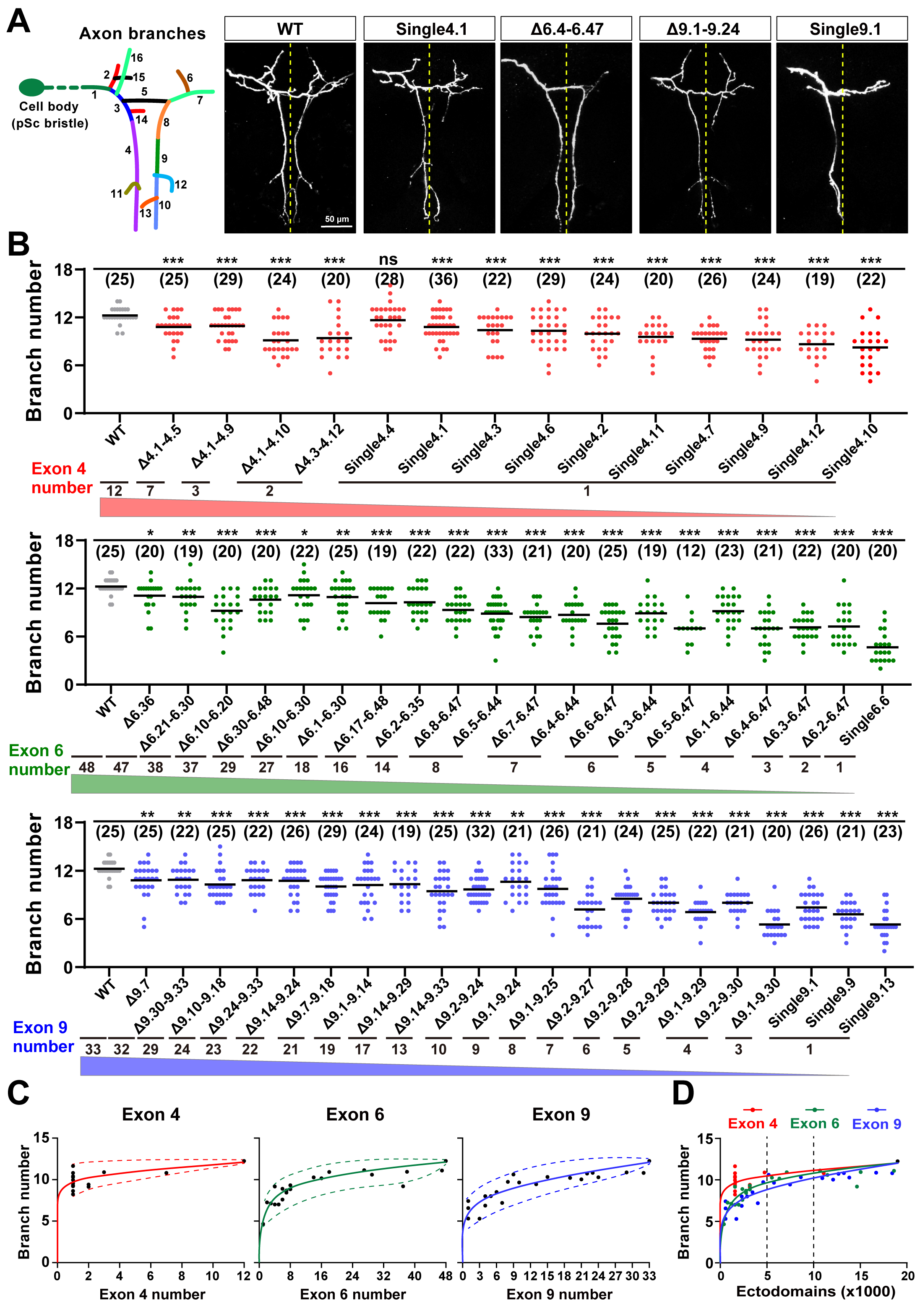

Supplement: S7 Fig — Related to Fig 5. (A) Schematic of the pSc neuron. Different colors of branch segments were assigned for scoring. Representative images of WT and Dscam1 mutant flies. Scale bar, 50 μm. (B) Quantitative analysis of the total branch number of pSc neurons in WT and Dscam1 mutants. Numbers in parentheses represent the number of pSc neurons observed in each genotype. ns, not significant; *P < 0.05; **P < 0.01; ***P < 0.001. (Student t test, two-tailed). (C) The average branch number of pSc neurons positively correlated with the number of variable exon 4, exon 6, and exon 9. (D) Comparison of phenotype–diversity correlates among variable exon 4, exon 6, and exon 9 clusters were shown. Data used to generate graphs can be found in S1 Data. Dscam1, Down syndrome cell adhesion molecule 1; MS, mechanosensory; WT, wild type. (TIF) [file pbio.3002197.s007.tif]

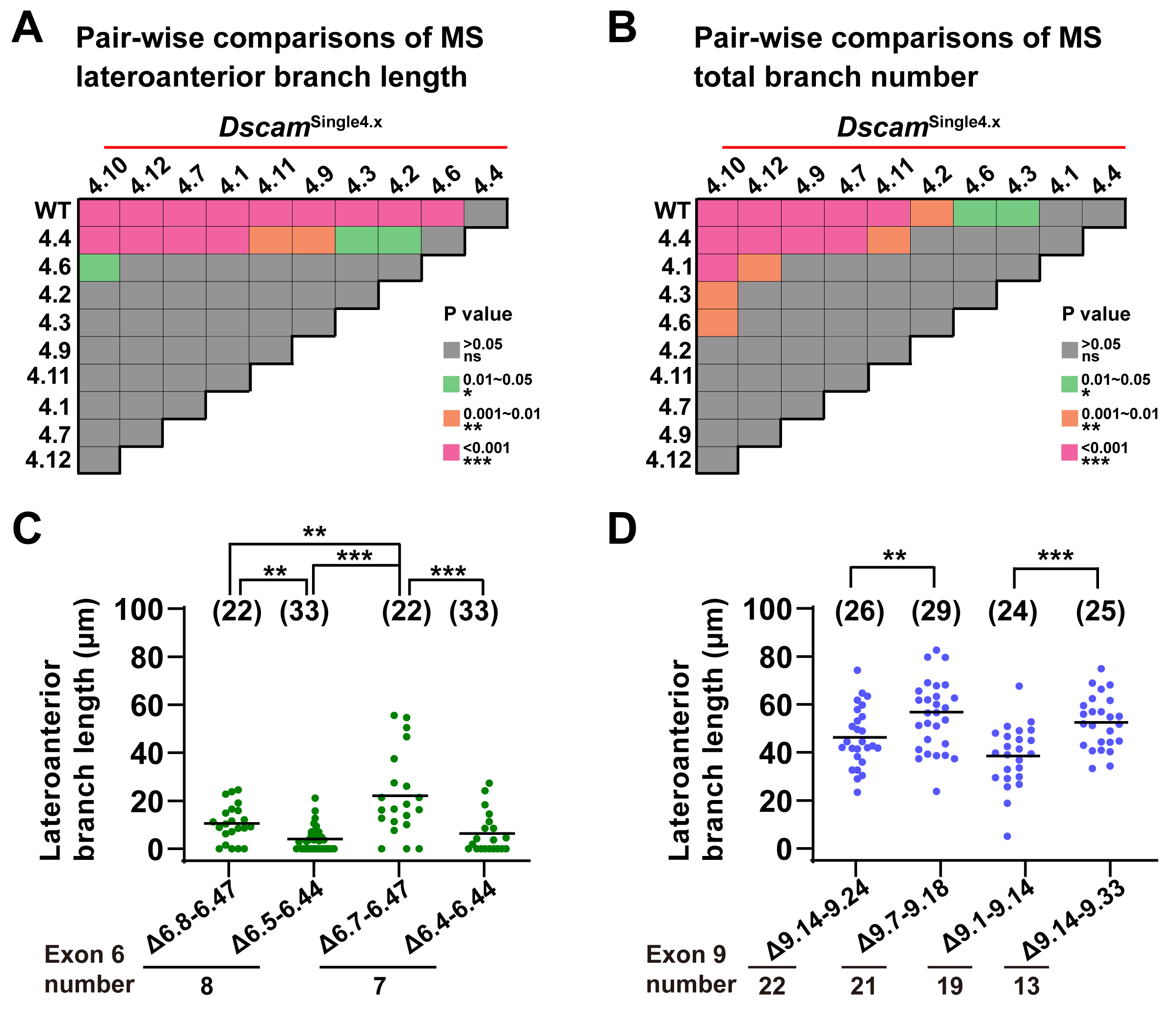

Supplement: S8 Fig — Related to Fig 5. (A, B) Pairwise comparisons (one-way ANOVA with Tukey’s test) of MS lateroanterior branch length (a) or total branch numbers (b) in DscamSingle4.x mutants. ns, not significant; *p < 0.05; **p < 0.01; ***p < 0.001. (C, D) Comparison of the lateroanterior branch length of MS neurons in mutants with similar Dscam1 diversity. **p < 0.01; ***p < 0.001 (Student t test, two-tailed). Data used to generate graphs can be found in S1 Data. Dscam1, Down syndrome cell adhesion molecule 1; MS, mechanosensory; WT, wild type. (TIF) [file pbio.3002197.s008.tif]

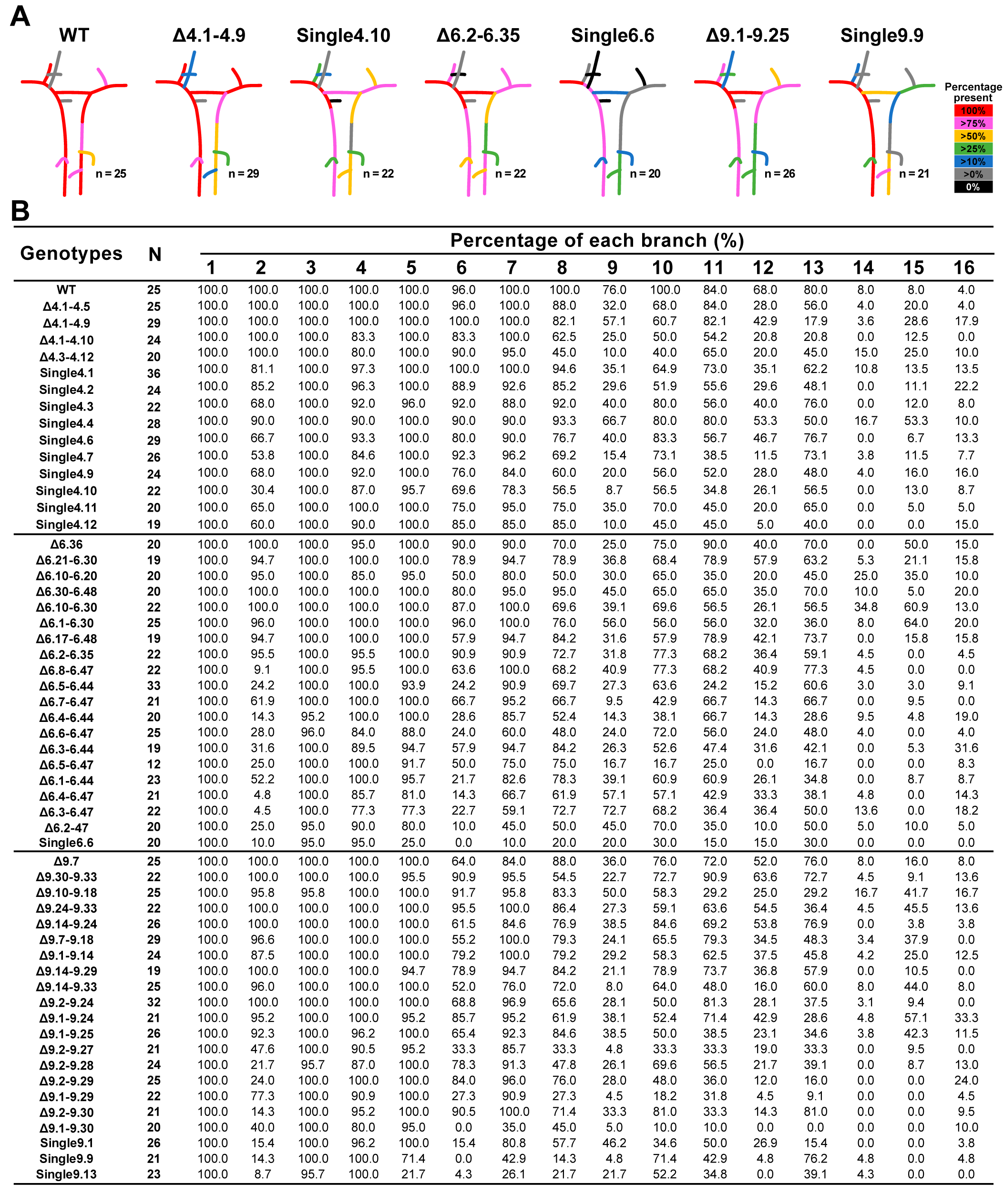

Supplement: S9 Fig — Related to Fig 5. (A) Average branching patterns of each branch of MS neuron for each genotype. (B) Summary of the detail frequency of each branch of MS neuron in each mutant and wild type. Dscam1, Down syndrome cell adhesion molecule 1; MS, mechanosensory; WT, wild type. (TIF) [file pbio.3002197.s009.tif]

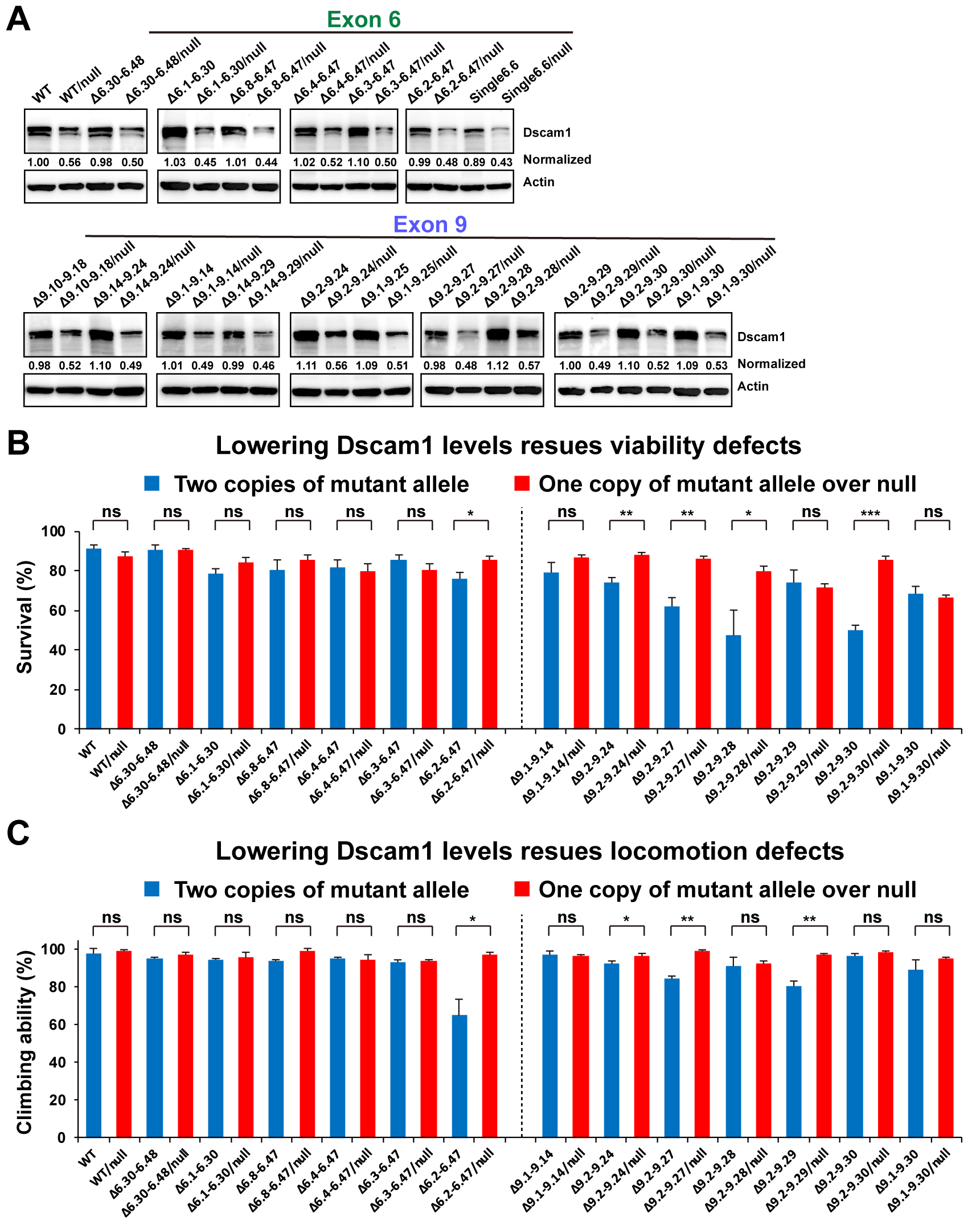

Supplement: S10 Fig — Related to Fig 6. (A) DscamSingle6.y-6.y’/Dscamnull and DscamSingle9.z-9.z’/Dscamnull flies reduced Dscam1 expression level by approximately 50% to DscamSingle6.y-6.y’ and DscamSingle9.z-9.z’ mutants. Semiquantitative western blot analysis was used to compare Dscam1 protein levels between different genotypes. Dscam1 levels were normalized to β-actin, and the expression levels were then compared to the value of wild type, which was set to 1. (B) Reducing Dscam1 expression level partially rescued the diminished fly viability in homozygous mutants. (C) Reducing Dscam1 expression level partially rescued the diminished climbing ability in homozygous mutants. Data are expressed as mean ± SD. *P < 0.05; **P < 0.01; ***P < 0.001; ns, not significant (Student t test, two-tailed). Data used to generate graphs can be found in S1 Data. Dscam1, Down syndrome cell adhesion molecule 1; WT, wild type. (TIF) [file pbio.3002197.s010.tif]

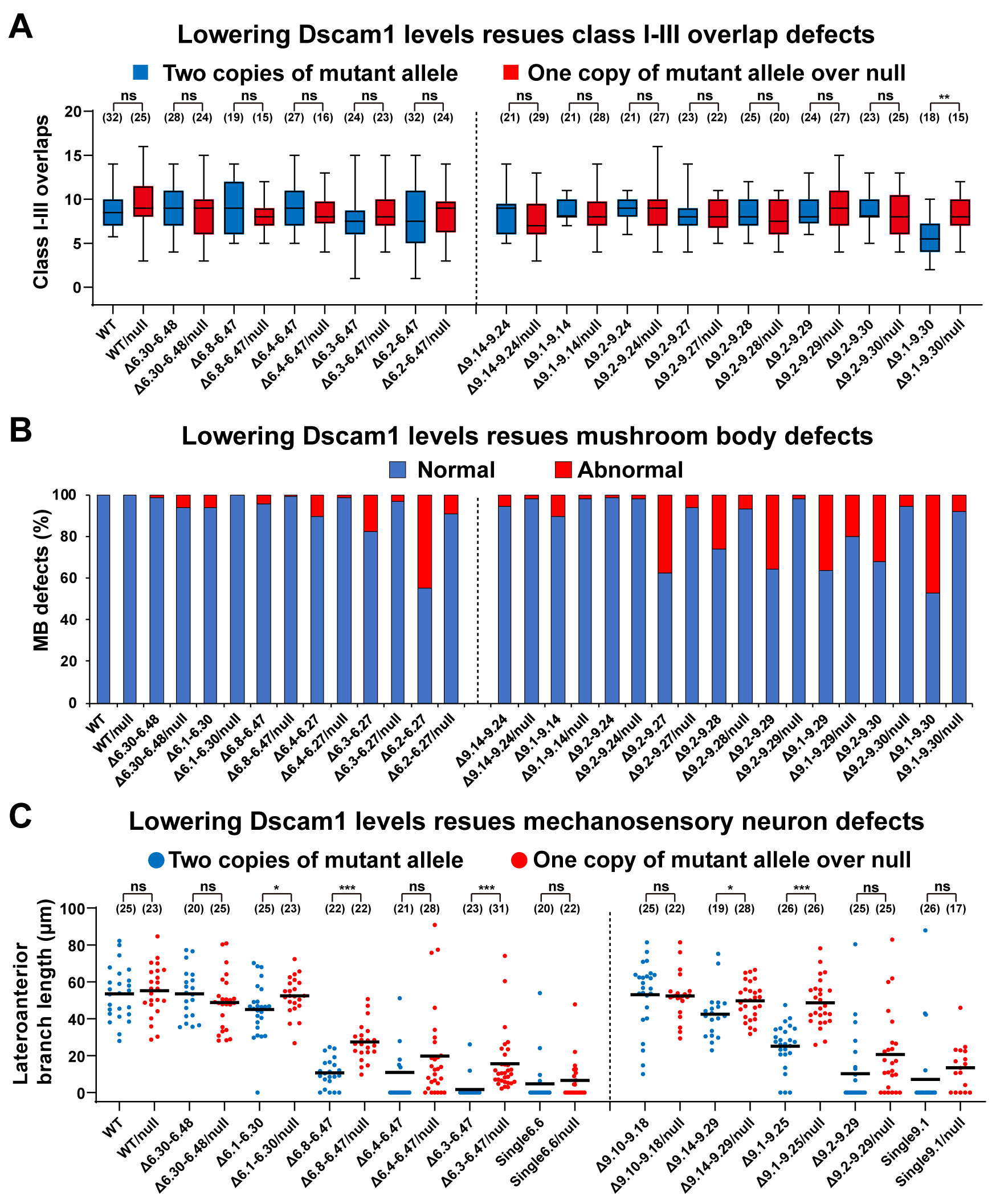

Supplement: S11 Fig — Related to Fig 6. (A-C) Comparison of the various phenotypes (class I-III overlaps (A), MB defects (B), and lateroanterior branch length of pSc neurons (C)) between mutants with 2 copies and 1 copy of Dscam1. Reducing Dscam1 level rescued defects caused by reduced diversity in homozygous mutants. ns, not significant; *P < 0.05; **P < 0.01; ***P < 0.001 (Student t test, two-tailed). Data used to generate graphs can be found in S1 Data. Dscam1, Down syndrome cell adhesion molecule 1; MB, mushroom body; WT, wild type. (TIF) [file pbio.3002197.s011.tif]

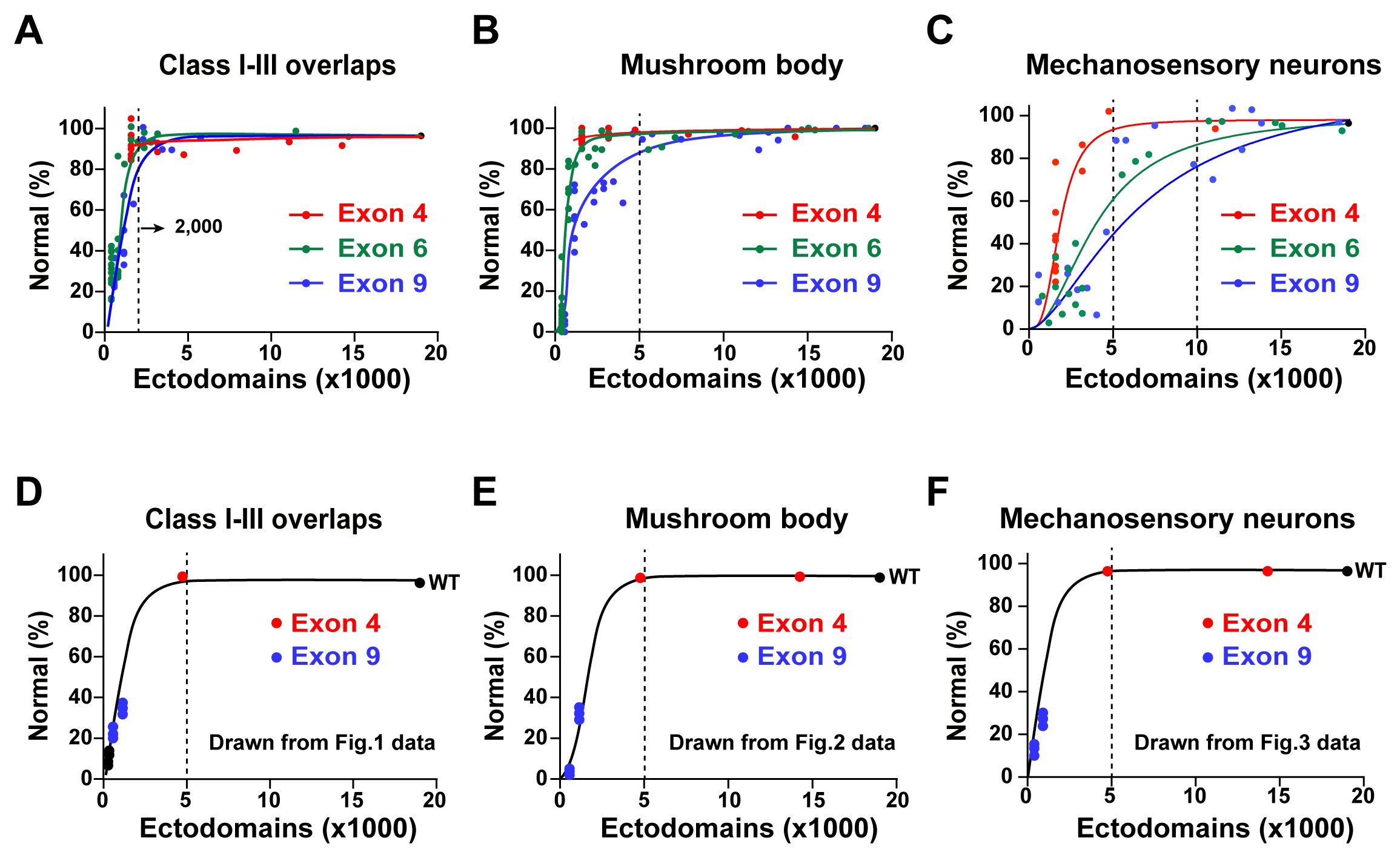

Supplement: S12 Fig — Comparison of Dscam1 phenotype–diversity correlations between our present study (A-C) and previous study [24] (D-F). These down panels (D-F) were drawn from data in previous study [24], which showed around 5,000 isoforms are sufficient to pattern all 3 neuron systems analyzed (panels D-F). However, our data indicated that up to 10,000 isoforms are required for normal axon patterning in MB and MS neurons (panels B, C), while 2,000 isoforms are sufficient for normal dendrite self/non-self discrimination (panel A). Our results demonstrate that previous reports [24] have overestimated the true number of isoforms required for normal dendrite self/non-self discrimination but have underestimated the true number of isoforms required for normal axon patterning in MB and MS neurons. Moreover, inconsistent with their notion that Dscam1 diversity function was independent of the identity of the isoforms [24], our present data indicated that the function of Dscam diversity was associated with specific exon clusters or isoforms. Data used to generate graphs can be found in S1 Data. Dscam1, Down syndrome cell adhesion molecule 1; MB, mushroom body; MS, mechanosensory; WT, wild type. (TIF) [file pbio.3002197.s012.tif]
