# Supplementary material for: A systematic CRISPR screen reveals redundant and specific roles for Dscam1 isoform diversity in neuronal wiring
Source: PLoS Biol. 2023 Jul 6;21(7):e3002197. doi: 10.1371/journal.pbio.3002197 (PMC10325099; doi:10.1371/journal.pbio.3002197)
Supplement: S1 Table — (PDF) [file pbio.3002197.s013.pdf]

Table S1 Specific primers used for sgRNA and mutant screening		
**Mutants**	**Screening primers**	**sgRNA Primers**
Dscam$^{\Delta 4.10\text{-}4.12}$	**Ds-4.10-F:** AGTTCTACATAACCGAGGCC **Ds-4.12-R:** CTCCAGAGGGCAATACCAGG	**Ds-4.10-sg-F1:** TAGCTCTCTCAGGCTCCTCT **Ds-4.12-sg-F1:** AACCCTTTTAGTTTTCCCC
Dscam$^{\Delta 4.1\text{-}4.5}$	**Ds-4.1-F:** TATTGTTGCACCGGCTGCGG **Ds-4.5-R:** CGGCCTCGGTTATGTAGAAC	**Ds-4.1-sg-F1:** TCTACACCCTGAACACGGGC **Ds-4.5-sg-F1:** TCCCCATTTCAATTTGCTTC
Dscam$^{\Delta 4.6\text{-}4.12}$	**Ds-4.6-F:** GCAGTTCTATGAATCGGAGG **Ds-4.12-R:** GCTGCTAATCGTTGACCATG	**Ds-4.5-sg-F1:** TCCCCATTTCAATTTGCTTC **Ds-4.12-sg-F1:** AACCCTTTTAGTTTTCCCC
Dscam$^{\Delta 4.1\text{-}4.9}$	**Ds-4.1-F:** TTCCAGGAACTCAGTTCCTAAGCAC **Ds-4.9-R:** CACATACTCGTCGTAGACTTGCGAC	**Ds-4.1-sg-F2:** GGTTGCCCAGTACTACGAGG **Ds-4.9-sg-F1:** GTGCGAGATTCCCTCCTACG
Dscam$^{\Delta 4.1\text{-}4.10}$	**Ds-4.1-F:** TTCCAGGAACTCAGTTCCTAAGCAC **Ds-4.10-R:** CACATACTCGTCGTAGACTTGCGAC	**Ds-4.1-sg-F2:** GGTTGCCCAGTACTACGAGG **Ds-4.10-sg-F2:** CCCCTCCCGGACGACTCCGT
Dscam$^{\Delta 4.3\text{-}4.12}$	**Ds-4.3-F:** TGCGATATACCGTCGTTTGTAGCCG **Ds-4.12-R:** ACATCGAGACCAGATCGAAGCGAAC	**Ds-4.3-sg-F1:** TGTAATACGCGGCAATGCGG **Ds-4.12-sg-F1:** CGATTCGTGGGTGGAGGACG
Dscam$^{\Delta 6.36}$	**Ds-6.36-F:** CACTTCCCCACACTCTCATAC **Ds-6.36-R:** ATGGCAAAAGTGGAGAGCAGTG	**Ds-6.36-sg-F1:** AGGGAGAGTCGCGGTGCACA **Ds-6.36-sg-F2:** TTCCAGTGCCCATCATTAGG
Dscam$^{\Delta 6.21\text{-}6.30}$	**Ds-6.21-F:** CTCTTCTCTGTCTTCGTCAGG **Ds-6.30-R:** GAGCATTGATTTTGGGCGGAC	**Ds-6.21-sg-F1:** TGAGTACAATCCTACAGATC **Ds-6.30-sg-F1:** ATGTCCGGCGCAAGGCTACC
Dscam$^{\Delta 6.10\text{-}6.20}$	**Ds-6.10-F:** CCTTTCCCAACATCATCATCAG **Ds-6.20-F:** GGGTTCTGTGGGAGGTTTTATC	**Ds-6.10-sg-F1:** GCAGACACGCGAGATTTCCC **Ds-6.21-sg-F1:** TGAGTACAATCCTACAGATC
Dscam$^{\Delta 6.30\text{-}6.48}$	**Ds-6.30-F:** TAGAGCCTGTGGGAAGTGTGGGTC **Ds-6.48-R:** GCTTGTTTACGGGTTGTTCCTTCG	**Ds-6.30-sg-F1:** ATGTCCGGCGCAAGGCTACC **Ds-6.48-sg-F1:** GGTCCCCCATTACTAGGCCG
Dscam$^{\Delta 6.10\text{-}6.30}$	**Ds-6.10-F:** CCTTTCCCAACATCATCATCAG **Ds-6.30-R:** GAGCATTGATTTTGGGCGGAC	**Ds-6.10-sg-F1:** GCAGACACGCGAGATTTCCC **Ds-6.30-sg-F1:** ATGTCCGGCGCAAGGCTACC
Dscam$^{\Delta 6.1\text{-}6.30}$	**Ds-6.1-F:** CAAGTCCCAATCGTTGGCTATC **Ds-6.30-R:** GAGCATTGATTTTGGGCGGAC	**Ds-6.1-sg-F1:** GCTTCATTCACAGAACCAGT **Ds-6.30-sg-F1:** ATGTCCGGCGCAAGGCTACC
Dscam$^{\Delta 6.17\text{-}6.48}$	**Ds-6.16-F:** TGCCCTCCACCAACATTCACTT **Ds-6.48-R:** GCTTGTTTACGGGTTGTTCCTTCG	**Ds-6.16-sg-F1:** TCCGGTGAGTACCAGCTGTC **Ds-6.48-sg-F1:** GGTCCCCCATTACTAGGCCG
Dscam$^{\Delta 6.2\text{-}6.35}$	**Ds-6.2-F:** CAAGTCCCAATCGTTGGCTATC **Ds-6.36-R:** ATGGCAAAAGTGGAGAGCAGTG	**Ds-6.2-sg-F1:** AGACCAACTTGCGCCAGTTT **Ds-6.36-sg-F1:** AGGGAGAGTCGCGGTGCACA
Dscam$^{\Delta 6.8\text{-}6.47}$	**Ds-6.8-F:** AGTCGCGAATTCTCCGGGTGTATC **Ds-6.47-R:** GCTTGTCCCTGGCAAAGAATAGAG	**Ds-6.8-sg-F1:** GAGCGGAACTAGCTTCAACA **Ds-6.47-sg-F1:** ACACCCGCATTTAGGTAATG
Dscam$^{\Delta 6.5\text{-}6.44}$	**Ds-6.5-F:** AAACCCAACCCAACACTTAG **Ds-6.44-R:** GGCACAAAAGGGCGAAACTAGT	**Ds-6.5-sg-F1:** ATCAGGCGACGATTCGCGGA **Ds-6.44-sg-F1:** TTCAGAGCCCGTGGGCTCCA
Dscam$^{\Delta 6.7\text{-}6.47}$	**Ds-6.5-F:** AAACCCAACCCAACACTTAG **Ds-6.47-R:** GCTTGTCCCTGGCAAAGAATAGAG	**Ds-6.7-sg-F1:** TGCAGAACCAATTGGTAGCG **Ds-6.47-sg-F1:** ACACCCGCATTTAGGTAATG

Dscam$^{\Delta 6.4\text{-}6.44}$	**Ds-6.3-F:**<br>AGTCGCGAATTCTCCGGGTGTATC<br>**Ds-6.44-R:**<br>GGCACAAAAGGGCGAAACTAGT	**Ds-6.4-sg-F1:**<br>GTCGGAAGTGTAAGTCCCCA<br>**Ds-6.44-sg-F1:**<br>TTCAGAGCCCGTGGGCTCCA
Dscam$^{\Delta 6.6\text{-}6.47}$	**Ds-6.5-F:**<br>AAACCCAACCCAACACTTAG<br>**Ds-6.47-R:**<br>GCTTGTCCCTGGCAAAGAATAGAG	**Ds-6.46-sg-F1:**<br>CCCCAAGATAAGTACTGGTG<br>**Ds-6.47-sg-F1:**<br>ACACCCGCATTTAGGTAATG
Dscam$^{\Delta 6.3\text{-}6.44}$	**Ds-6.3-F:**<br>GTTTCCCAATACTCTGACCAGC<br>**Ds-6.44-R:**<br>GGCACAAAAGGGCGAAACTAGT	**Ds-6.3-sg-F1:**<br>GTTGGCAGTATTGGACCCCG<br>**Ds-6.44-sg-F1:**<br>TTCAGAGCCCGTGGGCTCCA
Dscam$^{\Delta 6.5\text{-}6.47}$	**Ds-6.5-F:**<br>AAACCCAACCCAACACTTAG<br>**Ds-6.47-R:**<br>GCTTGTCCCTGGCAAAGAATAGAG	**Ds-6.5-sg-F1:**<br>ATCAGGCGACGATTCGCGGA<br>**Ds-6.47-sg-F1:**<br>ACACCCGCATTTAGGTAATG
Dscam$^{\Delta 6.1\text{-}6.44}$	**Ds-6.1-F:**<br>CAAGTCCCAATCGTTGGCTATC<br>**Ds-6.44-R:**<br>GGCACAAAAGGGCGAAACTAGT	**Ds-6.1-sg-F1:**<br>GCTTCATTCACAGAACCAGT<br>**Ds-6.44-sg-F1:**<br>TTCAGAGCCCGTGGGCTCCA
Dscam$^{\Delta 6.4\text{-}6.47}$	**Ds-6.3-F:**<br>AGTCGCGAATTCTCCGGGTGTATC<br>**Ds-6.47-R:**<br>GCTTGTCCCTGGCAAAGAATAGAG	**Ds-6.4-sg-F1:**<br>GTCGGAAGTGTAAGTCCCCA<br>**Ds-6.47-sg-F1:**<br>ACACCCGCATTTAGGTAATG
Dscam$^{\Delta 6.3\text{-}6.47}$	**Ds-6.3-F:**<br>AGTCGCGAATTCTCCGGGTGTATC<br>**Ds-6.47-R:**<br>GCTTGTCCCTGGCAAAGAATAGAG	**Ds-6.3-sg-F2:**<br>AAGTGCAACTCTTCTCTGCC<br>**Ds-6.47-sg-F1:**<br>ACACCCGCATTTAGGTAATG
Dscam$^{\Delta 6.2\text{-}6.47}$	**Ds-6.2-F:**<br>CAAGTCCCAATCGTTGGCTATC<br>**Ds-6.47-R:**<br>GCTTGTCCCTGGCAAAGAATAGAG	**Ds-6.2-sg-F1:**<br>AGACCAACTTGCGCCAGTTT<br>**Ds-6.47-sg-F1:**<br>ACACCCGCATTTAGGTAATG
Dscam$^{\Delta 9.7}$	**Ds-9.7-F:**<br>GTACCACCTACCTGAGTGTGGAT<br>**Ds-9.7-R:**<br>CATTGGCAGATCGCCCTTGGGAAC	**Ds-9.7-sg-F1:**<br>CCTGTAAAGTTGCTTCCTAC<br>**Ds-9.7-sg-F2:**<br>CACTAGATGTCAACGGTTTG
Dscam$^{\Delta 9.30\text{-}9.33}$	**Ds-9.30-F:**<br>GCTGCGTGTGATAGGTTCTC<br>**Ds-9.33-R:**<br>ACTTGGTCTTTGGCCTACGGAT	**Ds-9.30-sg-F1:**<br>GGGAATAACCACGTCCCGCG<br>**Ds-9.33-sg-F1:**<br>GACCAGGCTAACTGTGAATG
Dscam$^{\Delta 9.10\text{-}9.18}$	**Ds-9.10-F:**<br>GTCCCTCAACTCTGCTCCAATCG<br>**Ds-9.18-R:**<br>CCTATTCCCTCATTCCTCTTAACA	**Ds-9.10-sg-F1:**<br>TTAGTATTGCTTGAGGTGTT<br>**Ds-9.18-sg-F1:**<br>CCTGATTGTGAACGGTTGGC
Dscam$^{\Delta 9.24\text{-}9.33}$	**Ds-9.24-F:**<br>TCCTCCTAAAATCACTCCCTTC<br>**Ds-9.33-R:**<br>ACTTGGTCTTTGGCCTACGGAT	**Ds-9.24-sg-F1:**<br>GGAATCCATAAGCTACGCCT<br>**Ds-9.33-sg-F1:**<br>GACCAGGCTAACTGTGAATG
Dscam$^{\Delta 9.14\text{-}9.24}$	**Ds-9.14-F:**<br>GAATCGCATTAGCGAGAAGACGAG<br>**Ds-9.24-R:**<br>GCGACGGATTTTTATAAGCATAATC	**Ds-9.14-sg-F1:**<br>ACCTGCCCATGAATGTCACC<br>**Ds-9.24-sg-F1:**<br>GGAATCCATAAGCTACGCCT
Dscam$^{\Delta 9.1\text{-}9.14}$	**Ds-9.1-F:**<br>TGCCAAGAATCAGGAAGGATACTC<br>**Ds-9.14-R:**<br>TCACGATCAGCAGTGCCGAG	**Ds-9.1-sg-F1:**<br>CCGCAGGTCGTACCCTTTGA<br>**Ds-9.14-sg-F1:**<br>ACCTGCCCATGAATGTCACC
Dscam$^{\Delta 9.14\text{-}9.29}$	**Ds-9.14-F:**<br>GAATCGCATTAGCGAGAAGACGAG<br>**Ds-9.33-R:**<br>ACTTGGTCTTTGGCCTACGGAT	**Ds-9.14-sg-F1:**<br>ACCTGCCCATGAATGTCACC<br>**Ds-9.29-sg-F1:**<br>AGCGTCTTAGTATTAAGAGA
Dscam$^{\Delta 9.14\text{-}9.33}$	**Ds-9.14-F:**<br>GAATCGCATTAGCGAGAAGACGAG<br>**Ds-9.33-R:**<br>ACTTGGTCTTTGGCCTACGGAT	**Ds-9.14-sg-F1:**<br>ACCTGCCCATGAATGTCACC<br>**Ds-9.33-sg-F1:**<br>GACCAGGCTAACTGTGAATG
Dscam$^{\Delta 9.2\text{-}9.24}$	**Ds-9.1-F:**<br>TGCCAAGAATCAGGAAGGATACTC<br>**Ds-9.24-R:**<br>GCGACGGATTTTTATAAGCATAATC	**Ds-9.2-sg-F1:**<br>CCGCAGTTCTGCCCCAGATC<br>**Ds-9.24-sg-F1:**<br>GGAATCCATAAGCTACGCCT
Dscam$^{\Delta 9.1\text{-}9.24}$	**Ds-9.1-F:**<br>TGCCAAGAATCAGGAAGGATACTC<br>**Ds-9.24-R:**<br>GCGACGGATTTTTATAAGCATAATC	**Ds-9.1-sg-F1:**<br>CCGCAGGTCGTACCCTTTGA<br>**Ds-9.24-sg-F1:**<br>GGAATCCATAAGCTACGCCT

Dscam$^{\Delta 9.1\text{-}9.25}$	**Ds-9.1-F:**<br>TGCCAAGAATCAGGAAGGATACTC<br>**Ds-9.25-R:**<br>GACAAGTCACCGAAACGGAATCC	**Ds-9.1-sg-F1:**<br>CCGCAGGTCGTACCCTTTGA<br>**Ds-9.25-sg-F1:**<br>ACTAATAGTTAATGGTACAC
Dscam$^{\Delta 9.2\text{-}9.27}$	**Ds-9.1-F:**<br>TGCCAAGAATCAGGAAGGATACTC<br>**Ds-9.27-R:**<br>TGGGCAATCAAAGGGTATTGT	**Ds-9.2-sg-F1:**<br>CCGCAGTTCTGCCCCAGATC<br>**Ds-9.27-sg-F1:**<br>GCGAAGTCAGAGCTTCTGTA
Dscam$^{\Delta 9.2\text{-}9.28}$	**Ds-9.1-F:**<br>TGCCAAGAATCAGGAAGGATACTC<br>**Ds-9.29-R:**<br>CTCCGCCGGGAACGGAGCAGTG	**Ds-9.2-sg-F1:**<br>CCGCAGTTCTGCCCCAGATC<br>**Ds-9.28-sg-F1:**<br>GCGGCCCTGATTGTCAACGG
Dscam$^{\Delta 9.2\text{-}9.29}$	**Ds-9.1-F:**<br>TGCCAAGAATCAGGAAGGATACTC<br>**Ds-9.29-R:**<br>CTCCGCCGGGAACGGAGCAGTG	**Ds-9.2-sg-F1:**<br>CCGCAGTTCTGCCCCAGATC<br>**Ds-9.29-sg-F1:**<br>AGCGTCTTAGTATTAAGAGA
Dscam$^{\Delta 9.1\text{-}9.28}$	**Ds-9.1-F:**<br>TGCCAAGAATCAGGAAGGATACTC<br>**Ds-9.29-R:**<br>CTCCGCCGGGAACGGAGCAGTG	**Ds-9.1-sg-F1:**<br>CCGCAGGTCGTACCCTTTGA<br>**Ds-9.28-sg-F1:**<br>GCGGCCCTGATTGTCAACGG
Dscam$^{\Delta 9.1\text{-}9.29}$	**Ds-9.1-F:**<br>TGCCAAGAATCAGGAAGGATACTC<br>**Ds-9.29-R:**<br>CTCCGCCGGGAACGGAGCAGTG	**Ds-9.1-sg-F1:**<br>CCGCAGGTCGTACCCTTTGA<br>**Ds-9.29-sg-F1:**<br>AGCGTCTTAGTATTAAGAGA
Dscam$^{\Delta 9.2\text{-}9.30}$	**Ds-9.1-F:**<br>TGCCAAGAATCAGGAAGGATACTC<br>**Ds-9.30-R:**<br>GCCCCTATCTCATTTCCATTAT	**Ds-9.2-sg-F1:**<br>CCGCAGTTCTGCCCCAGATC<br>**Ds-9.30-sg-F1:**<br>GGGAATAACCACGTCCCGCG
Dscam$^{\Delta 9.1\text{-}9.30}$	**Ds-9.1-F:**<br>TGCCAAGAATCAGGAAGGATACTC<br>**Ds-9.30-R:**<br>GCCCCTATCTCATTTCCATTAT	**Ds-9.1-sg-F1:**<br>CCGCAGGTCGTACCCTTTGA<br>**Ds-9.30-sg-F1:**<br>GGGAATAACCACGTCCCGCG
Dscam$^{\text{Single9.1}*}$	**Ds-9.1-F:**<br>TGCCAAGAATCAGGAAGGATACTC<br>**Ds-9.33-R:**<br>ACTTGGTCTTTGGCCTACGGAT	**Ds-9.2-sg-F1:**<br>CCGCAGTTCTGCCCCAGATC<br>**Ds-9.33-sg-F1:**<br>GACCAGGCTAACTGTGAATG

Specific primers used for RT-PCR

Primers	5'-3' sequence	Assay
**Ds-3-F**	TGGATCAGGAGCGACGGTAC	**RT-PCR**
**Ds-5-R**	CTCCAGAGGGCAATACCAGG	**RT-PCR**
**Ds-5-F**	GCTACCAGTGCCGAACCAAACATC	**RT-PCR**
**Ds-7-R**	AGTCTCAACGCTTTCGCCTCCCAC	**RT-PCR**
**Ds-7-F**	AACATAACCTCGGTCCACGC	**RT-PCR**
**Ds-8-R**	GTCGCTTGGTCTGAGTTCCG	**RT-PCR**
**Ds-8-F**	ACTTGCGTTGCCAAGAATCAGGAAG	**RT-PCR**
**Ds-10-R**	GCCTTATCGGTGGGCTCGAGGATCC	**RT-PCR**
**Ds-10-RT**	GGTTTGGGGAAGCCATCAGCCTT	**RT**
