# Supplementary material for: A systematic CRISPR screen reveals redundant and specific roles for Dscam1 isoform diversity in neuronal wiring
Source: PLoS Biol. 2023 Jul 6;21(7):e3002197. doi: 10.1371/journal.pbio.3002197 (PMC10325099; doi:10.1371/journal.pbio.3002197)
Supplement: S2 Table — (PDF) [file pbio.3002197.s014.pdf]

Table S2 Summary of the mutation sequences of *Dscam1* mutants

Mutants	Mutation sequences*	Assay
Dscam$^{\Delta 4.10\text{-}4.12}$	TTCGTCGATCTCTGGCTGGACTCGGAGGGTCGCAACTACTATCCGAACAATGCCGCAGAGACGG**4.9**gtacttggccaggtttcttt~~gccgtagctctctcagg~~......~~cccaacccttttttagttt~~tcccctccaaaaatccatgggtcgct	Mutants of variable exon 4 cluster
Dscam$^{\Delta 4.1\text{-}4.5}$	gcacagccggcccgcagctagttggccgaaatgagttat~~ggtctacaccctgaacacgggctggcgtaaagaata~~......~~ttaagaagttagtcctaagcagcaatcccattccttccccat~~ttcaatttgcttcagcttctgtttcatttcaccactctaccccattttaaatcgcagTTGTGAATCAGTTTTACGGCGCCGATATCCTGATGGAGTATGTCATCAGGGGAAATGCGGCGGTTTTGAAA**4.6**	
Dscam$^{\Delta 4.6\text{-}4.12}$	CTCCGGAAGAGGATTTTG**4.5**gtacagtcccgcaccgttgaaactattaagaagttagtcctaagcagcaatccattcctt~~ccccatttcaatttgcttcagcttc~~......~~catcccaacccttttttagtttt~~cccctccaaaaatccatggg	
Dscam$^{\Delta 4.1\text{-}4.9}$	aatcggcc~~TGGTTGCCCAGTACTACGAGGCCGGATGTTAACAAGGAGCACGTTATAAGAGGCAATTCGG~~**4.1**......~~CGTCATTAGGGGCAACTCTGTGGTGATGAAGTGCCGAGATTCCCTCCTA~~CGTGGCCGACTTCGTGTTCGTCGA**4.9**	
Dscam$^{\Delta 4.1\text{-}4.10}$	cggcc~~TGGTTGCCCAGTACTACG~~**4.1**......TGGTGAAGCAGTTTTTCGAGTCGCAAGTCTACGACGAGTATGTGATCAAGGGCAATGCG**4.11**	
Dscam$^{\Delta 4.3\text{-}4.12}$	GCCTATGTAATACGCG**4.3**~~GCAATGCGGCCGGTTCTGAAATGCGAAATCCCCTCCTTTGT~~......AGTTATCTATCCCCAGGAGGATATCGCGGAAAGCG**4.12**	
Dscam$^{\Delta 6.36}$	cagagagcgctggaaagagagggagagtcgcggt......~~AAGCTCATCGAGGTGCAAAAGTCAAAGCGCACGCTTTTGCCCTGCTCTGCCAGCCCCAAGCCTTTCCAGTGCCCATC~~ATCAG**6.36**gtaagccccacattcaggcagttagta	Mutants of variable exon 6 cluster
Dscam$^{\Delta 6.21\text{-}6.30}$	CTCAAGGTTATCCAATGCCAGCGTTTAG**6.20**gtaaaaacattgttgagt......~~AAGGCTACCCCGGTGCCTGTGTTTA~~G**6.30**gtaatcaaagcggaaagat	
Dscam$^{\Delta 6.10\text{-}6.20}$	ACAAACTGCAGACACGCGAGATTT~~CCCAGGGAATCGGAATCGCTCTACTGTGTCCGGCGCAGTCATATCCGATTCCAGCCCACAG~~**6.10**......~~CAATGCCAGCGTTTAG~~**6.20**~~gtaaaaacattgttgagtacaatcctacag~~atcgggttt	
Dscam$^{\Delta 6.30\text{-}6.48}$	GCGCTTCTATGTCCGGCGCAAGG~~CTACCCCGGTGCCTGTGTTTAG~~**6.30**......~~atcatagggtcccccattactaggccgaggatagg~~ttctcccaaatccc	
Dscam$^{\Delta 6.10\text{-}6.30}$	AACCCGTTGGCTCTGTCAAGCCGAAAATAAATGTTCAAGACAAACTGCAGACACGC~~GAGATTTCCCAGGGAATCGGAATCGCTCTACTGTGTCCGGCGCAGTCATATCCGATTCCAGCCCACAG~~**6.10**.........GCCTGTGTTTAG**6.30**gta	
Dscam$^{\Delta 6.1\text{-}6.30}$	ggctattaactttgaactatatttcgcttcattcac~~agAACCAGTTGGCAGAGTTTCGCCCAAGTTTC~~**6.1**......CCGGTGCCTGTGTTTAG**6.30**gtaatc	
Dscam$^{\Delta 6.17\text{-}6.48}$	gtgagtaccagctg~~tcaggacttgtcccagcacttaggcagtaaatcgaaatcggcagatgccatgactaagctccccccctaaatctttgtagAACCTGTTGGCAGTGTGGCCCCCC~~**6.17**.........~~cccattactagg~~gatactagtaccgag	
Dscam$^{\Delta 6.2\text{-}6.35}$	GTCAAACCCTTCTATGCCCTGCCCAGGCTTATCCAGCTCCCCTTTTTAG**6.1**gtaggactaatatccatccagac......taatggacaggacaggatggctgtgcatgtgtgggtgcttcccctttccactttccactttttcgttgtgcac	
Dscam$^{\Delta 6.8\text{-}6.47}$	AACCCGTTGGCAGTGTGGCGCCCAAGTTGAACTTCTTAGAGCGCTTTAAGATGATGCAGATCCA~~GAGCCGAACTAGCTTCAACATTCAGTGTCCGGTACAATCGTATCCCATGCCTGTTTTCAA~~**6.8**......CCGCATTTAG**6.47**gtaatga	
Dscam$^{\Delta 6.5\text{-}6.44}$	aacctcctttaagAA~~CCCGTTGGCAGCGTTGGCCCAAAACTCACATCAGGCGACG~~**6.5**......~~AGCCCGTGGCCT~~CCAAGGCTCCAACCTTTGCTAC**6.44**	
Dscam$^{\Delta 6.7\text{-}6.47}$	tacgaaaatgaaacgaatttaacc~~ttgcagAACCAATTGGTAGCGTCAGTCCTAA~~**6.7**......~~ATCGTTTCCCACACC~~CGCATTTAG**6.47**GTAATGAAAGCT	
Dscam$^{\Delta 6.4\text{-}6.44}$	acagAACCGGTC~~GGAAGTGTAAGTCCCCAGCTCAGTGGCAATGGTAATCA~~**6.4**......TCCATAGAACCAAGGCTCCAACCTTTGCTACGGCTTCAAAG**6.44**	
Dscam$^{\Delta 6.6\text{-}6.47}$	AGCCTGTTGGCACAGTCAGCCCCAAGATAAG~~TACTGGTGAGGACTTCAAGCACCTTAAGGGAAAGAGCAGCCAG~~**6.6**......~~TTCCCACACCCCG~~CATTTAG**6.47**gtaatgaaagctatttatcagagccaacattcaggcagcaaatagcat	
Dscam$^{\Delta 6.3\text{-}6.44}$	aattaaatattgggtttccctcttcgaattcccagAGC**6.3**......AGTATCAGAGGCTCCAACCTTTGCTACGGCTTCAAAGATTTCCAGTCTACTCG**6.44**	
Dscam$^{\Delta 6.5\text{-}6.47}$	ACATCAGGCGACGATTCGC~~GGACGGTGAGGATTAGGCAGGAGGATAGTGTTACGCTCCTTTG~~**6.5**......~~CGTTTCCCACACCCCGC~~ATTTAG**6.47**gtaatg	
Dscam$^{\Delta 6.1\text{-}6.44}$	atttcgcttcattca~~cagAACCAGT~~......~~agAGCCCCGTGGCCTCCAAGGCTCCAACC~~TTTGCTACGGCTTCAAAGATTTCCAGTCTACTCGGCTCGAGTTCGAGTGATATTGTGCTACTTTGCCAGGCTCAGGCTTTTCCAGTTCCCT**6.44**	
Dscam$^{\Delta 6.4\text{-}6.47}$	ccgaattcaacagAACC~~GGTCGGAAGTGTAAGTCCCCAGCTCAGTGGCAA~~......~~ATATTGTGCTTGGGACAATCGTTTCCCACACC~~CGCATTTAG**6.47**gt	
Dscam$^{\Delta 6.3\text{-}6.47}$	TCTGGCCGCAAGTGCAACTCTTCTCT~~GCCCGGCTCAGGCTTATCCGGTGC~~......~~CCATATTGTGCTTGGGACAATCGTTTCCCACA~~CCCGCATTTAG**6.47**g	

Dscam$^{\Delta 6.2\text{-}6.47}$	CTCCCCTTTTTAG**6.1**gtaggactaatatccatccagacccaga~~ccaacttgcgccagtttccaattgagattgctcgcc~~......~~aatgaaagctatttatcagag~~TATATGTATCccaacattcaggcagcaaatagcatttcaaatagggatcttacagaattttgctttgatttcagAACCCATTGGTAGTGTAG**6.48**	
Dscam$^{\Delta 9.7}$	TGACCTGCAAGTCAATG**9.6**gttggtgaaactcccttttggatctctctatatatccgcctg~~taaagttgcttcctacta~~......~~TGAATACGTTTCCACACTAG~~ATGTCAACG**9.7**gtttgtttgaccttatcactatttgtagtgctttaa	Mutants of variable exon 9 cluster
Dscam$^{\Delta 9.30\text{-}9.33}$	TGGTCAGGCAATCTCGGAGGATCTGGGAATAACCACGT~~CCCCCGTGGTCGTCGTGGAAGTGTGCTAACCATC~~**9.30**......~~CGAACTACAGTTGTCGGCCGATCAACCAGGTGGCCGTGGCAGAACGCCCGACCA~~TAACTGTGAATG**9.33**g	
Dscam$^{\Delta 9.10\text{-}9.18}$	TGTAGCTGAATTACAAGTTAATG**9.9**gtttgtccccacaagaaatattgaataaggagaaataagagcacgcttactgtctatgttgttttcacttagtattgcttg......CTTCCCtggcagggaaacccttgtccataagttatataatt	
Dscam$^{\Delta 9.24\text{-}9.33}$	ATCGAGTGGCTGTTCAACGGGGAATCCATAAGC~~TACCCCTCGGGAATCGCTGTTTTGCGAGGTGGCAAAAGGACTAGTGTACTCA~~**9.24**......~~AGAACGCCCGACCAGGC~~TAACTGTGAATG**9.33**gtaaatgggcggttatcagattatc	
Dscam$^{\Delta 9.14\text{-}9.24}$	GCGTCCTTTGATTTCGGGGAAGAGCCTCTGAACTACGGAGAACC~~TCCGTCAGTACAGTGCACCATTTTGGCTGGAGAC~~**9.14**......~~GAATCCATAAGCTACCCCTCGG~~GAATCGCTGTTTTGCGAGGTGGCAAAAGGACTAGTGTAC**9.24**	
Dscam$^{\Delta 9.1\text{-}9.14}$	cgaatacccaaacctctcgctcagTTCCACCG~~CAGGTCGTACCCTTTGATTTCGGTGAGGAAACCATCAACATGAATGACATG~~**9.1**......~~CGTGGAGACCTGCCCATGAATGTC~~ACCTGGTTGCTGAATAACGCCACCATTGATAGTT**9.14**	
Dscam$^{\Delta 9.14\text{-}9.29}$	TGACCTGACAGTGAACG**9.13**gtactgaaattgagtgtgtcatgatttagttttagtggttgcggttactaaaggtcttgctagttgttgctactaatcaaacccatgcccatttctctaactttctatctaagta......TTAAGTATTAagtattaagagaagctttcgttgatgcggttatg	
Dscam$^{\Delta 9.14\text{-}9.33}$	taatcaaacccatgcccatttctctaactttctatctaactaagc~~ctgcgtggataact~~......~~CCAGGTGGCCGTGGCAGAACGCCCGACCAGCC~~TAACTGTGAATG**9.33**gtaaatgggcggttatcagattatcagatatgccaattcc	
Dscam$^{\Delta 9.2\text{-}9.24}$	cctactcgcatcctcgccctcctccg~~cagTTCTGCCCCCAGATCGTGCCCT~~**9.2**......~~ATTTGCCCATTGACATCGAGTGGCTGTTCAACGGGGAATCCATAAGCTACCCCT~~CGGGAATCGCTGTTTTGCGAGGTGGCAAAAGGAC**9.24**	
Dscam$^{\Delta 9.1\text{-}9.24}$	ttggtcgtccatcgtgcttctctcaatcgaatacccaaacctctcgctcagTTCCA**9.1**......CGCCTCGGGAATCGCTGTTTTGCGAGGTGGCAAAAGGACTAGTGTACTCACGATAGATTCCGTTCATGCGGGACATGCGGGGA**9.24**	
Dscam$^{\Delta 9.1\text{-}9.25}$	tcgaatacccaaacctctcgctcagTTCCACCGGTCGAC~~CAGGTCGTACCCTTTGATTTCGGTGAG~~**9.1**......~~ATACCACCGAACTA~~ATAGTTAATG**9.25**g	
Dscam$^{\Delta 9.2\text{-}9.27}$	GAGCTGCGCGTTAACG**9.1**gttcgcttaggccttcttcgattcgtagattgattagttccttggtttttgagttctgttttcttgaccctactcgcatcctcgccctcctc~~cgcagTTCTGCCCCAGATCGTGC~~......~~gtacttgcctgcgaagtcagagcttc~~tgtacagcacttcagtgtcgttccgggttatttagt	
Dscam$^{\Delta 9.2\text{-}9.28}$	TGCGCGTTAACG**9.1**gttcgcttaggccttcttcgattcgtagattgattagttccttggtttttgagttctgttttcttgaccctactcgcatcctcgccctcct~~ccgcagTTCTGCCCCAGA~~......~~gtgggatag~~agatccacatccata	
Dscam$^{\Delta 9.2\text{-}9.29}$	gagttctgttttcttgaccctaagagggccc~~ctcgcatcctcgccctcctccgcag~~......~~gttctcatcccagcg~~tcttagtattaagagaagctttcgttg	
Dscam$^{\Delta 9.1\text{-}9.28}$	cccaaacctctcgctcagTTCCACCGGTCGAC~~CAGGTCGTACCCTTTGATTTCGGTGAGGAAACCATCAACATGAATGACATGG~~**9.1**......~~AAATGGTGGAGTACACTGCGGCCCTG~~ATTGTCAACG**9.28**gtgggatagagatccacat	
Dscam$^{\Delta 9.1\text{-}9.29}$	aacctctcgctcag~~TTCCACCGCAGGTCGTACCCTTTGATTTCGGTGAGG~~**9.1**......~~gagaagctttcgttgatgcggttatg~~ttttaattttatgcacctt	
Dscam$^{\Delta 9.2\text{-}9.30}$	cttcgattcgtagattgattagttccttggtttttgagttctgttttcttgaccctactcgcatcctcgccctcctccg~~cagTTCTGCCCCAGATCGTGCCCTTCCA~~......~~ttaggaattctccggg~~attgggtcgctcgagtagtttctt	
Dscam$^{\Delta 9.1\text{-}9.30}$	ctctcgctcagTTC~~CACCGCAGGTCGTACCCTTTGATT~~**9.1**......~~CGATCTGCGAATAACCACGTCCCG~~CGTGGGTCGTCGTGGAAGTGTGCTAACCA**9.30**	
Dscam$^{\text{Single9.1}*}$	GGTCATCTACCACACGGCAGAGCTGCGCGTTAACG**9.1**gttcgcttaggccttcttcgattcgtagattgattagttccttggtttttgagttctgttttcttgaccctactcgcatcctcgccctcctc~~cgcagTTCTGCCCCAGATCGTGCCCTT~~......~~GATGACGTCACTCAG~~GCCACAGTTGGCGAACACACCAGCCTACTGCTGATCAACAGGGCGGGGGAGGGG**9.33**	

*The variable exon sequences are in capital letters and highlighted in green. Intron sequences are lowercase letters. The sequence in red font indicates the mutation sequence. The sequence shown in red font and strikethrough represents the deleted sequence.
