# Supplementary material for: A systematic CRISPR screen reveals redundant and specific roles for Dscam1 isoform diversity in neuronal wiring
Source: PLoS Biol. 2023 Jul 6;21(7):e3002197. doi: 10.1371/journal.pbio.3002197 (PMC10325099; doi:10.1371/journal.pbio.3002197)

Raw images for Figure 1B-C, 6F-G, S10A

Figure 1B

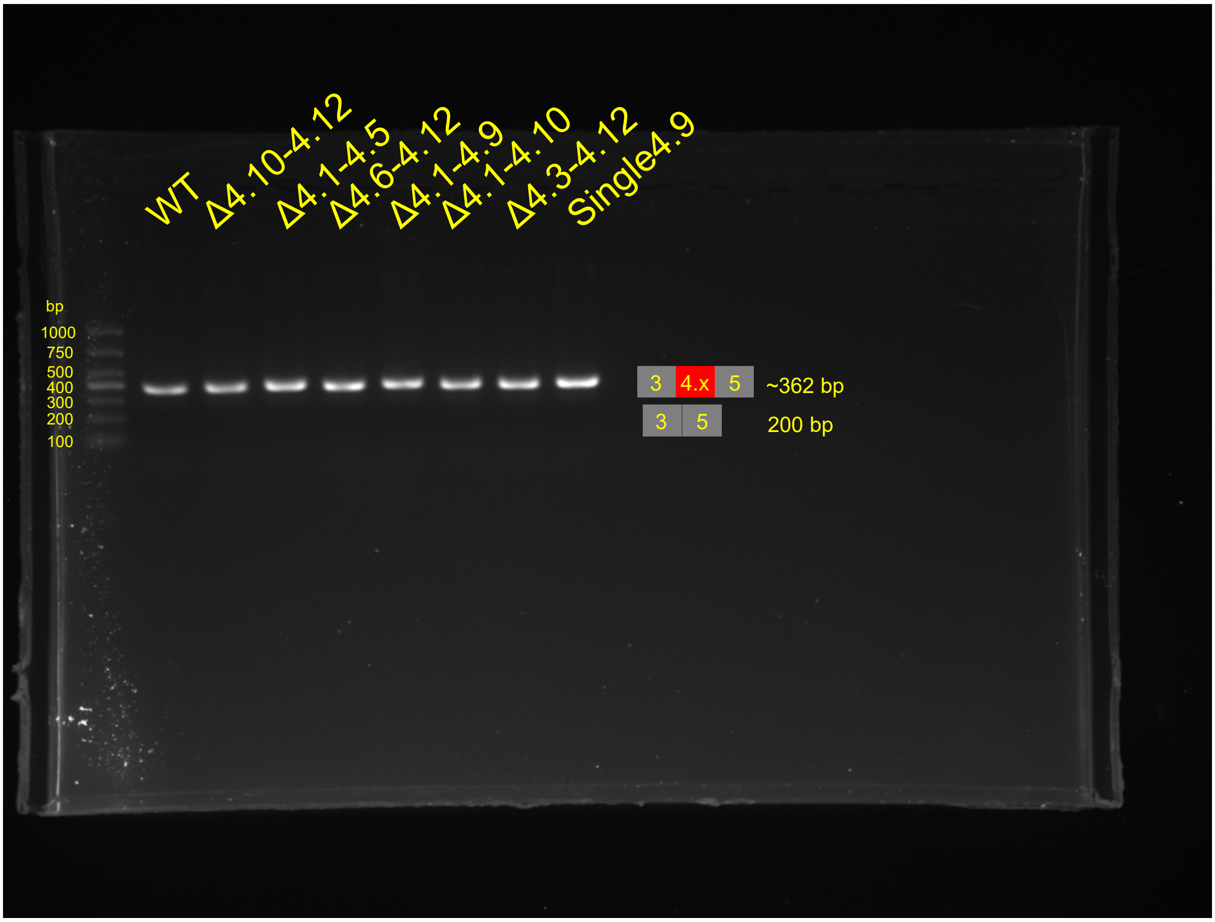
WT
Δ4.10-4.12
Δ4.1-4.5
Δ4.6-4.12
Δ4.1-4.9
Δ4.1-4.10
Δ4.3-4.12
Single4.9
bp
1000
750
500
400
300
200
100
3
4.x
5
~362 bp
3
5
200 bp

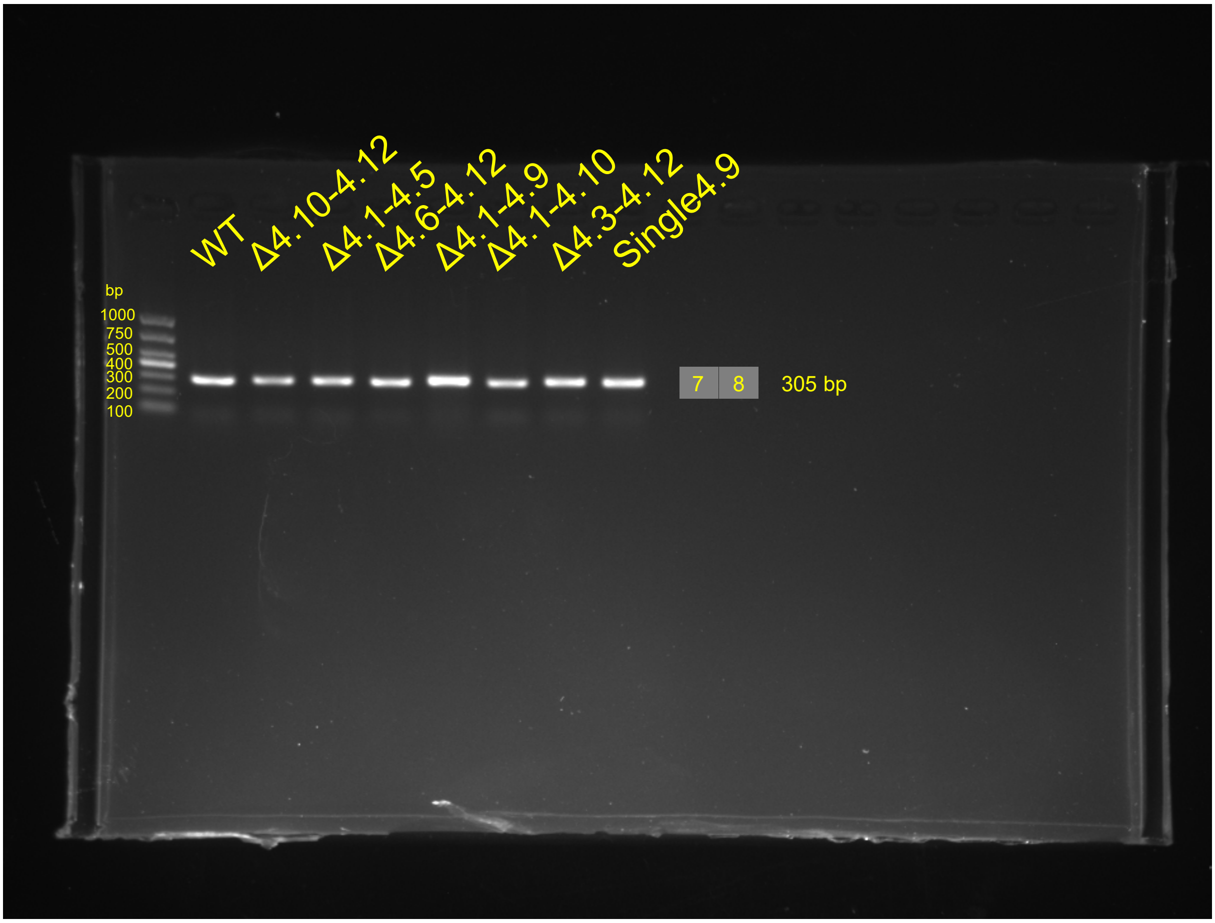
WT
Δ4.10-4.12
Δ4.1-4.5
Δ4.6-4.12
Δ4.1-4.9
Δ4.1-4.10
Δ4.3-4.12
Single4.9
bp
1000
750
500
400
300
200
100
7
8
305 bp

Figure 1B

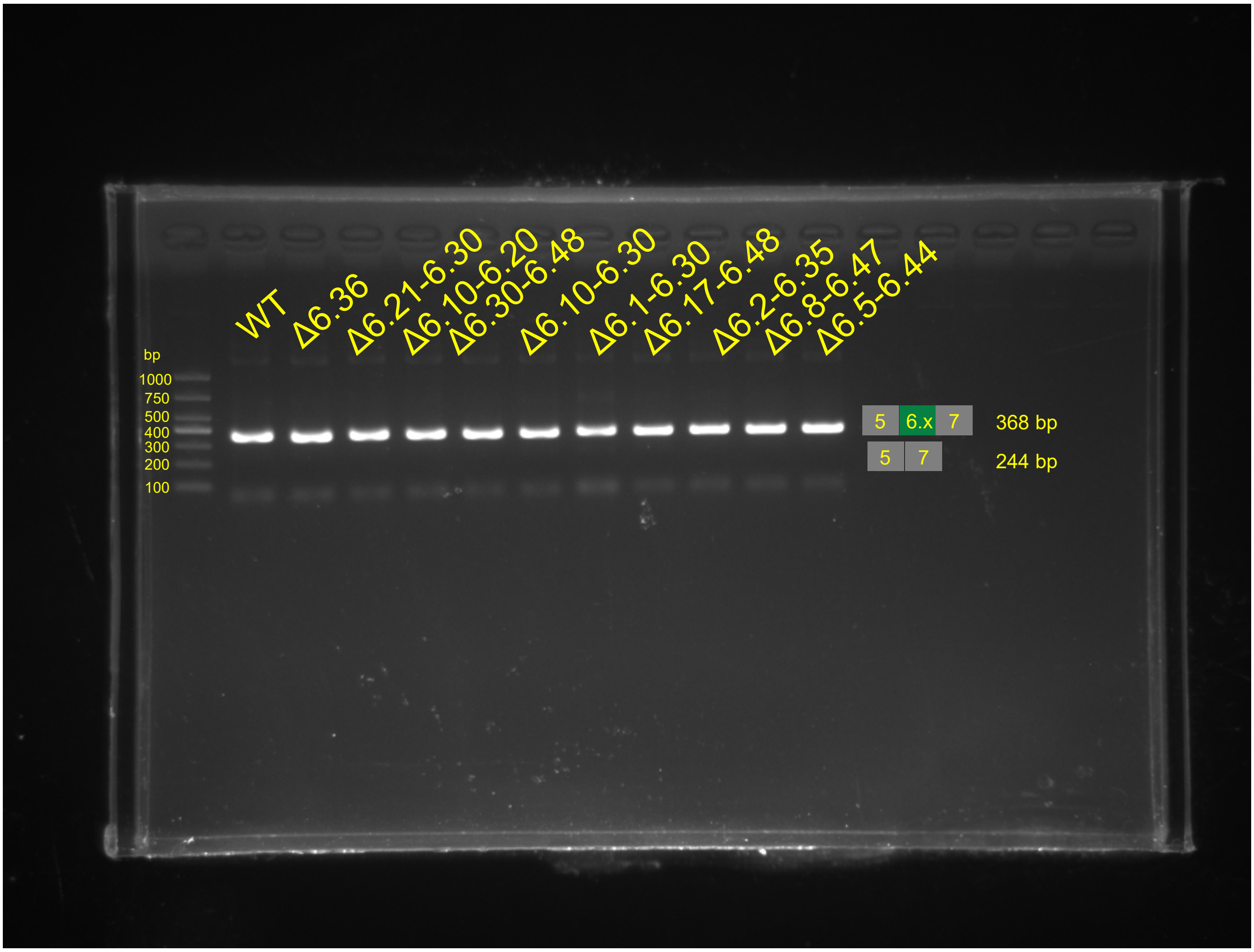
WT
Δ6.36
Δ6.21-6.30
Δ6.10-6.20
Δ6.30-6.48
Δ6.10-6.30
Δ6.1-6.30
Δ6.17-6.48
Δ6.2-6.35
Δ6.8-6.47
Δ6.5-6.44
bp
1000
750
500
400
300
200
100
5
6.x
7
368 bp
5
7
244 bp

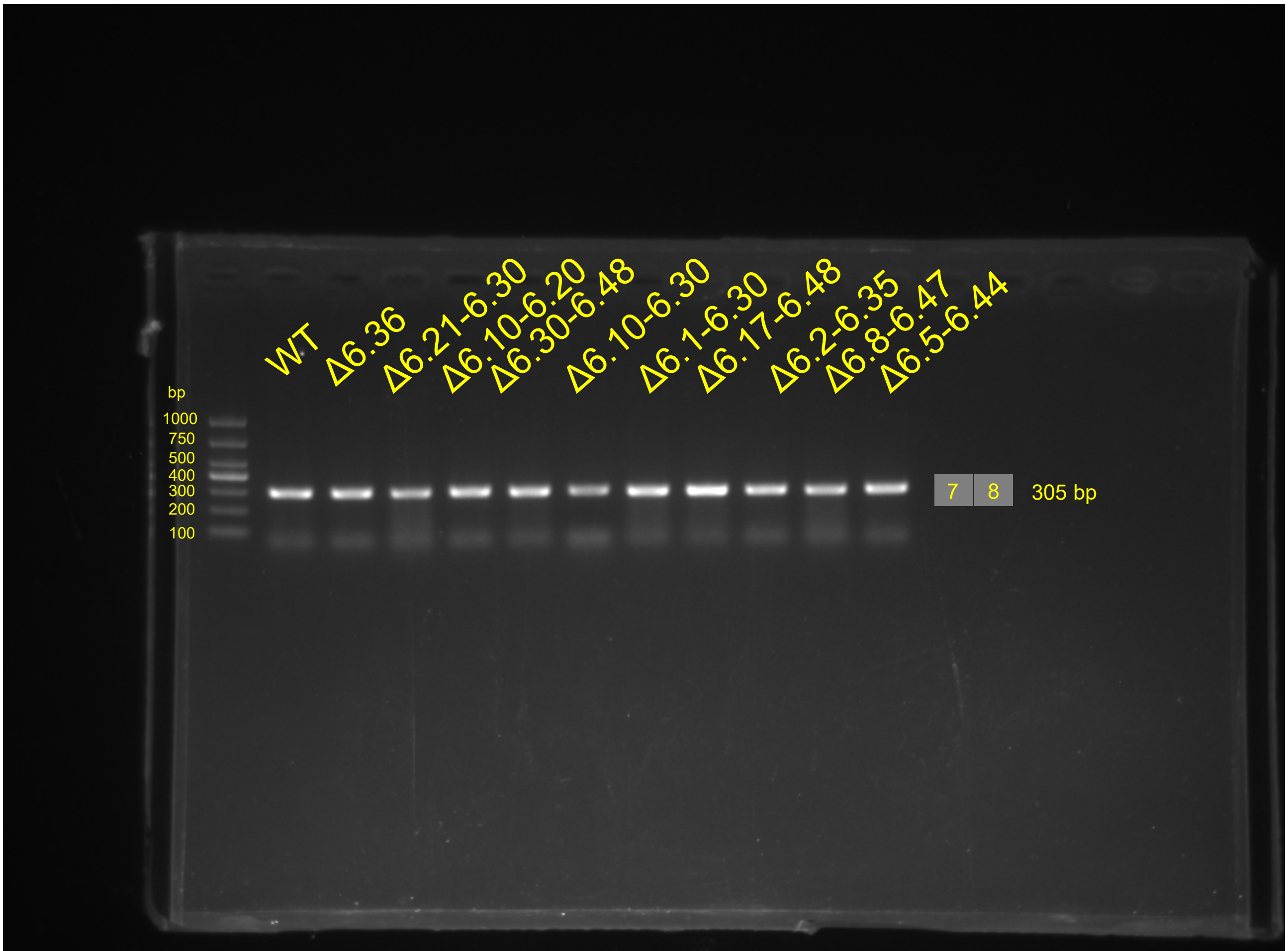
WT
Δ6.36
Δ6.21-6.30
Δ6.10-6.20
Δ6.30-6.48
Δ6.10-6.30
Δ6.1-6.30
Δ6.17-6.48
Δ6.2-6.35
Δ6.8-6.47
Δ6.5-6.44
bp
1000
750
500
400
300
200
100
7
8
305 bp

Figure 1B

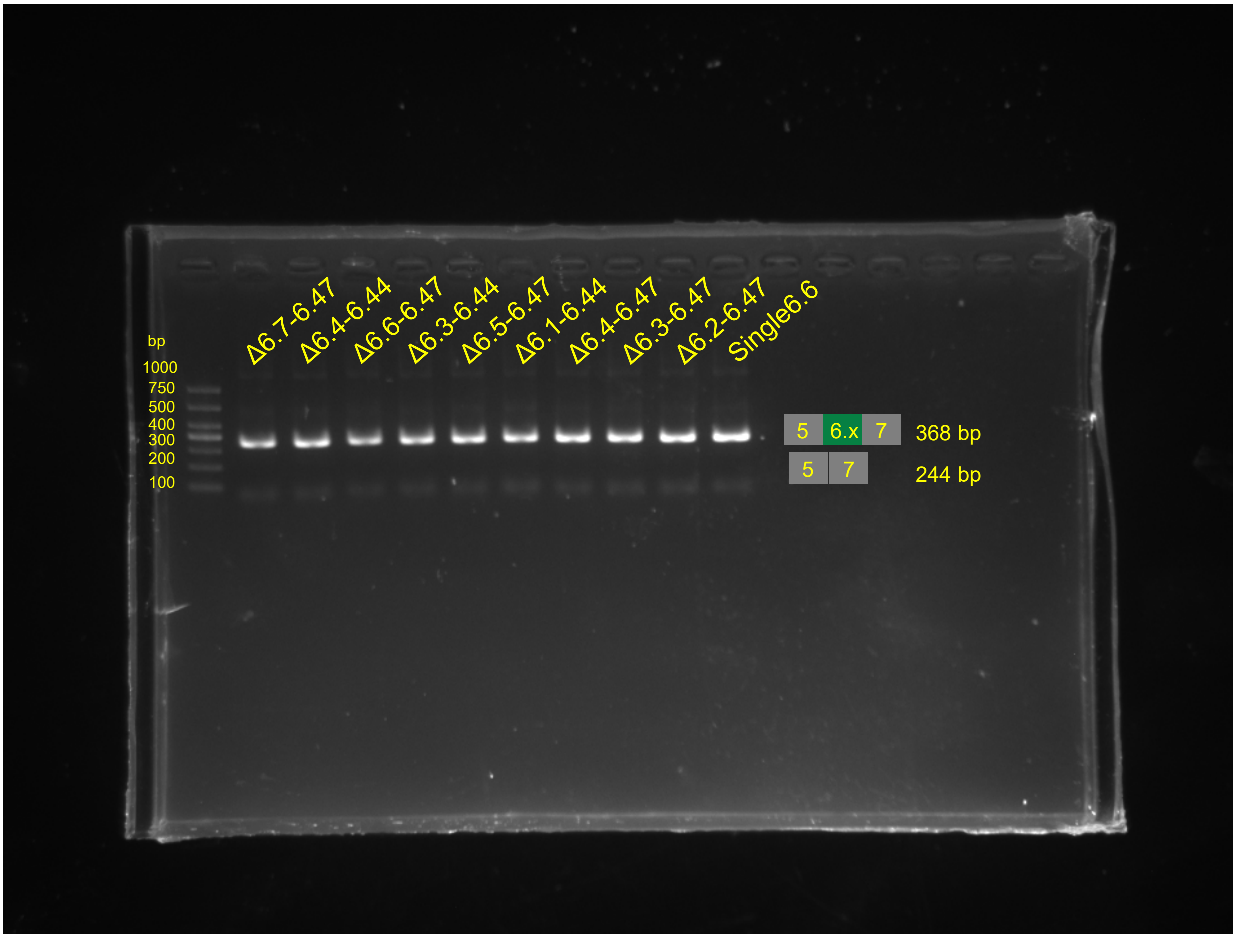
Δ6.7-6.47
Δ6.4-6.44
Δ6.6-6.47
Δ6.3-6.44
Δ6.5-6.47
Δ6.1-6.44
Δ6.4-6.47
Δ6.3-6.47
Δ6.2-6.47
Single6.6
bp
1000
750
500
400
300
200
100
5 6.x 7
368 bp
5 7
244 bp

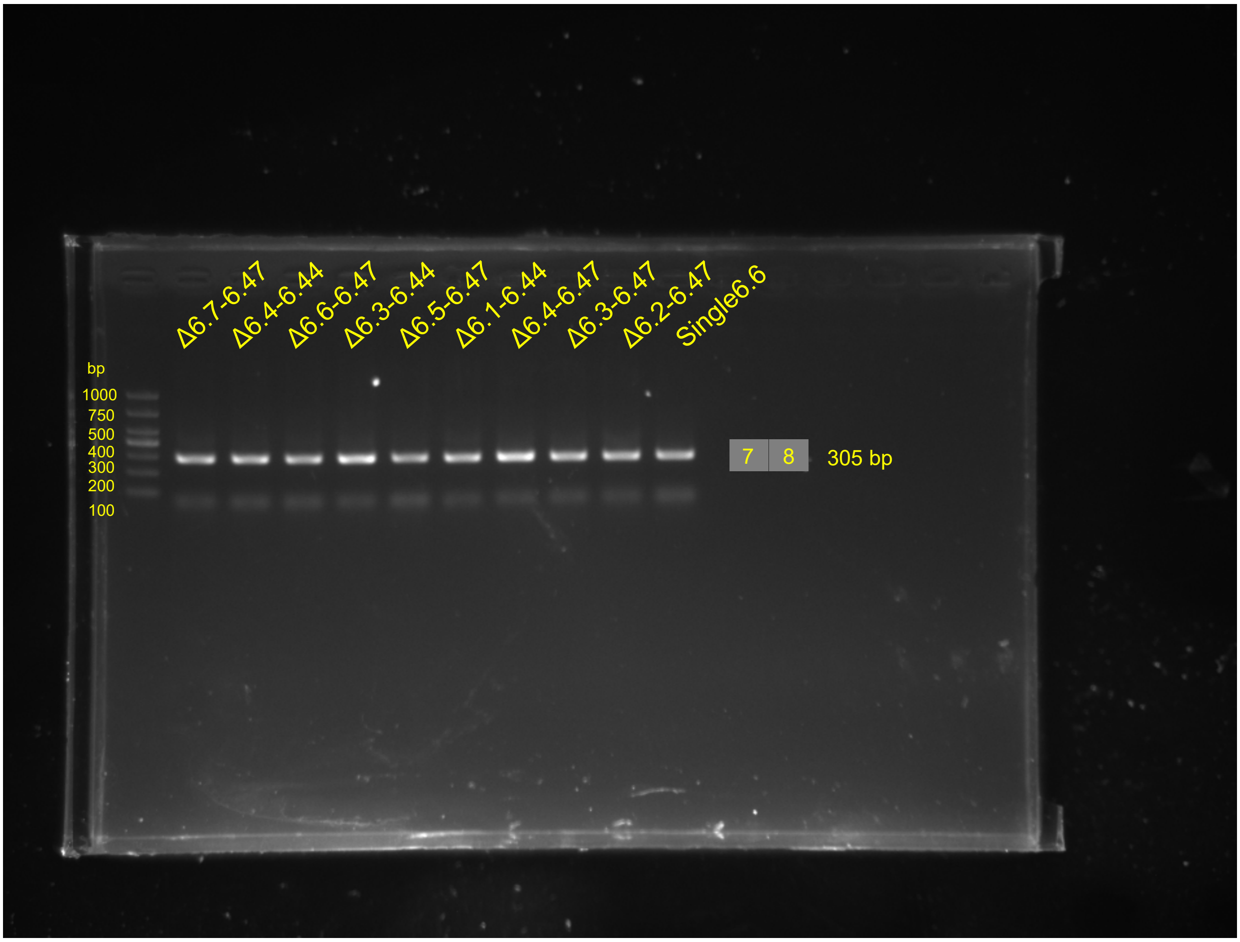
Δ6.7-6.47
Δ6.4-6.44
Δ6.6-6.47
Δ6.3-6.44
Δ6.5-6.47
Δ6.1-6.44
Δ6.4-6.47
Δ6.3-6.47
Δ6.2-6.47
Single6.6
bp
1000
750
500
400
300
200
100
7 8
305 bp

Figure 1B

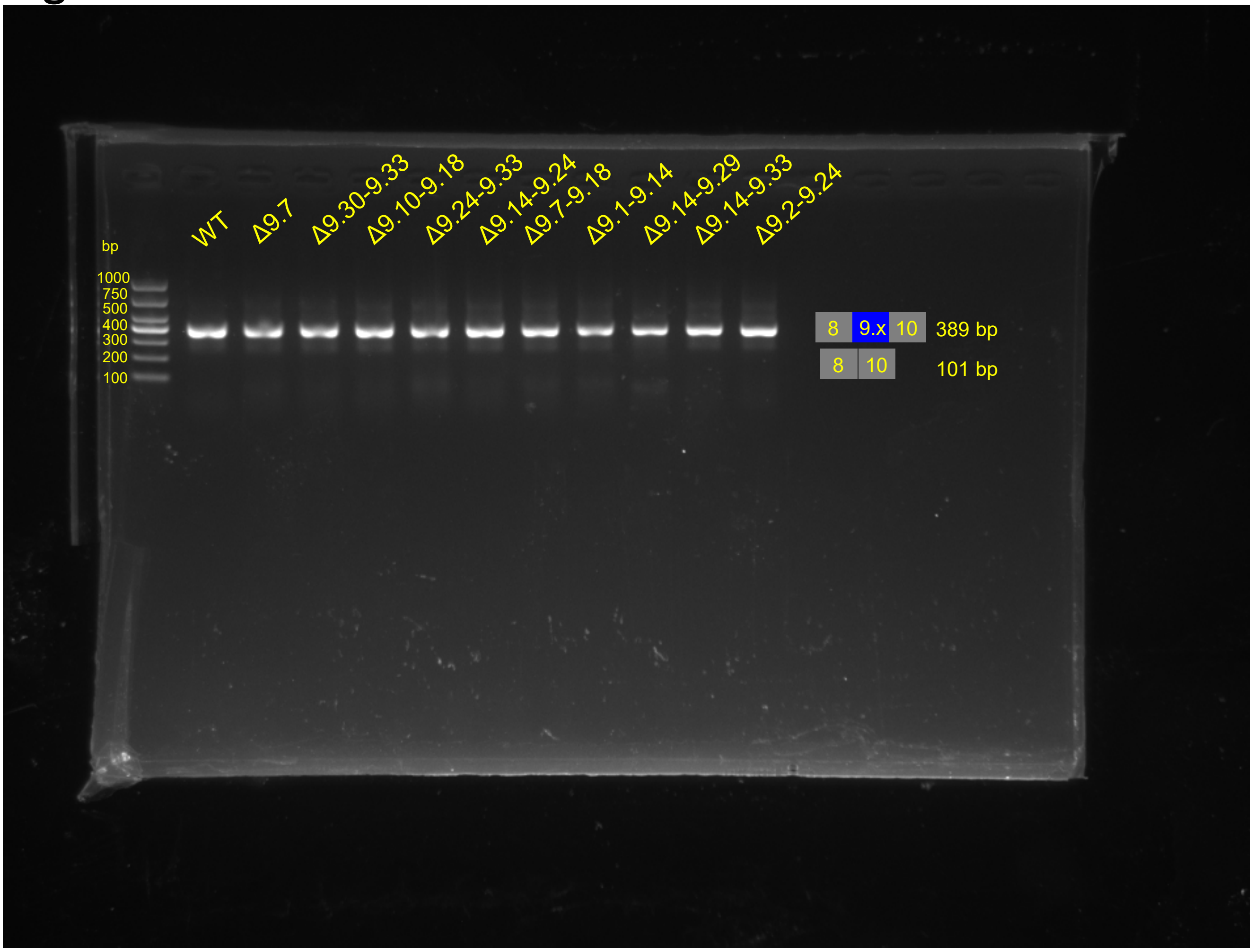
WT
Δ9.7
Δ9.30-9.33
Δ9.10-9.18
Δ9.24-9.33
Δ9.14-9.24
Δ9.7-9.18
Δ9.1-9.14
Δ9.14-9.29
Δ9.14-9.33
Δ9.2-9.24
bp
1000
750
500
400
300
200
100
8 9.x 10 389 bp
8 10 101 bp

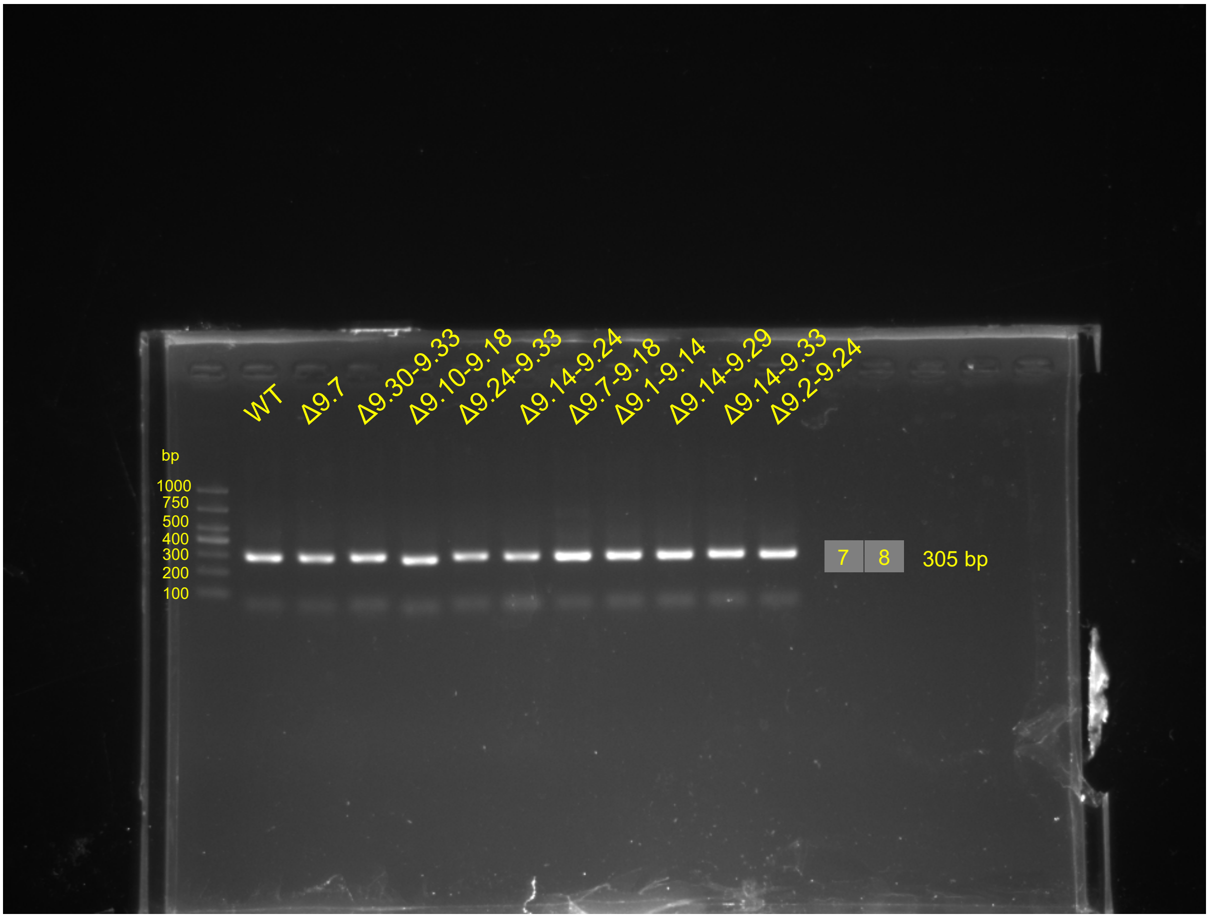
WT
Δ9.7
Δ9.30-9.33
Δ9.10-9.18
Δ9.24-9.33
Δ9.14-9.24
Δ9.7-9.18
Δ9.1-9.14
Δ9.14-9.29
Δ9.14-9.33
Δ9.2-9.24
bp
1000
750
500
400
300
200
100
7 8 305 bp

Figure 1B

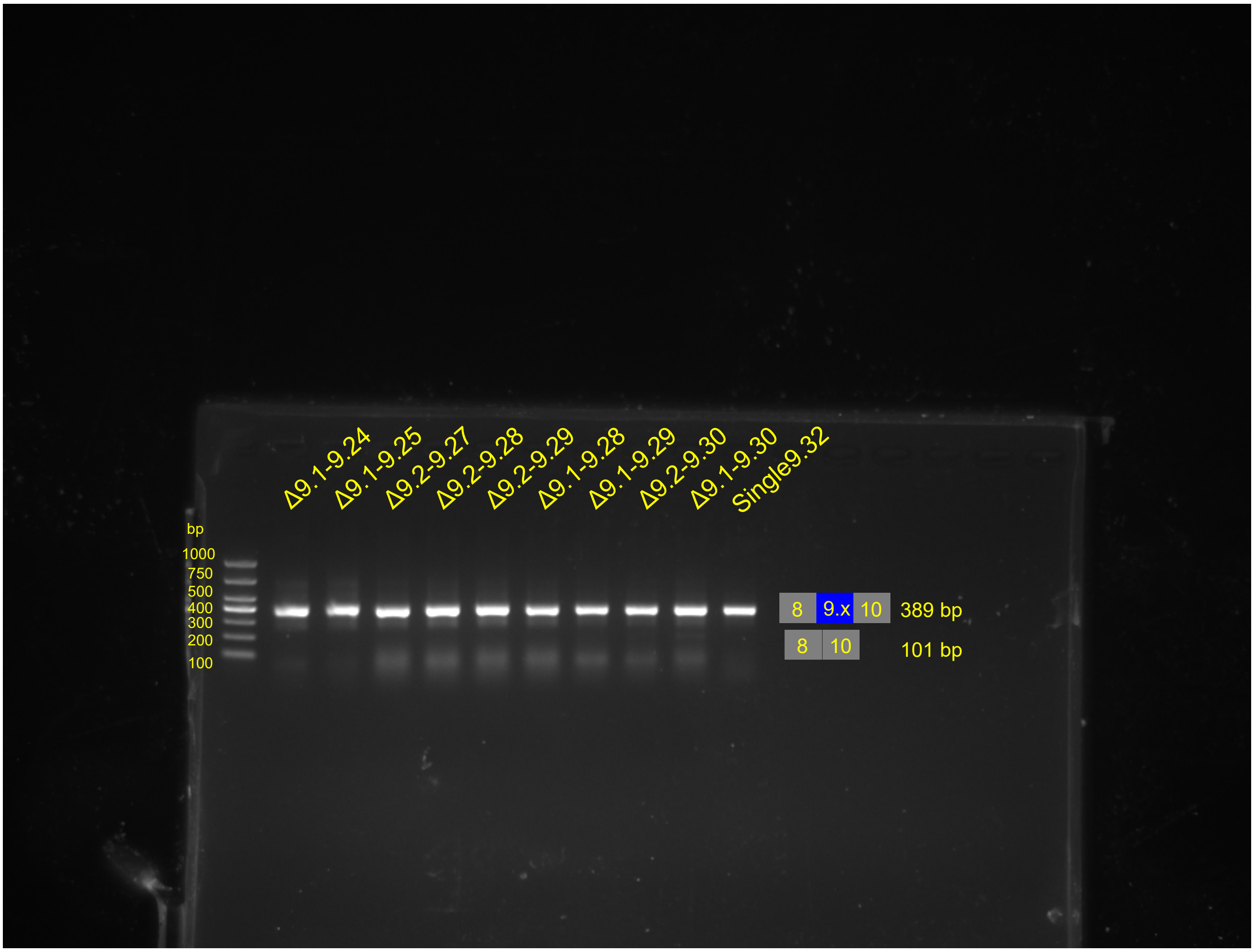
Δ9.1-9.24
Δ9.1-9.25
Δ9.2-9.27
Δ9.2-9.28
Δ9.2-9.29
Δ9.1-9.28
Δ9.1-9.29
Δ9.2-9.30
Δ9.1-9.30
Single9.32
bp
1000
750
500
400
300
200
100
8
9.x
10
389 bp
8
10
101 bp

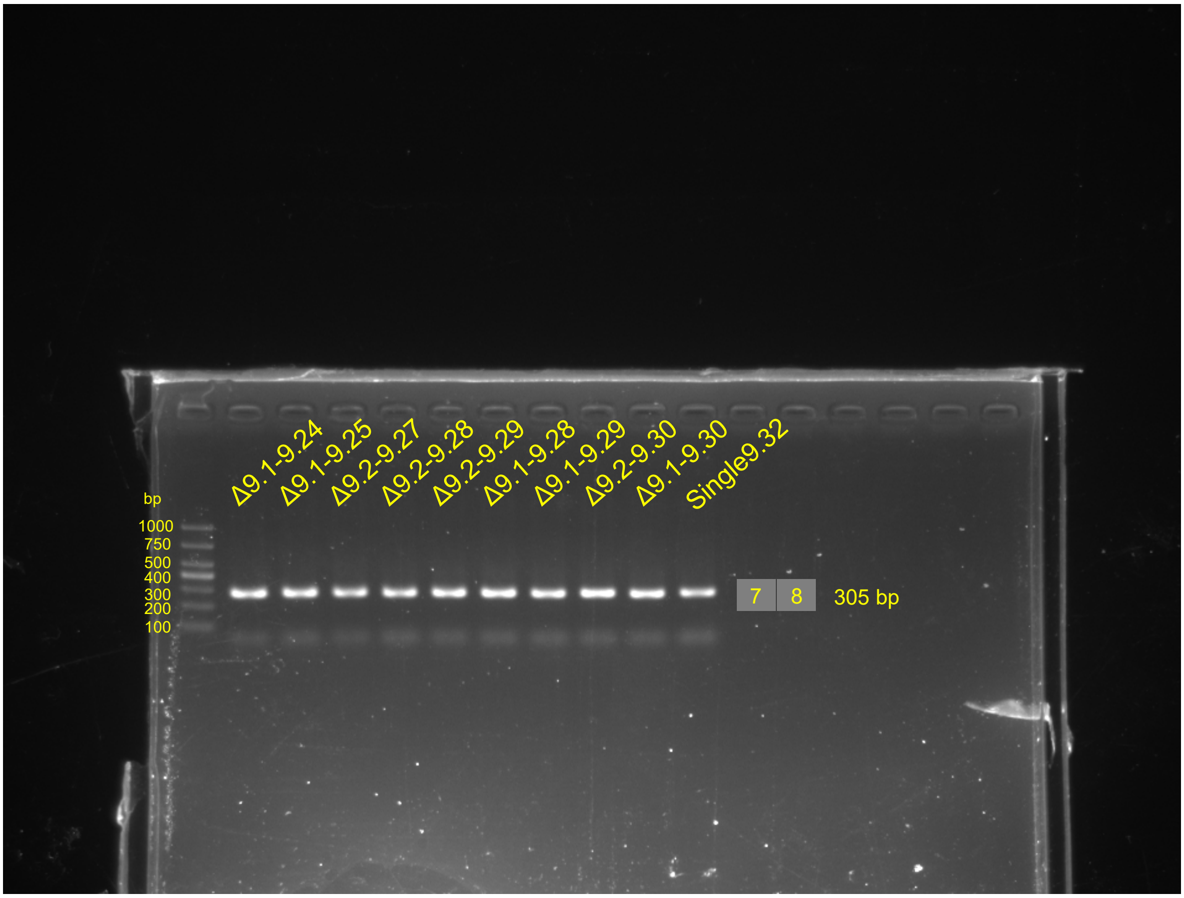
Δ9.1-9.24
Δ9.1-9.25
Δ9.2-9.27
Δ9.2-9.28
Δ9.2-9.29
Δ9.1-9.28
Δ9.1-9.29
Δ9.2-9.30
Δ9.1-9.30
Single9.32
bp
1000
750
500
400
300
200
100
7
8
305 bp

Figure 6F

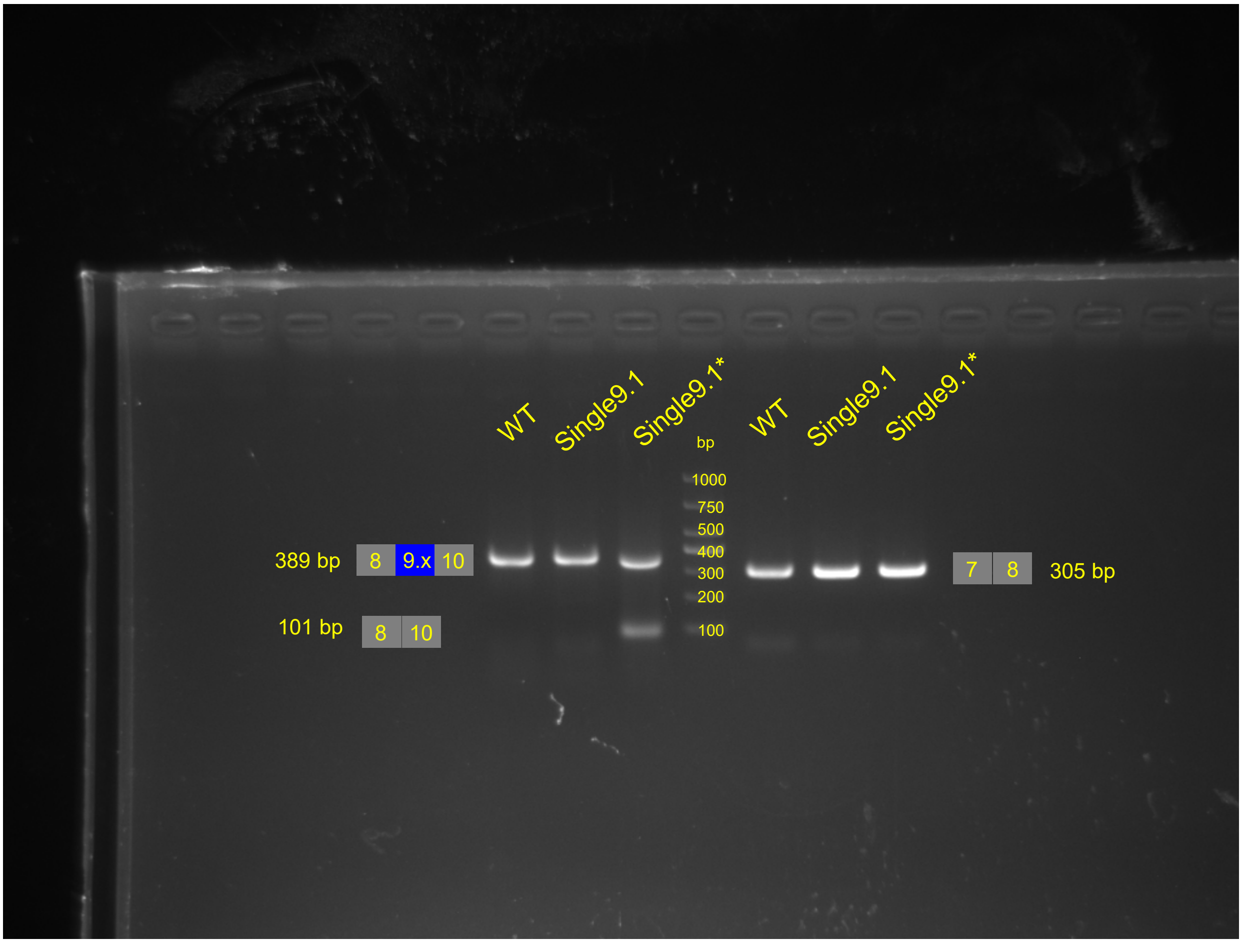
WT
Single9.1
Single9.1*
bp
WT
Single9.1
Single9.1*
1000
750
500
400
300
200
100
389 bp
8
9.x
10
101 bp
8
10
7
8
305 bp

Figure 1C

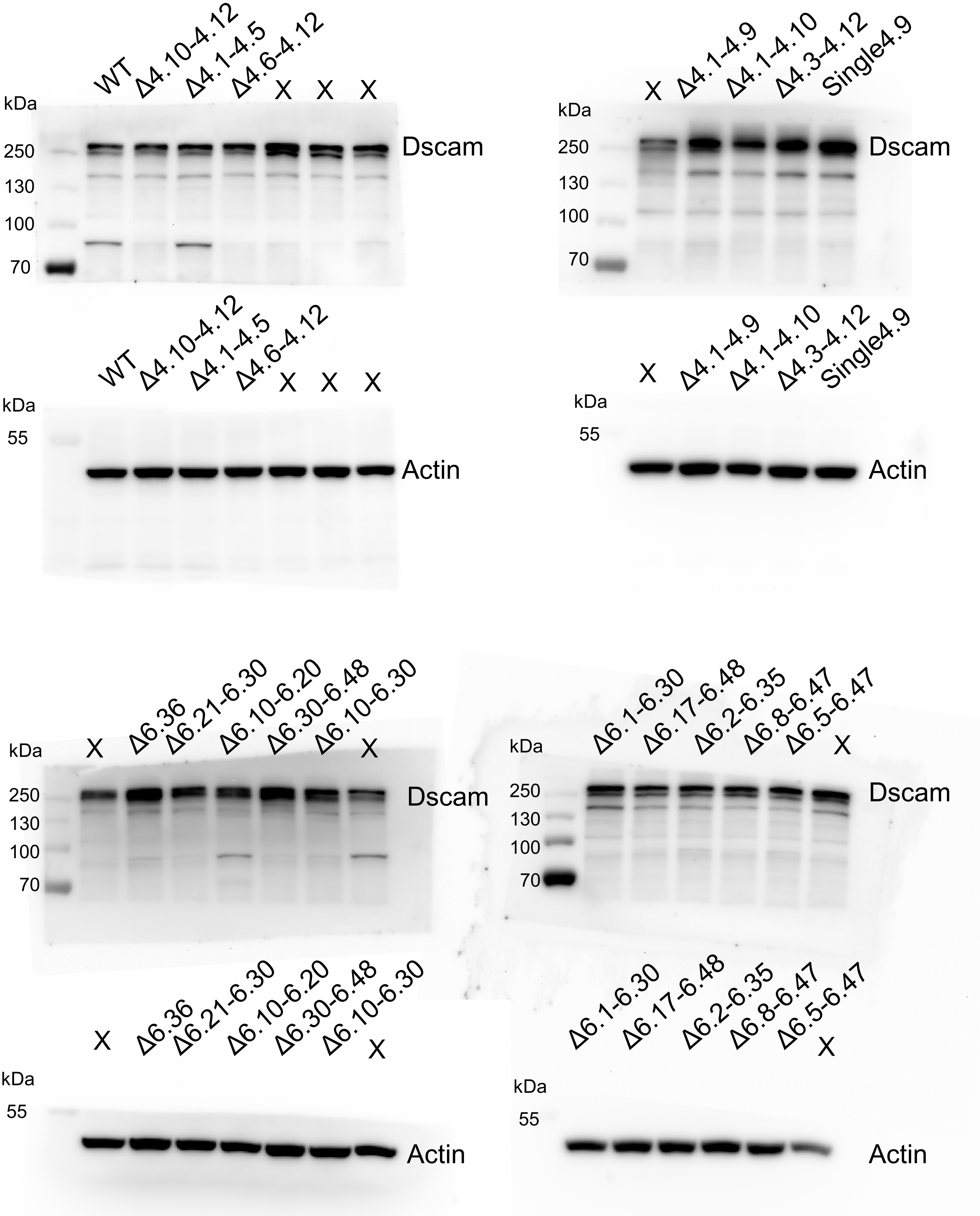
WT
Δ4.10-4.12
Δ4.1-4.5
Δ4.6-4.12
X
X
X
kDa
250
130
100
70
Dscam
X
Δ4.1-4.9
Δ4.1-4.10
Δ4.3-4.12
Single4.9
kDa
250
130
100
70
Dscam
WT
Δ4.10-4.12
Δ4.1-4.5
Δ4.6-4.12
X
X
X
kDa
55
Actin
X
Δ4.1-4.9
Δ4.1-4.10
Δ4.3-4.12
Single4.9
kDa
55
Actin
X
Δ6.36
Δ6.21-6.30
Δ6.10-6.20
Δ6.30-6.48
Δ6.10-6.30
X
kDa
250
130
100
70
Dscam
Δ6.1-6.30
Δ6.17-6.48
Δ6.2-6.35
Δ6.8-6.47
Δ6.5-6.47
X
kDa
250
130
100
70
Dscam
X
Δ6.36
Δ6.21-6.30
Δ6.10-6.20
Δ6.30-6.48
Δ6.10-6.30
X
kDa
55
Actin
Δ6.1-6.30
Δ6.17-6.48
Δ6.2-6.35
Δ6.8-6.47
Δ6.5-6.47
X
kDa
55
Actin

Figure 1C

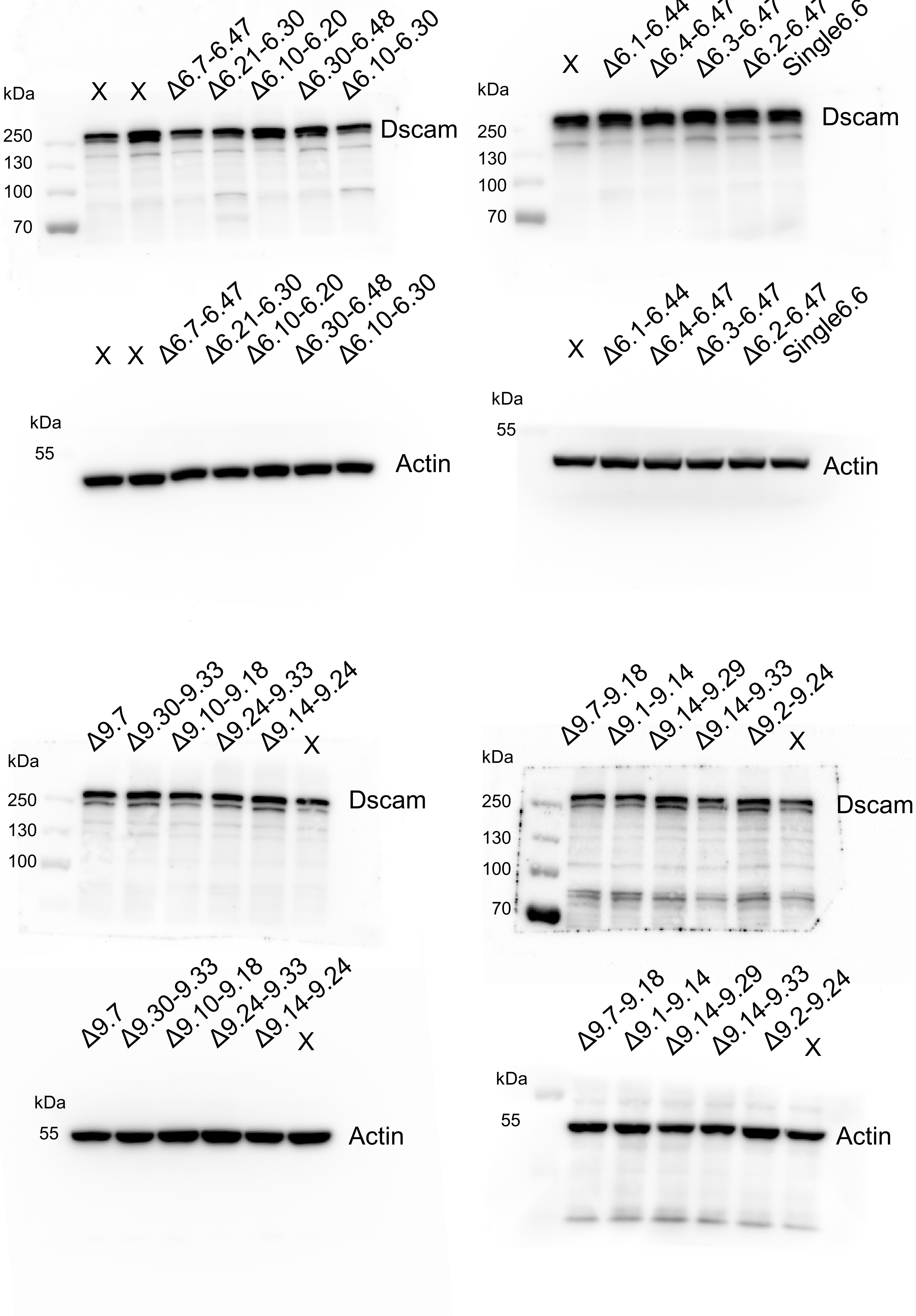
X
X
Δ6.7-6.47
Δ6.21-6.30
Δ6.10-6.20
Δ6.30-6.48
Δ6.10-6.30
kDa
250
130
100
70
Dscam
X
Δ6.1-6.44
Δ6.4-6.47
Δ6.3-6.47
Δ6.2-6.47
Single6.6
kDa
250
130
100
70
Dscam
X
X
Δ6.7-6.47
Δ6.21-6.30
Δ6.10-6.20
Δ6.30-6.48
Δ6.10-6.30
kDa
55
Actin
X
Δ6.1-6.44
Δ6.4-6.47
Δ6.3-6.47
Δ6.2-6.47
Single6.6
kDa
55
Actin
Δ9.7
Δ9.30-9.33
Δ9.10-9.18
Δ9.24-9.33
Δ9.14-9.24
X
kDa
250
130
100
Dscam
Δ9.7-9.18
Δ9.1-9.14
Δ9.14-9.29
Δ9.14-9.33
Δ9.2-9.24
X
kDa
250
130
100
70
Dscam
Δ9.7
Δ9.30-9.33
Δ9.10-9.18
Δ9.24-9.33
Δ9.14-9.24
X
kDa
55
Actin
Δ9.7-9.18
Δ9.1-9.14
Δ9.14-9.29
Δ9.14-9.33
Δ9.2-9.24
X
kDa
55
Actin

Figure 1C

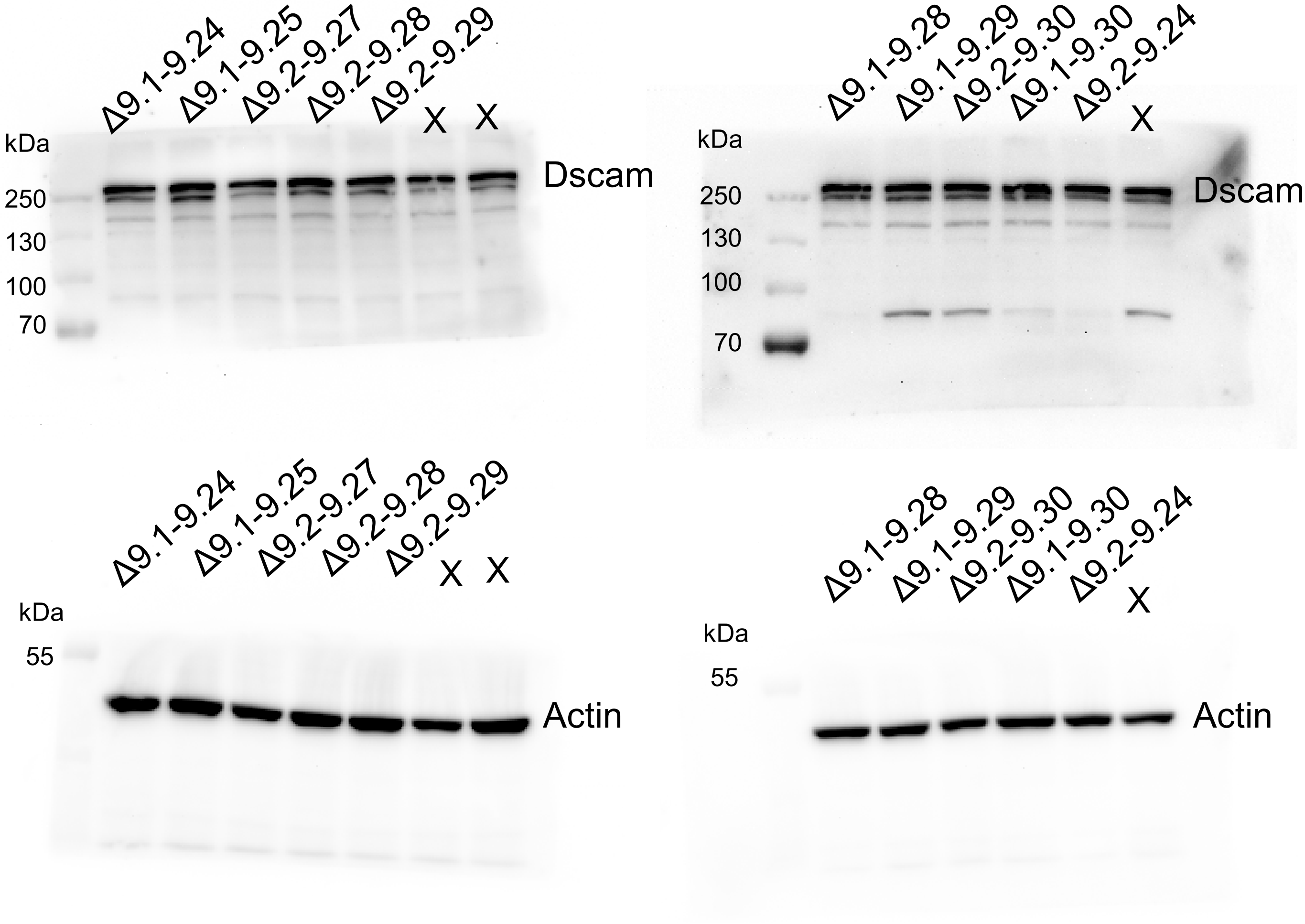
Δ9.1-9.24
Δ9.1-9.25
Δ9.2-9.27
Δ9.2-9.28
Δ9.2-9.29
X
X
kDa
250
130
100
70
Dscam
Δ9.1-9.28
Δ9.1-9.29
Δ9.2-9.30
Δ9.1-9.30
Δ9.2-9.24
X
kDa
250
130
100
70
Dscam
Δ9.1-9.24
Δ9.1-9.25
Δ9.2-9.27
Δ9.2-9.28
Δ9.2-9.29
X
X
kDa
55
Actin
Δ9.1-9.28
Δ9.1-9.29
Δ9.2-9.30
Δ9.1-9.30
Δ9.2-9.24
X
kDa
55
Actin

Figure S10A

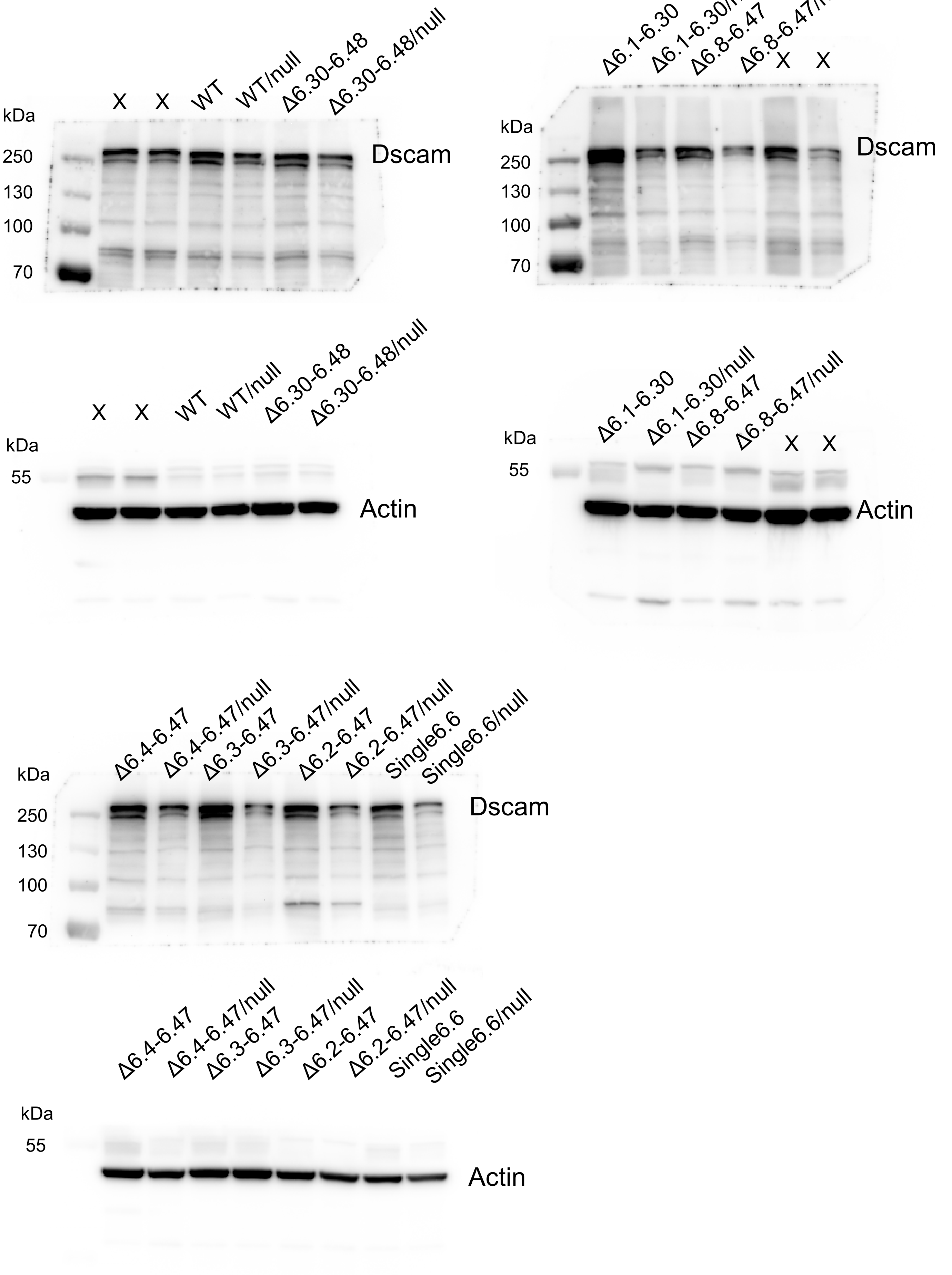

X
X
WT
WT/null
Δ6.30-6.48
Δ6.30-6.48/null
kDa
250
130
100
70
Dscam
Δ6.1-6.30
Δ6.1-6.30/null
Δ6.8-6.47
Δ6.8-6.47/null
X
X
kDa
250
130
100
70
Dscam
X
X
WT
WT/null
Δ6.30-6.48
Δ6.30-6.48/null
kDa
55
Actin
Δ6.1-6.30
Δ6.1-6.30/null
Δ6.8-6.47
Δ6.8-6.47/null
X
X
kDa
55
Actin
Δ6.4-6.47
Δ6.4-6.47/null
Δ6.3-6.47
Δ6.3-6.47/null
Δ6.2-6.47
Δ6.2-6.47/null
Single6.6
Single6.6/null
kDa
250
130
100
70
Dscam
Δ6.4-6.47
Δ6.4-6.47/null
Δ6.3-6.47
Δ6.3-6.47/null
Δ6.2-6.47
Δ6.2-6.47/null
Single6.6
Single6.6/null
kDa
55
Actin

Figure S10A，Figure 6G

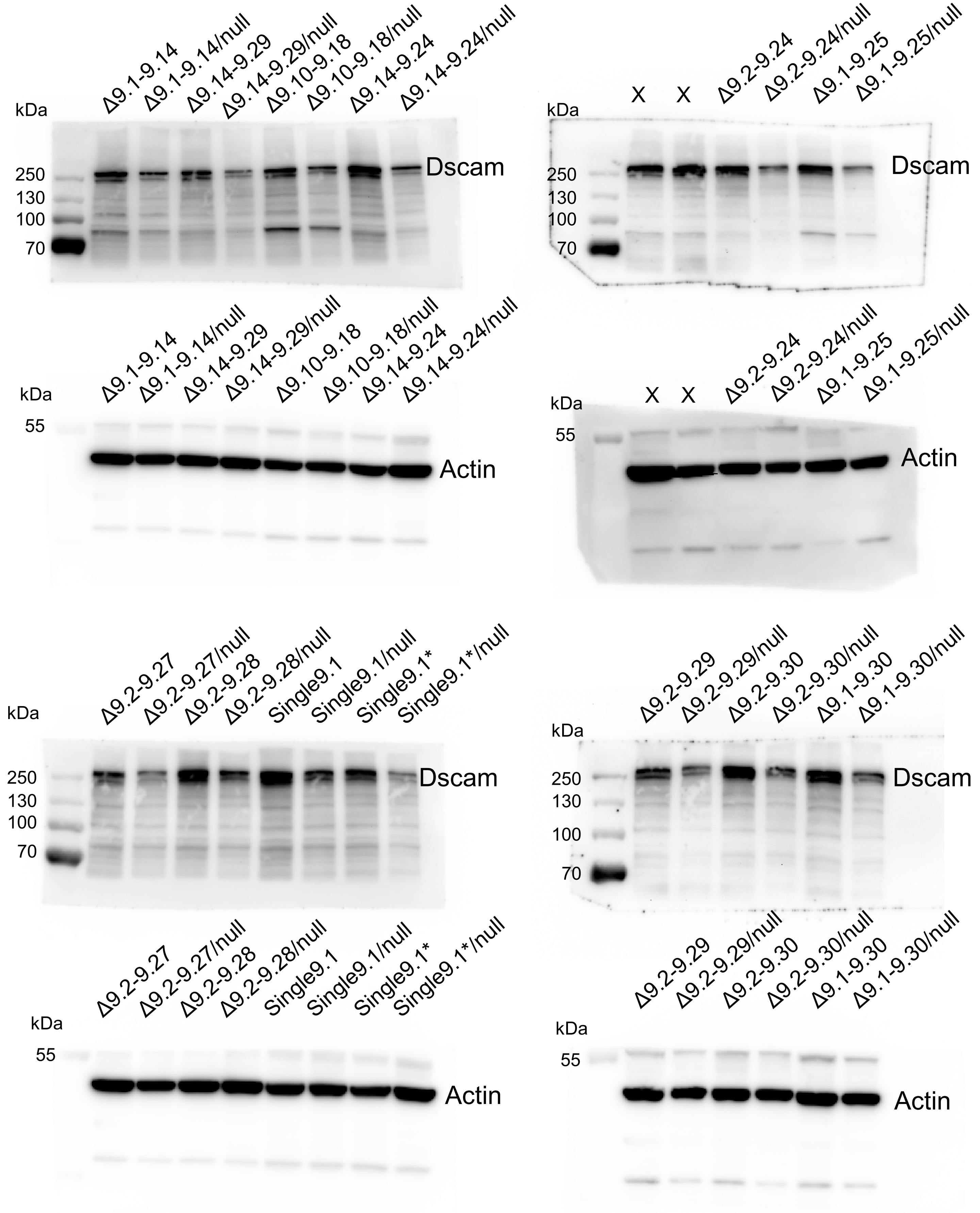

Supplement: S1 Raw Images — (PDF) [file pbio.3002197.s015.pdf]
